# Supplementary material for: Combinatorial Action of Temporally Segregated Transcription Factors
Source: Dev Cell. 2020 Nov 23;55(4):483–499.e7. doi: 10.1016/j.devcel.2020.09.002 (PMC7704111; doi:10.1016/j.devcel.2020.09.002)
Supplement: Document S1. Figures S1–S6 and Tables S1–S5 [file mmc1.pdf]

Developmental Cell, Volume *55*

Supplemental Information

Combinatorial Action of Temporally Segregated Transcription Factors

Julien Charest, Thomas Daniele, Jingkui Wang, Aleksandr Bykov, Ariane Mandlbauer, Mila Asparuhova, Josef Röhsner, Paula Gutiérrez-Pérez, and Luisa Cochella

Supplementary Information

Combinatorial action of temporally segregated transcription factors

Julien Charest[1], Thomas Daniele[1], Jingkui Wang[2], Aleksandr Bykov[2], Ariane Mandlbauer[2], Mila Asparuhova, Josef Röhsner, Paula Gutiérrez-Pérez and Luisa Cochella*

Research Institute of Molecular Pathology (IMP), Vienna BioCenter (VBC), Campus-Vienna-Biocenter 1, 1030 Vienna, Austria.

[1] Equal contribution

[2] Equal contribution

* Corresponding Author and Lead Contact: cochella@imp.ac.at

Supplementary Tables 1-5

Supplementary Figures 1-6

Supplementary Tables

	Heat shock T1				Heat shock T2			
	ASEL On	N	% ON	p-value	ASEL On	N	% ON	p-value
**zif-1**	78	84	93 ± 3	N/A	79	81	98 ± 2	N/A
**vhhGFP4::zif-1**	13	90	14 ± 4	<0.0001	81	85	95 ± 2	0.297

Table S1. ***lsy-6::yfp*** **expression in ASEL upon TBX-37 degradation (related to Figure 3)**

Number of animals scored, percentages and statistics for the experiments shown in **Fig. 3C**. YFP expression from the *lsy-6* locus was scored in the number of animals shown (N). YFP expression was either absent or observed exclusively in ASEL. Errors shown correspond to the standard error of the proportion. The proportions of animals expressing YFP were compared between experimental (nanobody-zif-1 line) and control (zif-1 alone) strains using a chi-squared test and p-values from these are shown for each of the two timepoints.

		lsy-6::gfp::Δtbs::5xUAS (lucIs41)				***lsy-6::gfp::5xUAS (lucIs54)***			
		**ASEL On**	**N**	**%ON**	**p-value**	**ASEL On**	**N**	**%ON**	**p-value**
		0	50	0 ± 0	N/A	50	50	100 ± 0	N/A
tbx-37p::gal4sk **(DBD alone)**	**lucEx959**	0	47	0 ± 0	N/A	50	50	100 ± 0	N/A
	**lucEx960**	0	60	0 ± 0	N/A				
	**lucEx961**	0	49	0 ± 0	N/A				
	**lucEx962**	0	54	0 ± 0	N/A				
tbx-37p::gal4sk::vp64 **(+ activator)**	**lucEx937**	61	67	91 ± 3	<0.0001				
	**lucEx938**	45	48	94 ± 3	<0.0001				
	**lucEx939**	44	51	86 ± 5	<0.0001				
	**lucEx940**	58	64	91 ± 4	<0.0001				
tbx-37p::gal4sk::unc-37 **(+ repressor)**	**lucEx1024**					3	44	7 ± 4	<0.0001
	**lucEx1025**					4	36	11 ± 5	<0.0001

Table S2. ***lsy-6::gfp*** **expression in ASEL upon GAL4-UAS tethering (related to Figure 5)**

Number of animals scored, percentages and statistics for the experiments shown in **Fig. 5B, C**. GFP expression from the *lsy-6* locus was scored in the number of animals shown (N) for each independent transgenic line. GFP expression was either absent or observed exclusively in ASEL. Errors shown correspond to the standard error of the proportion. The proportions of animals expressing GFP were compared using a chi-squared test and p-values from these are shown. The proportion in each independent experimental line (GAL4-VP64 or GAL4-UNC-37) was compared to tethering of the GAL4 DBD alone using the transgene *lucEx959*.

***lsy-2* Scoring**

	On	Dim	Off	N	% ON	% Dim	% OFF	p-value
otIs283 (lsy-6:yfp)	147	3	5	155	95 ± 2	2 ± 1	3 ± 1	N/A
otIs283, lsy-2 (ot64/+)	82	29	2	113	73 ± 4	26 ± 4	2 ± 1	<0.0001

delta Ebox Scoring

		On	Dim	Off	N	% ON	% Dim	% OFF	p-value
**wt Line 1**	**early emb**	24	0	2	26	92 ± 5	0 ± 0	8 ± 5	N/A
	**3-fold**	56	1	5	62	90 ± 4	2 ± 2	8 ± 3	N/A
**wt Line 2**	**early emb**	26	0	2	28	93 ± 25	0 ± 0	7 ± 25	N/A
	**3-fold**	58	0	0	58	100 ± 0	0 ± 0	0 ± 0	N/A
**ΔEbox Line 1**	**early emb**	16	3	3	22	73 ± 10	14 ± 7	14 ± 7	0.082
	**3-fold**	34	0	1	35	97 ± 3	0 ± 0	3 ± 3	0.210
**ΔEbox Line 2**	**early emb**	10	13	9	32	31.25	40.63	28.13	<0.0001
	**3-fold**	37	1	3	41	90.24	2.44	7.32	1
**ΔEbox Line 3**	**early emb**	18	12	8	38	47.37	31.58	21.05	0.0002
	**3-fold**	31	4	1	36	86.11	11.11	2.78	0.551

Table S3. *lsy-6::yfp* expression in ASEL in *lsy-2* mutants or upon E-box deletion (related to Figure 6)

Number of animals scored, percentages and statistics for the experiments shown in **Fig. 6B, C**. YFP expression from the *lsy-6* locus was scored in the number of animals shown (N). YFP expression was either absent or observed exclusively in ASEL. Errors shown correspond to the standard error of the proportion. The proportions of animals expressing YFP were compared using a chi-squared test and p-values from these are shown. Progeny of *lsy-2* heterozygous mothers were compared to wild-type animals carrying the same *lsy-6::yfp* fosmid reporter. For the E-box deletion experiment, the p-values for the comparisons between each independent experimental line (ΔEbox) to wild-type line 1 are shown (for each early embryos and 3-fold embryos); comparison to wild-type line 2 yielded lower p-values in all cases.

Table S4. Experiment Models: Organisms/Strains used in this study (related to STAR Methods and Key Resources Table)

Experimental Models: Organisms/Strains		
Caenorhabditis elegans: Wild type Bristol isolate	Caenorhabditis Genetics Center	WB Strain: N2
Caenorhabditis elegans: tbx-37(luc41[GFP::flex::tbx-37]) tbx-38(tm581) III	This study	MLC813
Caenorhabditis elegans: tbx-37(tm314) tbx-38(luc54[GFP::flex::tbx-38]) III	This study	MLC893
Caenorhabditis elegans: otIs252[lsy-6::YFP (fosmid); rol-6(su1006)] II; otIs220[gcy-5p::mChopti; rol-6(su1006)] IV	Hobert Lab Columbia University	OH9241
Caenorhabditis elegans: otIs220[gcy-5p::mChopti; rol-6(su1006)] IV; lsy-6(luc160[lsy-6::Δ150]) V	This study	MLC2219
Caenorhabditis elegans: lsy-6(luc157[lsy-6::YFP]), otIs235[che-1p::mChopti; rol-6(su1006)] V	This study	MLC2183
Caenorhabditis elegans: otIs232[che-1p::mChopti; rol-6] II; lsy-6(luc156[lsy-6::yfp::d150]) V	This study	MLC2311
Caenorhabditis elegans: lsy-6(luc157[lsy-6::YFP]), otIs235[che-1p::mChopti; rol-6(su1006)] V; otEx5161[hsp-16p::tbx-37; elt-2p::dsRed]	This study	MLC2203
Caenorhabditis elegans: lucSi100[hsp-16.41p::vhhGFP4::zif-1::SL2::mCherry::his-11::tbb-2 3'UTR] otIs252[lsy-6::YFP (fosmid); rol-6(su1006)] II; tbx-37(luc41[GFP::flex::tbx-37]) tbx-38(tm581) III	This study	MLC1300
Caenorhabditis elegans: lucSi102[hsp-16.41p::zif-1::SL2::mCherry::his-11::tbb-2 3'UTR] otIs252[lsy-6::YFP (fosmid); rol-6(su1006)] II; tbx-37(luc41[GFP::flex::tbx-37]) tbx-38(tm581) III	This study	MLC1219
Caenorhabditis elegans: otIs252[lsy-6::YFP (fosmid); rol-6(su1006)] II; tbx-37(tm314) tbx-38(tm581) III/qC1[dpy-19(e1259) glp-1(q339) qIs26[lag-2::GFP; rol-6(su1006)]	This study	MLC954
Caenorhabditis elegans: lucSi102[hsp-16.41p::zif-1::SL2::mCherry::his-11::tbb-2 3'UTR] II; wgIs37[pha-4::TY1::EGFP::3xFLAG; unc-119]	This study	MLC1467
Caenorhabditis elegans: lucSi100[hsp-16.41p::vhhGFP4::zif-1::SL2::mCherry::his-11::tbb-2 3'UTR] II; wgIs37[pha-4::TY1::EGFP::3xFLAG; unc-119]	This study	MLC1466
Caenorhabditis elegans: lucIs39[tbx-37p::mNeonGreen::2xNLS::tbx-37 3'UTR; pal-1p::mScarlet-I::2xNLS::tbb-2 3'UTR; med-2p::mScarlet-I::2xNLS::tbb-2 3'UTR]	This study	MLC1480
Caenorhabditis elegans: lucIs39[tbx-37p::mNeonGreen::2xNLS::tbx-37 3'UTR; pal-1p::mScarlet-I::2xNLS::tbb-2 3'UTR; med-2p::mScarlet-I::2xNLS::tbb-2 3'UTR)]; lsy-6(luc160[lsy-6::d150]) V	This study	MLC2309
Caenorhabditis elegans: otIs232[che-1p::mChopti; rol-6] II; lsy-6(luc160[lsy-6::d150]) V	This study	MLC2310

Caenorhabditis elegans: otIs252[lsy-6::YFP (fosmid); rol-6(su1006)] II	Caenorhabditis Genetics Center	WB Strain: OH8993
Caenorhabditis elegans: lucIs41[lsy-6::GFP::Δ150::5xUAS (fosmid); ttx-3p::mCherry]; lucEx959[tbx-37p::GAL4SK::tbx-37 3'UTR; elt-2p::mCherry]	This study	MLC1622
Caenorhabditis elegans: lucIs41[lsy-6::GFP::Δ150::5xUAS (fosmid); ttx-3p::mCherry]; lucEx960[tbx-37p::GAL4SK::tbx-37 3'UTR; elt-2p::mCherry]	This study	MLC1623
Caenorhabditis elegans: lucIs41[lsy-6::GFP::Δ150::5xUAS (fosmid); ttx-3p::mCherry]; lucEx961[tbx-37p::GAL4SK::tbx-37 3'UTR; elt-2p::mCherry]	This study	MLC1624
Caenorhabditis elegans: lucIs41[lsy-6::GFP::Δ150::5xUAS (fosmid); ttx-3p::mCherry]; lucEx962[tbx-37p::GAL4SK::tbx-37 3'UTR; elt-2p::mCherry]	This study	MLC1625
Caenorhabditis elegans: lucIs41[lsy-6::GFP::Δ150::5xUAS (fosmid); ttx-3p::mCherry]; lucEx937[tbx-37p::GAL4SK::VP64::tbx-37 3'UTR; elt-2p::mCherry]	This study	MLC1588
Caenorhabditis elegans: lucIs41[lsy-6::GFP::Δ150::5xUAS (fosmid); ttx-3p::mCherry]; lucEx938[tbx-37p::GAL4SK::VP64::tbx-37 3'UTR; elt-2p::mCherry]	This study	MLC1589
Caenorhabditis elegans: lucIs41[lsy-6::GFP::Δ150::5xUAS (fosmid); ttx-3p::mCherry]; lucEx939[tbx-37p::GAL4SK::VP64::tbx-37 3'UTR; elt-2p::mCherry]	This study	MLC1590
Caenorhabditis elegans: lucIs41[lsy-6::GFP::Δ150::5xUAS (fosmid); ttx-3p::mCherry]; lucEx940[tbx-37p::GAL4SK::VP64::tbx-37 3'UTR; elt-2p::mCherry]	This study	MLC1591
Caenorhabditis elegans: che-1(luc174) I; lucIs41[lsy-6p::GFP::d150::5xUAS; ttx-3p::mCherry] V; lucEx937[tbx-37p::GAL4SK::VP64; elt-2p::dsRed]	This study	MLC2320
Caenorhabditis elegans: che-1(luc174) I; lucIs41[lsy-6p::GFP::d150::5xUAS; ttx-3p::mCherry] V; lucEx938[tbx-37p::GAL4SK::VP64; elt-2p::dsRed]	This study	MLC2321
Caenorhabditis elegans: lucIs54[lsy-6::GFP::5xUAS (fosmid); ttx-3p::mCherry]; lucEx959[tbx-37p::GAL4SK::tbx-37 3'UTR; elt-2p::mCherry]	This study	MLC1920
Caenorhabditis elegans: lucIs54[lsy-6::GFP::5xUAS (fosmid); ttx-3p::mCherry]; lucEx1024[tbx-37p::GAL4SK::UNC-37::tbx-37 3'UTR; elt-2p::mCherry]	This study	MLC1741
Caenorhabditis elegans: lucIs54[lsy-6::GFP::5xUAS (fosmid); ttx-3p::mCherry]; lucEx1025[tbx-37p::GAL4SK::UNC-37::tbx-37 3'UTR; elt-2p::mCherry]	This study	MLC1742

Caenorhabditis elegans: lucIs41[lsy-6::GFP::Δ150::5xUAS (fosmid); ttx-3p::mCherry]	This study	MLC1557
Caenorhabditis elegans: lucIs54[lsy-6::GFP::5xUAS (fosmid); ttx-3p::mCherry]	This study	MLC1703
Caenorhabditis elegans: otIs386[lsy-6::GFP::Δ150 (fosmid); ttx-3p::mCherry]	Caenorhabditis Genetics Center	WB Strain: OH11115
Caenorhabditis elegans: unc-119(ed3) III; *wgIs159[tbx-2::TY1::eGFP::3xFLAG; unc-119]*	Caenorhabditis Genetics Center	WB Strain: OP159
Caenorhabditis elegans: unc-119(ed3) III; *wgIs311[tbx-7::TY1::eGFP::3xFLAG; unc-119]*	Caenorhabditis Genetics Center	WB Strain: OP311
Caenorhabditis elegans: lucEx552[tbx-8::GFP (fosmid); myo-2p::mCherry]	This study	MLC898
Caenorhabditis elegans: lucEx553[tbx-8::GFP (fosmid); myo-2p::mCherry]	This study	MLC899
Caenorhabditis elegans: unc-119(tm4063) III; *wgIs636[tbx-9::TY1::eGFP::3xFLAG; unc-119]*	Caenorhabditis Genetics Center	WB Strain: OP636
Caenorhabditis elegans: unc-119(tm4063) III; *wgIs368[tbx-11::TY1::eGFP::3xFLAG; unc-119]*	Caenorhabditis Genetics Center	WB Strain: OP368
Caenorhabditis elegans: lucEx822[mab-9p::MAB-9::T2A::GFP::H2B::mab-9 3'UTR; ttx-3p::mCherry]	This study	MLC1387
Caenorhabditis elegans: lucEx823[mab-9p::MAB-9::T2A::GFP::H2B::mab-9 3'UTR; ttx-3p::mCherry]	This study	MLC1388
Caenorhabditis elegans: lucEx824[mab-9p::MAB-9::T2A::GFP::H2B::mab-9 3'UTR; ttx-3p::mCherry]	This study	MLC1389
Caenorhabditis elegans: unc-119(ed3) III; *wgIs129[sea-1::TY1::eGFP::3xFLAG; unc-119]*	Caenorhabditis Genetics Center	WB Strain: OP129
Caenorhabditis elegans: lucEx818[tbx-31::GFP (fosmid); ttx-3p:mCherry]	This study	MLC1378
Caenorhabditis elegans: lucEx755[tbx-32::GFP (fosmid); ttx-3p::mCherry]	This study	MLC1276
Caenorhabditis elegans: lucEx628[tbx-33::GFP (fosmid); myo-2p:mCherry]	This study	MLC1019
Caenorhabditis elegans: lucEx757[tbx-33::GFP (fosmid); ttx-3p::mCherry]	This study	MLC1278
Caenorhabditis elegans: lucEx758[tbx-33::GFP (fosmid); ttx-3p::mCherry]	This study	MLC1279
Caenorhabditis elegans: lucEx825[tbx-34p::TBX-34::T2A::GFP::H2B::tbx-34 3'UTR; ttx-3p::mCherry]	This study	MLC1390
Caenorhabditis elegans: lucEx853[tbx-34p::TBX-34::T2A::GFP::H2B::tbx-34 3'UTR; ttx-3p::mCherry]	This study	MLC1435
Caenorhabditis elegans: lucEx756[tbx-35::GFP (fosmid); ttx-3p-mCherry]	This study	MLC1277
Caenorhabditis elegans: lucEx826[tbx-36p::TBX-36::T2A::GFP::H2B::tbx-36 3'UTR; ttx-3p::mCherry]	This study	MLC1391
Caenorhabditis elegans: lucEx827[tbx-36p::TBX-36::T2A::GFP::H2B::tbx-36 3'UTR; ttx-3p::mCherry]	This study	MLC1392
Caenorhabditis elegans: lucEx935[tbx-39p::TBX-39::T2A::GFP::H2B::tbx-39 3'UTR; ttx-3p::mCherry]	This study	MLC1578
Caenorhabditis elegans: lucEx936[tbx-39p::TBX-39::T2A::GFP::H2B::tbx-39 3'UTR; ttx-3p::mCherry]	This study	MLC1579

Caenorhabditis elegans: lucEx883[tbx-43p::TBX-43::T2A::GFP::H2B::tbx-43 3'UTR; ttx-3p::mCherry]	This study	MLC1493
Caenorhabditis elegans: lucEx893[tbx-43p::TBX-43::T2A::GFP::H2B::tbx-43 3'UTR; ttx-3p::mCherry]	This study	MLC1506
Caenorhabditis elegans: lsy-6(ot150) V; *nre-1(hd20) lin-15b(hd126) X*; *otIs186[gcy-5p::GFP; rol-6(su1006)]*	Hobert Lab Columbia University	OH8996
Caenorhabditis elegans: otIs252[lsy-6::YFP (fosmid); rol-6(su1006)] II; *tbx-11(luc144) III*	This study	MLC1977
Caenorhabditis elegans: otIs252[lsy-6::YFP (fosmid); rol-6(su1006)] II; *tbx-33(gk3098) /hT2[qIs48[myo-2::GFP; pes-10::GFP; F22B7.9::GFP] III*	This study	MLC1928
Caenorhabditis elegans: otIs252[lsy-6::YFP (fosmid); rol-6(su1006)] II; *tbx-43(luc131) III*; *otIs220[gcy-5p::mCherry; rol-6(su1006)] IV*	This study	MLC1866
Caenorhabditis elegans: otIs252[lsy-6::YFP (fosmid); rol-6(su1006)] II; *otIs235[che-1p::mChopti; rol-6(su1006)] V*	This study	MLC2239
Caenorhabditis elegans: otIs252[lsy-6::YFP; rol-6(su1006)] II; tbx-37(tm314) tbx-38(tm581)/qC1[dpy-19(e1259) glp-1(q339) qIs26] III; lucEx1191[che-1p::mChopti; unc-122p::RFP]	This study	MLC2180
Caenorhabditis elegans: tbx-37(tm314) tbx-38(tm581)/qC1[dpy-19(e1259) glp-1(q339) qIs26] III; *lucEx1179[lsy-6p::GFP::Δ150::5xUAS (fosmid); che-1p::mChopti; tbx-37p::GAL4SK::VP64; unc-122p::RFP]*	This study	MLC2110
Caenorhabditis elegans: tbx-37(tm314) tbx-38(tm581)/qC1[dpy-19(e1259) glp-1(q339) qIs26] III, *lucEx1180[lsy-6p::GFP::Δ150::5xUAS (fosmid); che-1p::mChopti; tbx-37p::GAL4SK::VP64; unc-122p::RFP]*	This study	MLC2111
Caenorhabditis elegans: tbx-37(tm314) tbx-38(tm581)/qC1[dpy-19(e1259) glp-1(q339) qIs26] III, *lucEx1181[lsy-6p::GFP::Δ150::5xUAS (fosmid); che-1p::mChopti; tbx-37p::GAL4SK::VP64; unc-122p::RFP]*	This study	MLC2112
Caenorhabditis elegans: otIs283[lsy-6::YFP (fosmid); rol-6(su1006)] V; lsy-2(ot64)/+ X	Hobert Lab Columbia University	OH9593
Caenorhabditis elegans: otEx4375[lsy-6::YFP (fosmid); ttx-3p::mCherry]	Hobert Lab Columbia University	OH9863
Caenorhabditis elegans: otEx4376[lsy-6::YFP (fosmid); ttx-3p::mCherry]	Hobert Lab Columbia University	OH9864
Caenorhabditis elegans: otEx4379[lsy-6::YFP::ΔEbox (fosmid); ttx-3p::mCherry]	Hobert Lab Columbia University	OH9867
Caenorhabditis elegans: otEx4380[lsy-6::YFP::ΔEbox (fosmid); ttx-3p::mCherry]	Hobert Lab Columbia University	OH9868
Caenorhabditis elegans: otEx4381[lsy-6::YFP::ΔEbox (fosmid); ttx-3p::mCherry]	Hobert Lab Columbia University	OH9869

Caenorhabditis elegans: otIs235[che-1p::mChopti; rol-6(su1006)] V; lucEx881[C32C4.16::T2A::GFP::H2B; unc-122p::RFP]	This study	MLC2240
Caenorhabditis elegans: lucEx1129[C32C4.16::T2A::GFP::H2B (fosmid); opt-3p::tagRFP; unc-122p::RFP]	This study	MLC1976
Caenorhabditis elegans: lucEx1130[C32C4.16::T2A::GFP::H2B (fosmid); flp-8p::mCherry; unc-122p::RFP]	This study	MLC1978
Caenorhabditis elegans: lucEx881[C32C4.16::T2A::GFP::H2B (fosmid); unc-122p::RFP]	This study	MLC1491
Caenorhabditis elegans: tbx-37(tm314) tbx-38(tm581)/qC1[dpy-19(e1259) glp-1(q339) qIs26[lag-2::GFP; rol-6(su1006)]] III, lucEx881 [C32C4.16::T2A::GFP::H2B (fosmid); unc-122p::RFP]	This study	MLC1961
Caenorhabditis elegans: otIs235[che-1p::mChopti; rol-6(su1006)] V; lucEx1176[lsy-6::Δ150::C32C4.16::T2A::GFP::H2B (fosmid); unc-122p::RFP]	This study	MLC2104
Caenorhabditis elegans: otIs670[NeuroPal] V, lucEx881[C32C4.16::T2A::GFP::H2B (fosmid); unc-122p::RFP]	This study	MLC1962

Table S5. Recombinant DNA used in this study (related to STAR Methods and Key Resources Table)

Recombinant DNA		
Fosmid:<br>pCC1 - *tbx-8::GFP* (WRM065aG10)	This study	N/A
Fosmid:<br>pCC1 - *tbx-31::GFP* (WRM067dD02)	This study	N/A
Fosmid:<br>pCC1 - *tbx-32::GFP* (WRM0641aB04)	This study	N/A
Fosmid:<br>pCC1 - *tbx-33::GFP* (WRM0619bA09)	This study	N/A
Fosmid:<br>pCC1 - *tbx-35::GFP* (WRM0619dE06)	This study	N/A
Fosmid:<br>pCC1 - *lsy-6::GFP::Δ150::5xUAS* (WRM0628bA07)	This study	N/A
Fosmid:<br>pCC1 - *lsy-6::GFP::5xUAS* (WRM0628bA07)	This study	N/A
Fosmid:<br>pCC1 - *C32C4.16::T2A::GFP::H2B* (WRM0628bA07)	This study	N/A
Fosmid:<br>pCC1 - *lsy-6::Δ150::C32C4.16::T2A::GFP::H2B* (WRM0628bA07)	This study	N/A
Plasmid:<br>puc19 - *tbx-37::GFP::FLEX*	This study	N/A
Plasmid:<br>puc19 - *tbx-38::GFP::FLEX*	This study	N/A
Plasmid:<br>pCFJ350 – *hsp-16.41p::ZIF-1::SL2::mCherry::his-11::tbb-2 3'UTR*	This study	N/A
Plasmid:<br>pCFJ350 – *hsp-16.41p::vhhGFP4::ZIF-1::SL2::mCherry::his-11::tbb-2 3'UTR*	This study	N/A
Plasmid:<br>psC-B-AmpKan - *tbx-37p::mNeonGreen::2xNLS::tbx-37 3'UTR*	This study	N/A
Plasmid:<br>puc19 - *pal-1p::mScarlet-I::2xNLS::tbb-2 3'UTR*	This study	N/A
Plasmid:<br>puc19 - *med-2p::mScarlet-I::2xNLS::tbb-2 3'UTR*	This study	N/A
Plasmid:<br>psC-B-AmpKan – *tbx-34p::tbx-34::T2A::GFP::H2B::tbx-34 3'UTR*	This study	N/A
Plasmid:<br>psC-B-AmpKan – *tbx-36p::tbx-36::T2A::GFP::H2B::tbx-36 3'UTR*	This study	N/A
Plasmid:<br>psC-B-AmpKan – *tbx-39p::tbx-39::T2A::GFP::H2B::tbx-39 3'UTR*	This study	N/A

Plasmid:<br>psC-B-AmpKan – *tbx-43p::tbx-43::T2A::GFP::H2B::tbx-43 3'UTR*	This study	N/A
Plasmid:<br>psC-B-AmpKan – *tbx-37p::GAL4SK::tbx-37 3'UTR*	This study	N/A
Plasmid:<br>psC-B-AmpKan – *tbx-37p::GAL4SK::VP64::tbx-37 3'UTR*	This study	N/A
Plasmid:<br>psC-B-AmpKan – *tbx-37p::GAL4SK::UNC-37::tbx-37 3'UTR*	This study	N/A
Plasmid:<br>pSM – *myo-2p::mCherry*	Zimmer lab<br>University of Vienna	N/A
Plasmid:<br>pPD – *ttx-3p::mCherry*	Hobert Lab<br>Columbia University	N/A
Plasmid:<br>pPD – *elt-2p::dsRED*	Hobert Lab<br>Columbia University	N/A
Plasmid:<br>pPD – *unc-122p::RFP*	Hobert Lab<br>Columbia University	N/A
Plasmid:<br>pSM – *flp-8p::mCherry*	Zimmer lab<br>University of Vienna	N/A
Plasmid:<br>pSM – *opt-3p::tagRFP*	Zimmer lab<br>University of Vienna	N/A
Plasmid:<br>pPD – *che-1p::mChopti*	Hobert Lab<br>Columbia University	N/A
Plasmid:<br>pL440 control RNAi	Kamath & Ahringer, 2003	N/A
Plasmid:<br>pL440 – *mab-9* RNAi	Kamath & Ahringer, 2003	*T27A1.6*
Plasmid:<br>pL440 – *mls-1* RNAi	Kamath & Ahringer, 2003	*H14A12.4*
Plasmid:<br>pL440 – *sea-1* RNAi	Kamath & Ahringer, 2003	*F19B10.9*
Plasmid:<br>pL440 – *tbx-2* RNAi	Kamath & Ahringer, 2003	*F21H11.3*
Plasmid:<br>pL440 – *tbx-7* RNAi	Kamath & Ahringer, 2003	*ZK328.8*
Plasmid:<br>pL440 – *tbx-8* RNAi	Kamath & Ahringer, 2003	*T07C4.2*
Plasmid:<br>pL440 – *tbx-9* RNAi	Kamath & Ahringer, 2003	*T07C4.6*
Plasmid:<br>pL440 – *tbx-11* RNAi	Kamath & Ahringer, 2003	*F40H6.4*
Plasmid:<br>pL440 – *tbx-30* RNAi	This study	N/A
Plasmid:<br>pL440 – *tbx-31* RNAi	Kamath & Ahringer, 2003	*C36C9.2*
Plasmid:<br>pL440 – *tbx-32* RNAi	This study	N/A
Plasmid:<br>pL440 – *tbx-33* RNAi	Kamath & Ahringer, 2003	*Y66A7A.8*

Plasmid:<br>pL440 – *tbx-34* RNAi	This study	N/A
Plasmid:<br>pL440 – *tbx-35* RNAi	This study	N/A
Plasmid:<br>pL440 – *tbx-36* RNAi	Kamath & Ahringer, 2003	*ZK829.5*
Plasmid:<br>pL440 – *tbx-37* RNAi	This study	N/A
Plasmid:<br>pL440 – *tbx-38* RNAi	Kamath & Ahringer, 2003	*C24H11.3*
Plasmid:<br>pL440 – *tbx-39* RNAi	This study	N/A
Plasmid:<br>pL440 – *tbx-40* RNAi	This study	N/A
Plasmid:<br>pL440 – *tbx-41* RNAi	This study	N/A
Plasmid:<br>pL440 – *tbx-42* RNAi	Kamath & Ahringer, 2003	*Y59E9AR.5*
Plasmid:<br>pL440 – *tbx-43* RNAi	This study	N/A

Supplementary Figures

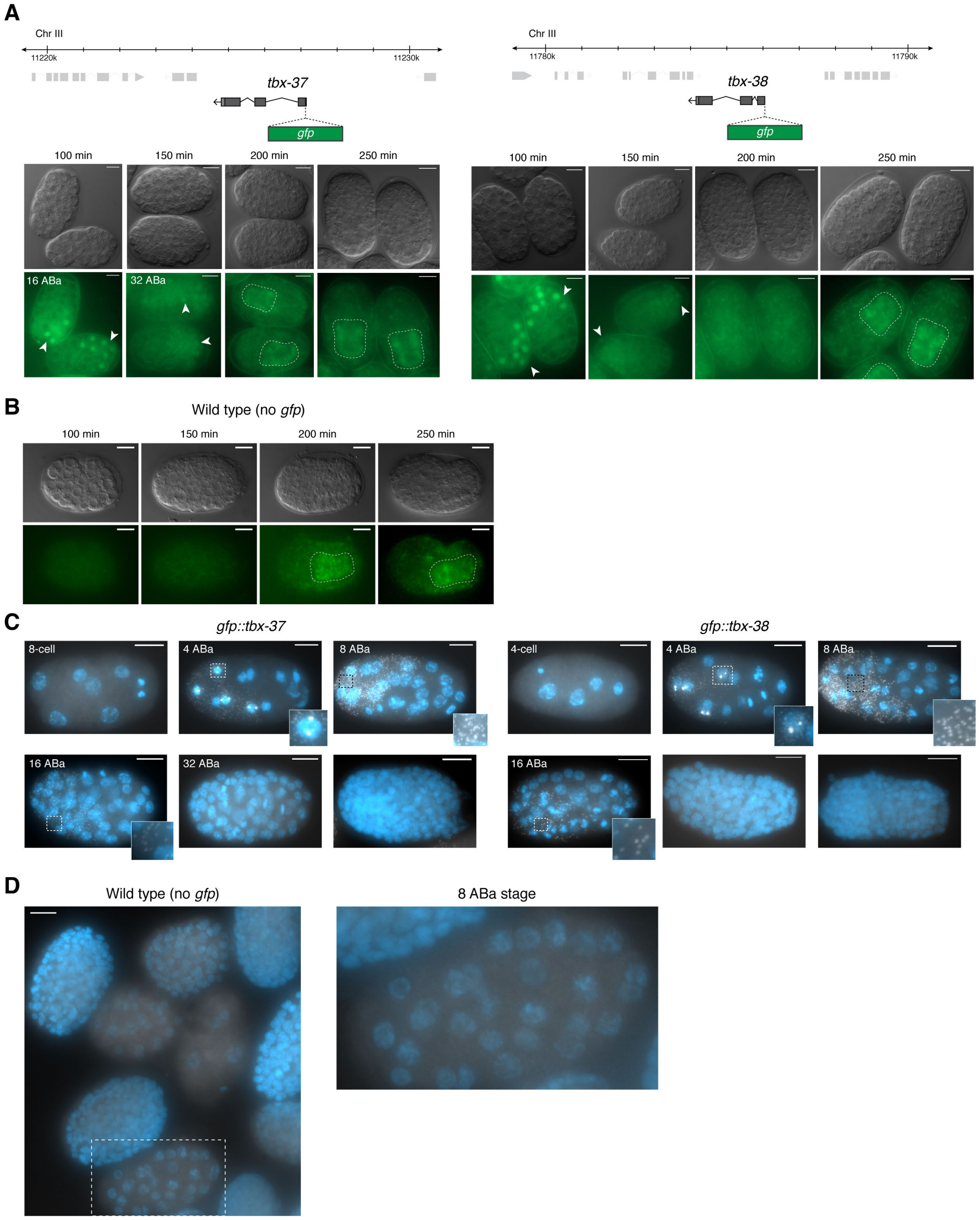

Figure S1. TBX-37/38 are transiently expressed (related to Figures 2 and 3)
A. Schematic of the CRISPR engineered *tbx-37* and *tbx-38* loci and GFP expression from embryos carrying these alleles. DIC (single plane) and GFP fluorescence (max. intensity projections) are shown. Embryos were collected at the 2-cell stage and allowed to develop for the indicated times at 20ºC. Arrowheads point to nuclei with visible GFP signal. At later timepoints the developing gut displays autofluorescence and is delimited by the dashed line. All scale bars represent 10 µm. **B.** Representative images of wt, non-transgenic embryos at the same timepoints as in A to show autofluorescence levels (max. intensity projections). **C.** Analysis of transcription from the *gfp::tbx-37* and *gfp::tbx-38* loci by smFISH with probes against *gfp.* The smFISH signal is shown in white and DAPI staining in blue (max. intensity projections), both signals are overlayed. Embryos were staged by counting DAPI-stained nuclei. Insets show the onset of transcription at the 4 ABa stage, then mRNA in the cytoplasm peaking at the 8 ABa stage and then rapidly disappearing. All scale bars represent 10 µm. **D.** Negative control for smFISH staining, on wild-type, non-transgenic embryos

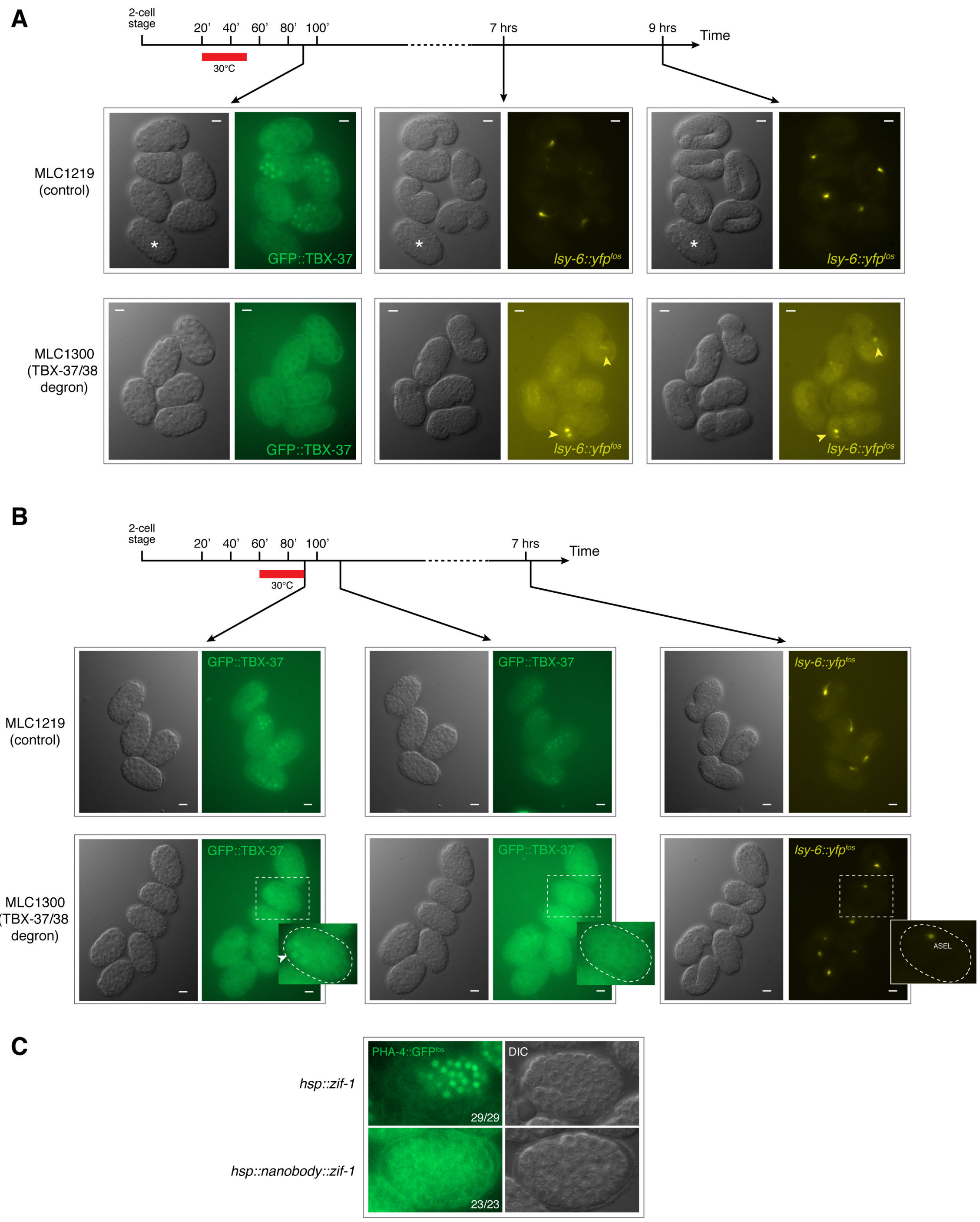

Figure S2. Experimental design and representative images for the TBX-37 degron experiment (related to Figure 3)

A. Timescale of the forced degradation experiment in Figure 3. Activation of the degron at the first time point (t1 = 20-50 minutes) resulted in robust degradation of GFP::TBX-37 (in a *tbx-38(0)* background), at its peak of expression (compare GFP::TBX-37 signal in control vs. degron at 90 min). This degradation prevented *lsy-6::yfp* activation (compare YFP signal in control vs. degron at 7 and 9 hours) and phenocopied the *tbx-37/38(0)* mutant morphology (compare DIC images). The arrowheads point to ectopic expression of *lsy-6::yfp* in a pair of cells in the tail of the embryo that we sometimes observe after heat shock treatment. The asterisk marks an embryo that arrested and died early in the experiment. **B.** Activation of the degron at the second time point (t2 = 60-90 minutes) already caused lower GFP::TBX-37 signal at the 90 min timepoint but we observed loss of signal 30 min later (120 min timepoint). This robust degradation of GFP::TBX-37 (in a *tbx-38(0)* background) slightly after their peak of expression did not affect *lsy-6::yfp* activation nor did it cause morphological defects as in the *tbx-37/38(0)* mutant. **C.** Activation of the degron at the second timepoint (t2 = 60-90 min) is able to clear PHA-4::GFP, indicating that the system is able to efficiently degrade a highly-expressed nuclear protein at this time point. The observed green signal in the degron strain is autofluorescence, different from the nuclear localized PHA-4::GFP signal.

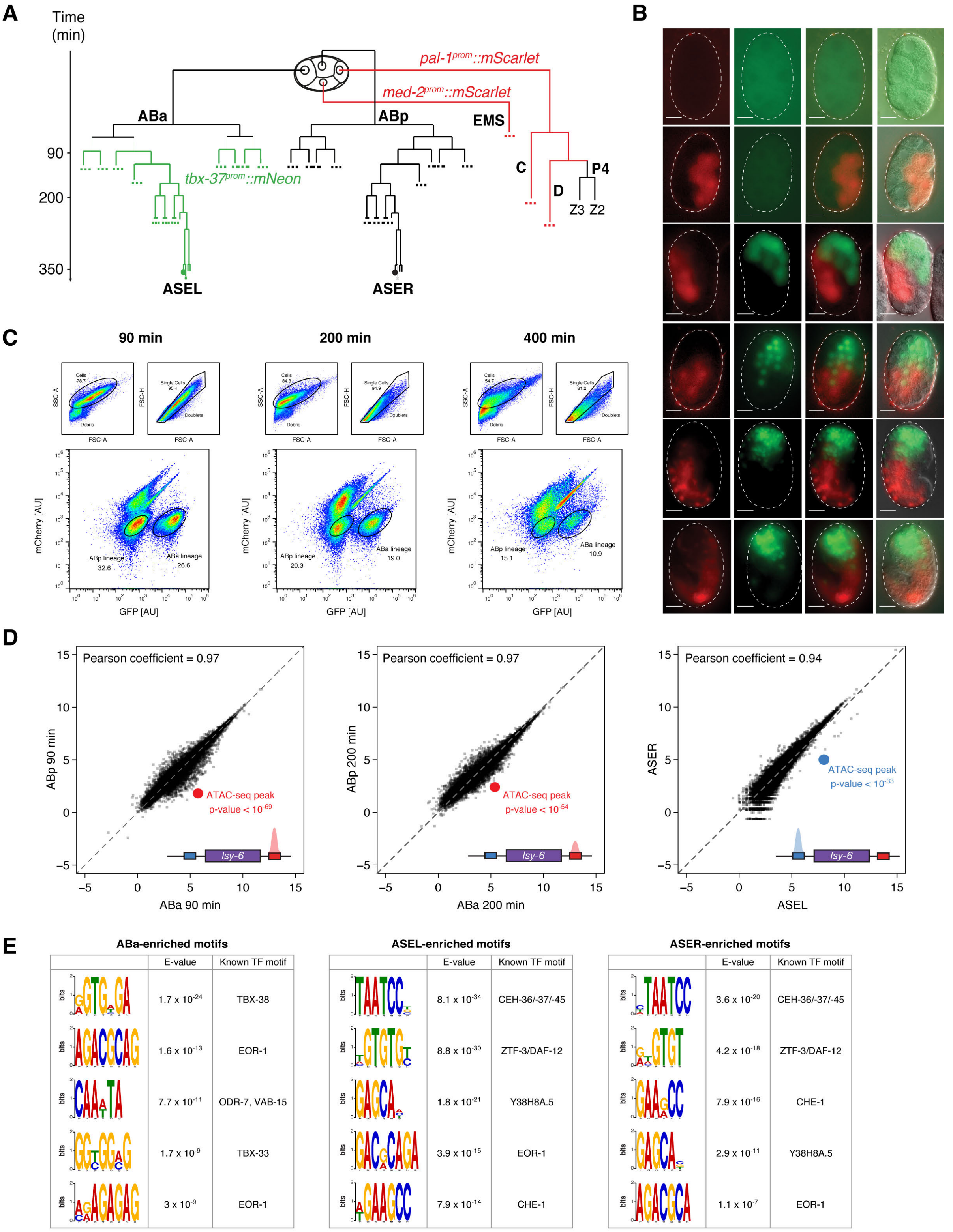

Figure S3. Experimental design for lineage specific FACS (related to Figure 4)

A. Schematic of the embryo labeling strategy that enables separation of ABa and ABp derived cells by FACS. Descendants from the ABa blastomere are labeled with a *tbx-37^{prom}::mNeonGreen* and cells from EMS and C/D lineages are labeled with *med-2^{prom}::mScarlet* and *pal-1^{prom}::mScarlet*, respectively. The Z2 and Z3 cells also remain unlabeled, but they represent a very minor fraction compared to the rest of the ABp lineage. **B.** Representative images following the development of a labeled embryo over time. DIC and fluorescence images are shown. From top to bottom, 6-cell, 8-10-cell, 8 ABa, 16 ABa, comma and 1.5-fold stages. Scale bars = 10 μm.

C. FACS plot representation of the ABa (GFP positive) and ABp (GFP and mScarlet negative) sorted cell population for the three ATAC-seq time points. FACS pseudo-colored plots for cells dissociated from the described strain at different timepoints. From left to right, 90 min, 200 min and 400 min. The top panels show the gating used to separate single cells from debris and doublets or higher-order cell aggregates. The bottom panel shows the separation of GFP positive, mScarlet negative cells corresponding to the ABa lineage; and double negative cells corresponding to ABp descendants. The cell populations outlined in black were isolated by FACS for the preparation of ATAC-seq libraries. **D.** Pairwise comparisons of ATAC-seq signal, in $\log_2$ (cpm), for all peaks called by MACS in at least one ATAC-seq sample. Pearson's correlation coefficients are shown. The p-values (MACS2) for the called *lsy-6* peaks in ABa and ASEL are shown. **E.** De novo motif discovery using the MEME Suite was used to find enriched motifs (and predicted TFs that can bind them) in ABa (using peaks detected in ABa at 90 min that do not overlap with peaks in ABp at 90 min), ASEL (using peaks detected in ASEL that do not overlap with peaks in ABa at 350 min) and ASER (using peaks detected in ASER that do not overlap with peaks in ABp at 350 min). ABa-specific accessible regions have significant enrichment of TBX-37/38 binding sites, while both ASEL and ASER accessible regions have significant enrichment of motifs that correspond to CHE-1 binding consensus and other TFs.

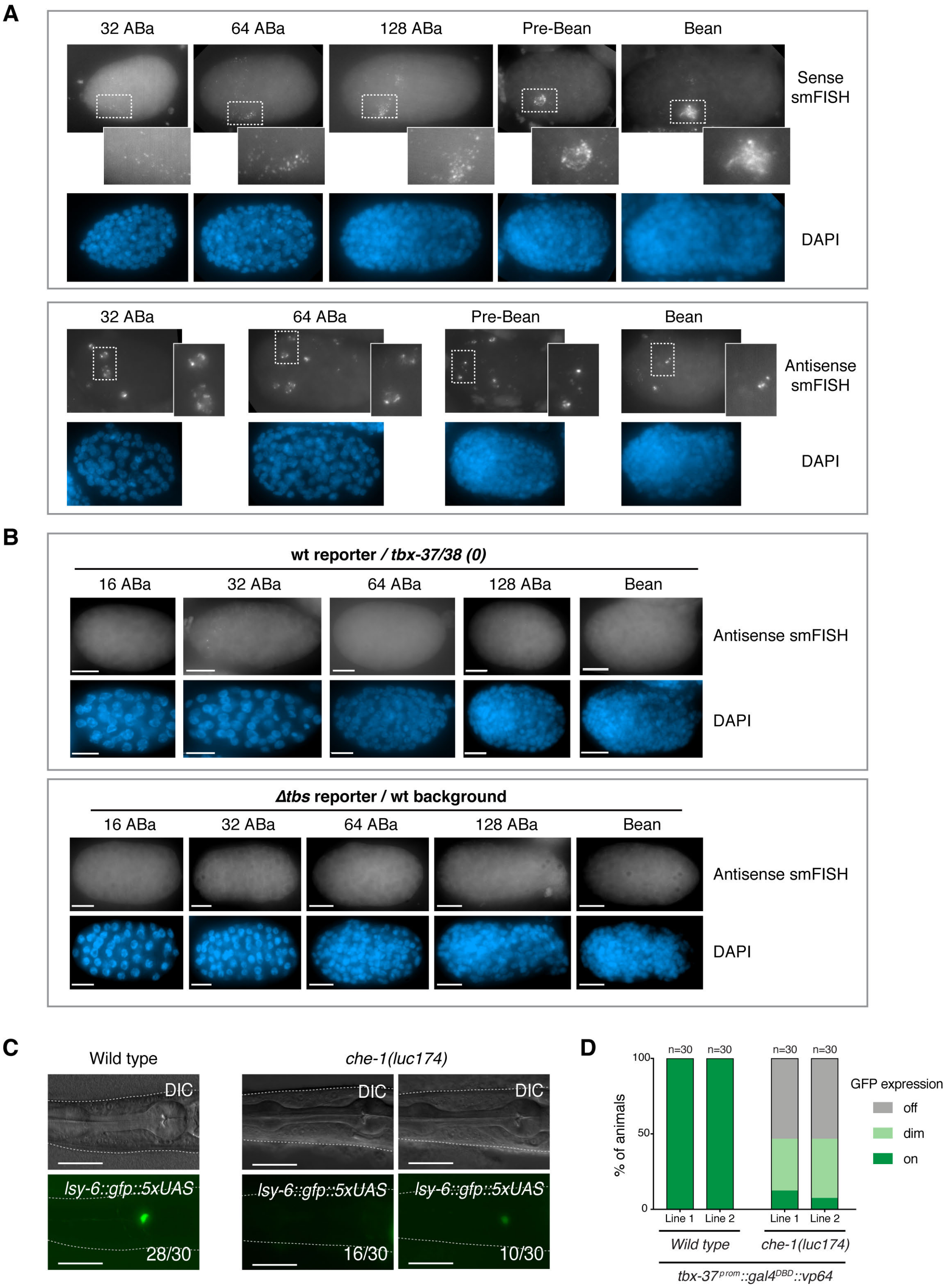

Figure S4. Transcription of the *lsy-6* locus occurs bidirectionally and requires TBX-37/38 binding (related to Figure 5)

A. Single molecule in situ hybridization on embryos carrying the *lsy-6::yfp*^{fosmid} with probes against the sense *yfp* transcript show low levels of sense transcription of the *lsy-6* locus from 32 AB stage until boosting of expression by CHE-1 in the ASEL mother cell (Pre-bean). Embryos were staged by counting DAPI-stained nuclei. Dashed boxes indicate zoomed regions showing bright nuclear foci. (Bottom) Same as above but using a probe set against the antisense of *yfp.* The insets show the presence of bright nuclear foci, indicative of continuous transcription. **B.** Antisense transcription of *lsy-6::yfp* is fully dependent on TBX-37/38 (top) and its binding sites (bottom). **C.** (Left) Representative image of animals like the ones scored in Fig. 5B, in which tethering of GAL4-VP64 restores *lsy-6::gfp::Δtbs::5xUAS* expression in ASEL. (Right) Representative images of the same tethering experiment in a *che-1*-deficient background. **D.** Scoring for the experiment described in C. Two independent lines were scored.

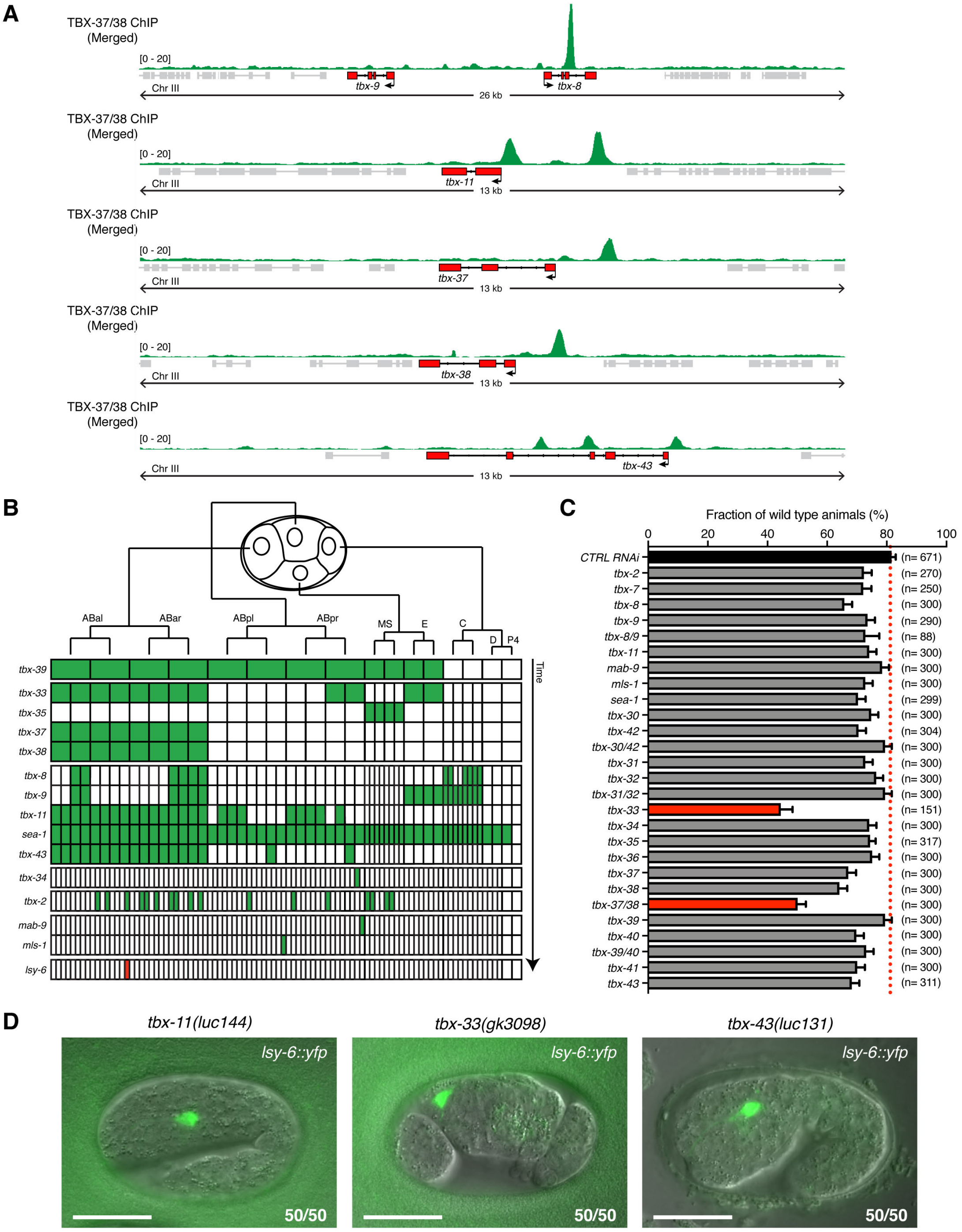

Figure S5. TBX-37/38 action at the *lsy-6* locus is not relayed by other T-Box transcription factors (related to Figure 6)

A. Aggregated GFP-TBX-37/38 ChIP-seq signal over T-box gene loci. TBX-37/38 were found to bind in the vicinity of *tbx-8/9*, *tbx-11*, *tbx-37*, *tbx-38* and *tbx-43*. **B.** Reporters for most *tbx* genes were generated and examined for expression. Those that showed early embryonic expression were lineaged with the aid of 4D microscopy and Simi Biocell software. The onset of expression of those early-expressed *tbx* genes is represented as a green box in the matrix. Boxes get narrower with every cell division. Expression of *tbx-33*, *tbx-37*, *tbx-38*, *tbx-11*, *sea-1*, *tbx-43* and *tbx-2* was observed in the ABalpp lineage branch, which will give rise to ASEL. **C.** Effect of RNA interference against different *tbx* genes on *lsy-6* expression in a sensitized background. A point mutation in the CHE-1 binding site in the promoter of *lsy-6* (*ot150*) affects CHE-1 binding efficiency and results in a sensitized background in which 20% of animals fail to express enough *lsy-6* and to specify ASEL (Sarin et al., 2007). The failure to specify ASEL is monitored by its ectopic expression of the ASER terminal fate marker *gcy-5*^{prom}*::GFP*. Because some of these T-box factors occur in recently duplicated pairs, we also tested for possible redundancy by doing double RNAi. Only RNAi against *tbx-37/38* or against *tbx-33* caused further enhancement of the defect. The effect of *tbx-33* was further explored by using a deletion allele (panel D) but could not be validated. **D.** Expression of the *lsy-6::yfp* fosmid reporter is not affected by deletion of *tbx-11, tbx-33* or *tbx-43*. All scale bars represent 20 μm.

A

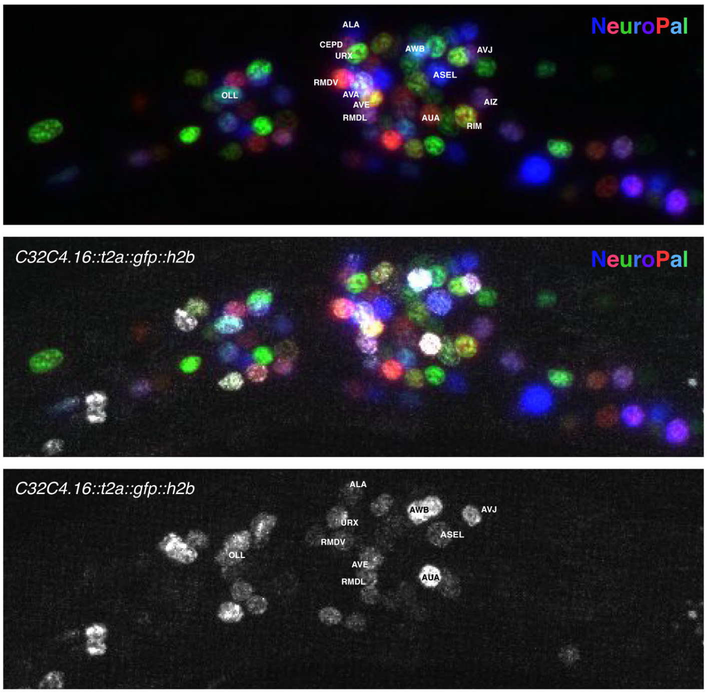

B

Worm	Orientation	Identified Neurons
1	Left	AUAL, AWBL, AVEL, RMDL, ASEL, AVJL/R, RMDV
2	Left	AVEL, RMDL, ASEL, OLLL
3	Left	AUAL, AWBL, AVEL, RMDL, ASEL, AVJL/R, RMDV, SMDVL/R, RMDD
4	Right	AUAL, AWBL, AVEL, RMDL, ASEL, AVJL/R, RMDVL/R, SMDVL/R, RMDDR, AVAR

C

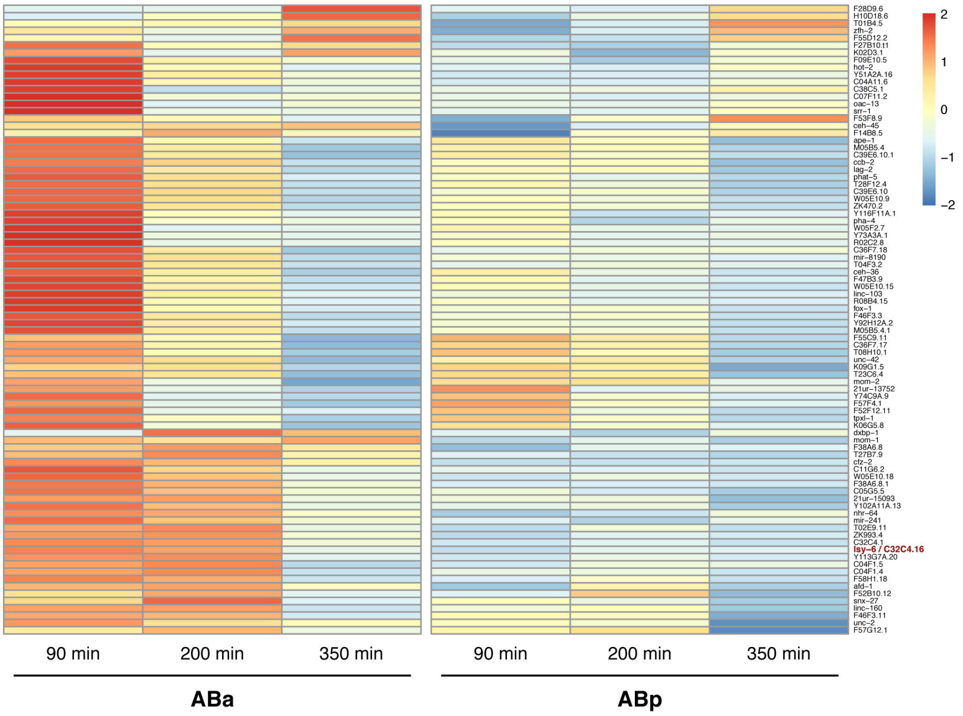

Figure S6. Identification of neurons expressing C32C4.16 and of candidate genes primed by TBX-37/38 (related to Figure 7)

A. Representative images of C32C4.16 reporter in the NeuroPal system, which uses a combination of over 40 neuron-specific promoters to allow easy neuronal identification by their unique color combination and position (Yemini et al., 2019). Shown are the neuronal identity assignments based on these properties (top), the nuclear GFP-H2B signal from the C32C4.16 reporter (bottom) and an overlay of both (middle). Max. intensity projections from one animal are shown. **B.** Four L1-stage larvae were analyzed in detail using this system. The neurons we could confidently identify are listed. In all four cases we could identify the right-side counterparts of AUAL, AWBL, AVEL, RMDL and ASEL, but we never saw GFP expression in those cells. **C.** Heatmap showing the scaled ATAC-seq accessibility of 86 genes that harbor TBX-37/38 binding sites (by ChIP-seq) and are asymmetrically accessible in ABa at 90 min, but not in ABp. The regulatory region between *lsy-6* and C32C4.16 are highlighted in red. These genes represent candidates for regulation similar to *lsy-6.*
