# Supplementary material for: Combinatorial Action of Temporally Segregated Transcription Factors
Source: Dev Cell. 2020 Nov 23;55(4):483–499.e7. doi: 10.1016/j.devcel.2020.09.002 (PMC7704111; doi:10.1016/j.devcel.2020.09.002)
Supplement: Document S2. Article plus Supplemental Information [file mmc2.pdf]

Developmental Cell

Article

Combinatorial Action of Temporally Segregated Transcription Factors

Graphical Abstract

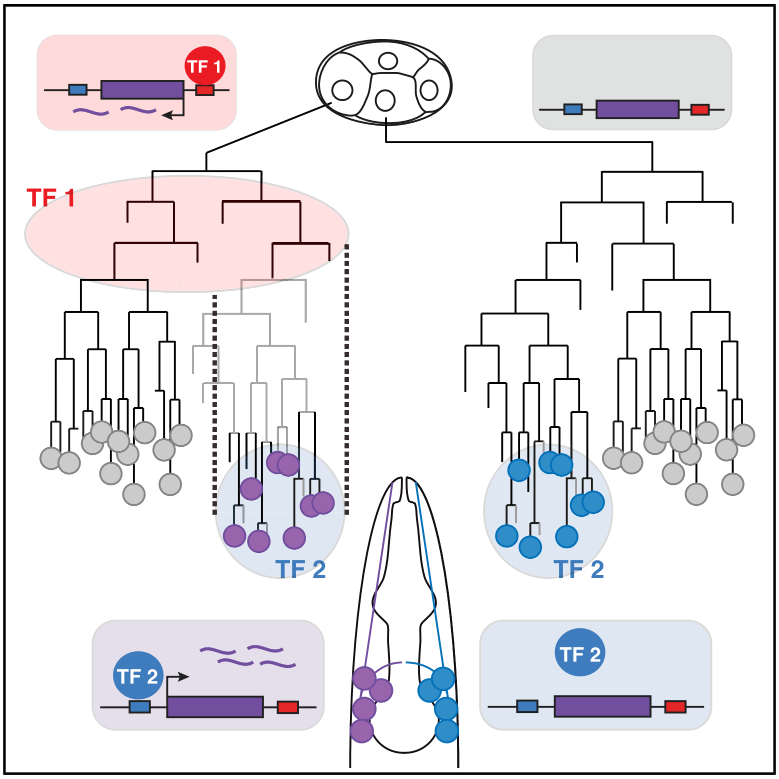

Highlights

- Lineage-specific priming enables asymmetric gene expression in L/R neuron pairs
- Transient, lineage-specific TFs prime a locus for later activation by a bilateral TF
- An early active transcriptional state is necessary and sufficient for priming
- Maintenance of asymmetric primed state occurs in a symmetric regulatory environment

Authors

Julien Charest, Thomas Daniele, Jingkui Wang, ..., Josef Röhsner, Paula Gutiérrez-Pérez, Luisa Cochella

Correspondence

cochella@imp.ac.at

In Brief

Studying neuronal specification in *C. elegans*, Charest et al. reveal that lineage-specific priming by a transiently expressed transcription factor allows activation of the primed gene by another transcription factor acting four cell divisions later. The findings suggest that the intersection of temporally segregated transcription factors contributes to cell-specific gene expression and cell diversification.

Charest et al., 2020, Developmental Cell *55*, 483–499
November 23, 2020 © 2020 The Authors. Published by Elsevier Inc.
https://doi.org/10.1016/j.devcel.2020.09.002

Developmental Cell

CellPress
OPEN ACCESS

Article

Combinatorial Action of Temporally Segregated Transcription Factors

Julien Charest,[1,2] Thomas Daniele,[1,2] Jingkui Wang,[1,3] Aleksandr Bykov,[1,3] Ariane Mandlbauer,[1,3] Mila Asparuhova,[1] Josef Röhsner,[1] Paula Gutiérrez-Pérez,[1] and Luisa Cochella[1,4,*]
[1]Research Institute of Molecular Pathology (IMP), Vienna BioCenter (VBC), Campus-Vienna-Biocenter 1, 1030 Vienna, Austria
[2]These authors contributed equally
[3]These authors contributed equally
[4]Lead Contact
*Correspondence: cochella@imp.ac.at
https://doi.org/10.1016/j.devcel.2020.09.002

SUMMARY

Combinatorial action of transcription factors (TFs) with partially overlapping expression is a widespread strategy to generate novel gene-expression patterns and, thus, cellular diversity. Known mechanisms underlying combinatorial activity require co-expression of TFs within the same cell. Here, we describe the mechanism by which two TFs that are never co-expressed generate a new, intersectional expression pattern in *C. elegans* embryos: lineage-specific priming of a gene by a transiently expressed TF generates a unique intersection with a second TF acting on the same gene four cell divisions later; the second TF is expressed in multiple cells but only activates transcription in those where priming occurred. Early induction of active transcription is necessary and sufficient to establish a competent state, maintained by broadly expressed regulators in the absence of the initial trigger. We uncover additional cells diversified through this mechanism. Our findings define a mechanism for combinatorial TF activity with important implications for generation of cell-type diversity.

INTRODUCTION

Transcription of developmentally regulated genes typically requires combinatorial activity of multiple transcription factors (TFs). Combinatorial use of TFs enables the generation of novel, specific gene-expression patterns that exceed the number of available TFs, through creation of intersectional gene-expression domains (Figure 1A, left) (Allan and Thor, 2015; Reiter et al., 2017; Spitz and Furlong, 2012). In fact, different cell types are largely determined by the differential expression of combinations of TFs that activate transcription of sets of effector genes, giving each cell type its unique structural and physiological properties (Hobert, 2008). The specification of individual cell identities by combinations of TFs enables integration of signaling events and intrinsic transcriptional programs; thus, combinatorial action of TFs is a basis for cellular diversification during development (Allan and Thor, 2015; Hobert, 2016).

Different molecular mechanisms for combinatorial activity of two or more TFs have been described (Lee et al., 2008; Long et al., 2016; Reiter et al., 2017; Wenick and Hobert, 2004; Zaret and Carroll, 2011; Zaret and Mango, 2016), most of which are based on TFs simultaneously present within the same cell. Consistently, known cases of combinations of TFs to generate cell-type diversity during development rely on spatial intersection of TFs with partially overlapping expression patterns (Figure 1A, left) (Alaynick et al., 2011; Wenick and Hobert, 2004; Xue et al., 1993; Zhang et al., 2014).

The *C. elegans* microRNA (miRNA) *lsy-6* is transcribed exclusively in one of two left/right morphologically symmetric neurons, the ASE sensory-neuron pair (Cochella and Hobert, 2012; Johnston and Hobert, 2003). Specific transcription of *lsy-6* in the left ASE neuron (ASEL) defines two alternative sensory-neuron fates with different molecular and functional properties (Hobert, 2014) that are necessary for animal behavior (Pierce-Shimomura et al., 2001; Suzuki et al., 2008) and represent the only currently known directed asymmetry in the worm nervous system. Intriguingly, asymmetric *lsy-6* transcription requires direct action of the ASE terminal selector TF CHE-1 on a well-characterized binding site, even though CHE-1 is present in both ASE neurons where it activates hundreds of symmetric genes (Etchberger et al., 2007, 2009; Leyva-Díaz and Hobert, 2019; Uchida et al., 2003). Thus, ASEL-specific expression of *lsy-6* cannot be determined solely by CHE-1: it depends instead on an intersectional strategy defined by the different lineage histories of the two ASE neurons, which diverge at the 4-cell stage of embryogenesis, eight cell divisions prior to the birth of the ASEs (Figure 1B) (Cochella and Hobert, 2012; Poole and Hobert, 2006; Sulston et al., 1983).

The two lineage branches that give rise to the ASE neurons are distinguished by exclusive expression of two redundant T-box TFs, TBX-37/38, in the ASEL precursor lineage, ABa

This is an open access article under the CC BY license (http://creativecommons.org/licenses/by/4.0/).

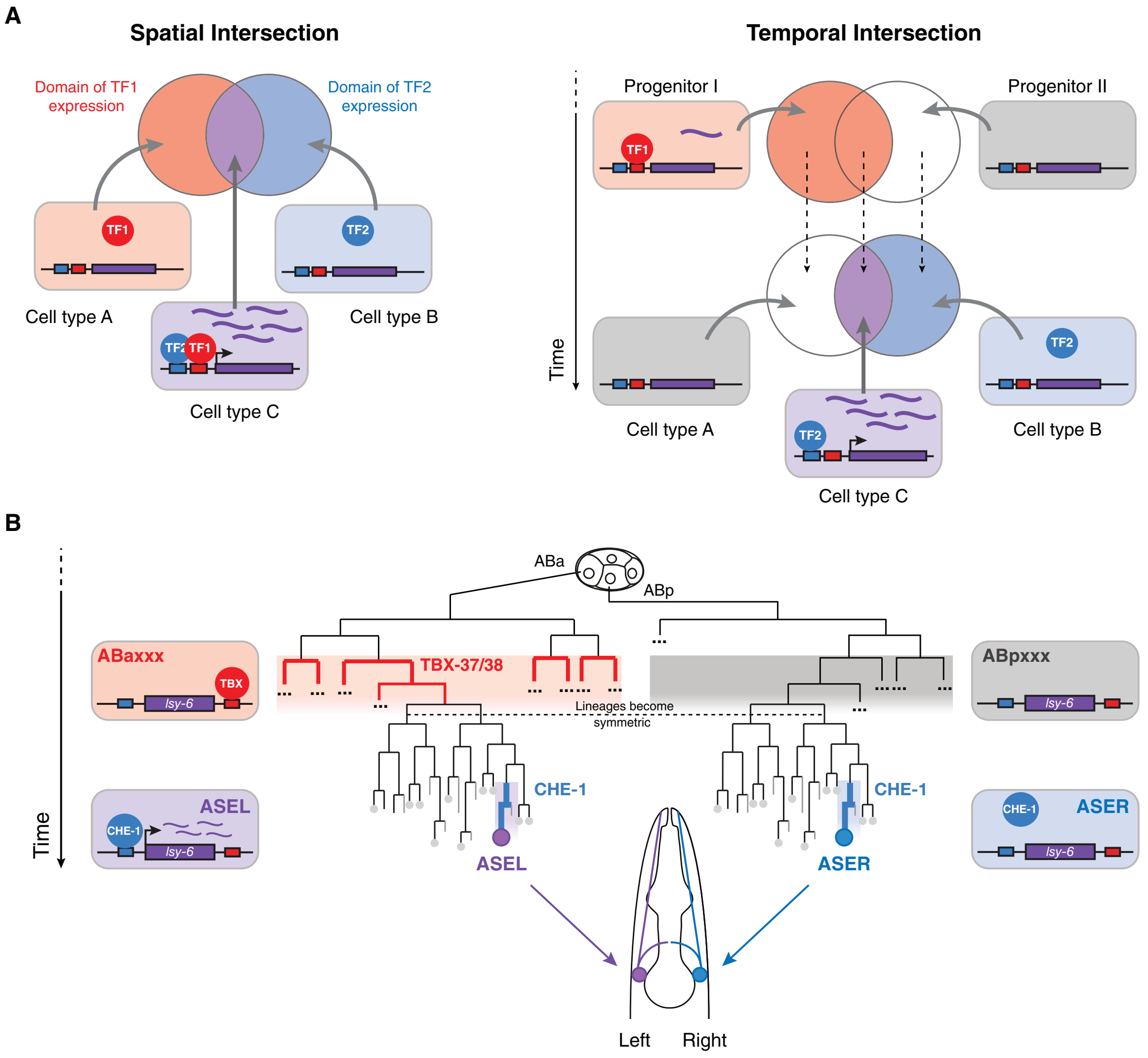

Figure 1. Integration of Transcriptional Inputs over Time as a Mechanism for Cell Diversification

(A) Left. Schematic of the classic mode of combinatorial TF action based on spatial intersection of two TFs. Circles denote the partially overlapping domains of expression of two TFs. Boxes represent cells within those domains. Expression of TF1 or TF2 alone is not sufficient to activate a gene relevant for cell identity (in purple) but action of both TFs together is. Right. Schematic of the concept of temporal intersection in which two TFs are required for transcriptional activation, but separated in time. TF1 is expressed transiently in a subset of progenitor cells, while TF2 is expressed in a subset of cells undergoing terminal differentiation. Only a cell expressing TF2 that derived from a progenitor that expressed TF1 will activate the purple gene. Therefore, the outcome of the presence of TF2 in a cell depends on the transcriptional history of that cell, and this mechanism can contribute to cellular diversification.

(B) Schematic of the lineage histories of the left and right ASE neurons (ASEL and ASER), which derive from the ABa and ABp blastomeres, respectively, at the 4-cell stage of embryogenesis. Both neurons express the terminal TF CHE-1 at the endpoint. CHE-1 is necessary for *lsy-6* expression, but only ASEL expresses *lsy-6*. Asymmetric expression of *lsy-6* is genetically dependent on the transiently expressed TBX-37/38, which have a predicted binding site in the *lsy-6* locus.

(Figure 1B) (Good et al., 2004). Early expression of TBX-37/38 is necessary for *lsy-6* transcription in ASEL four cell divisions later (Cochella and Hobert, 2012). In addition, a *cis*-regulatory element in the *lsy-6* locus, required for *lsy-6* transcription, contains two putative TBX-37/38 binding sites (Cochella and Hobert, 2012). These findings implied that TBX-37/38 act combinatorially with CHE-1 to activate *lsy-6* exclusively in ASEL. However, TBX-37/38 were reported to be transiently expressed four cell divisions before the onset of CHE-1 expression (Figure 1B) (Good et al., 2004), suggesting a model in which TBX-37/38 would prime the *lsy-6* locus at an early time point, in a lineage-specific manner, to enable subsequent activation by CHE-1 in ASEL (Cochella and Hobert, 2012). This model raised a number of mechanistic questions about how these two transcriptional inputs are integrated to achieve *lsy-6* expression.

Here, we took advantage of several recently available methods to experimentally test and dissect the mechanism of priming by TBX-37/38 and its temporal separation from the action of CHE-1. We demonstrate that TBX-37/38 act directly on *lsy-6* and that this activity is indeed only transiently required, providing experimental support for the combinatorial activity

of TBX-37/38 and CHE-1 on the *lsy-6* locus. Because these two TFs act on the same locus but have non-overlapping temporal expression and only partially overlapping spatial expression, we refer to this as "temporal intersection," in analogy to "spatial intersection" mechanisms (Figure 1A, right). We also show that the transient action of TBX-37/38 establishes a transcriptionally active state, which is necessary and sufficient to prime *lsy-6*. The asymmetric active transcriptional state of *lsy-6* can be maintained in the absence of the asymmetric trigger in a symmetric *trans*-acting factor environment, indicating that this is an epigenetic phenomenon. Finally, we provide evidence for the idea that, akin to spatial integration of TFs, temporal integration may be a general mechanism for the generation of cellular diversity during development. Our findings define a mechanistic framework for a mode of combinatorial TF activity with important implications for understanding development and for current efforts to generate defined cell types *in vitro*.

RESULTS

Direct, Early Binding of TBX-37/38 Is Necessary and Sufficient to Prime *lsy-6*

To begin to dissect the mechanism by which TBX-37/38 contribute to the specific expression of *lsy-6* in the post-mitotic ASEL, we explored the possibility that these TFs bind directly to the *lsy-6* locus already at the 28-cell stage, when they are first detected. We generated *gfp*-tagged alleles of endogenous *tbx-37* or *-38* using CRISPR-Cas9 (Figure S1A). Because these TFs are almost identical in sequence and fully redundant (Good et al., 2004), we tagged each TF in the background of a deletion of its paralog, such that the only source of TBX factor is tagged with GFP. N-terminal fusion of GFP did not cause defects in TBX activity, as animals developed normally, and were, thus, used for chromatin immunoprecipitation sequencing (ChIP-seq) from embryos staged at the peak of TBX-37/38 expression (90 min post 2-cell). *De-novo* motif discovery analysis of the TBX-37/38 binding sites revealed a highly enriched sequence motif practically identical to the known TBX-38 binding sequence (Figure 2A) (Narasimhan et al., 2015). Focusing on the *lsy-6* locus, we observed that both TBX-37/38 bound downstream of *lsy-6*, overlapping with the *cis*-regulatory element that had been genetically defined (Cochella and Hobert, 2012) (Figure 2A).

To functionally test the contribution of this binding site to *lsy-6* expression, we used CRISPR-Cas9 to delete a 150-bp region spanning two predicted TBX-37/38 binding sequence motifs (*Δtbs*). This deletion abolished transcription of endogenous *lsy-6* miRNA, whose activity we followed via expression of an ASER reporter: wild-type animals express *gcy-5* exclusively in ASER, but in animals lacking *lsy-6,* ASEL adopts the ASER fate and expresses *gcy-5* as well (Johnston and Hobert, 2003). Deletion of the *tbs* resulted in complete *lsy-6* loss of function, with all animals expressing the ASER marker in both ASE neurons (Figure 2B). Moreover, deletion of the *tbs* completely eliminated expression of a *yfp* reporter inserted in place of the *lsy-6* miRNA, also using CRISPR-Cas9 (Figure 2C). These data indicate that the requirement for TBX-37/38 is mediated through a binding site downstream of the *lsy-6* miRNA sequence.

It was previously shown that early expression of TBX-37/38 could prime a *lsy-6* reporter transgene for subsequent activation by CHE-1, but expression of TBX-37/38 after the birth of the ASE neurons was unable to do so (Cochella and Hobert, 2012). To define the ability of the TBXs to prime endogenous *lsy-6* over time, we ectopically expressed TBX-37 in all cells, at different time points (using a heat-shock promoter) and followed expression of the endogenously labeled *lsy-6::yfp*. If heat-shock-induced TBX-37 is sufficient to prime *lsy-6*, we expect YFP expression in ASER, in addition to ASEL. Heat-shock-activated TBX-37 was sufficient to prime *lsy-6* for expression in ASER up to the 180-min time point, resulting in embryos with YFP in both ASEs (Figure 2D). However, beyond this time point, TBX-37 progressively lost its ability to prime *lsy-6* in both neuron precursors and was ultimately unable to activate transcription of the *lsy-6* locus (Figure 2D). This defined a window during which priming needs to occur, beyond which *lsy-6* becomes refractory to transcriptional activation. Moreover, although TBX-37 was expressed throughout the embryo, YFP was expressed exclusively in the ASE neurons, supporting that *lsy-6* specificity is generated by the intersection with the ASE-specific CHE-1.

Together, these data show that early, direct action of TBX-37/38 on the *lsy-6* locus is necessary and sufficient to prime *lsy-6* for subsequent activation by CHE-1 in the ASEs.

TBX-37/38 Are Transiently Required to Prime *lsy-6*

Previous work suggested that TBX-37/38 expression is temporally separated from that of CHE-1, but it has remained unclear whether low levels of TBX-37/38 persist and continue to act on *lsy-6* at later time points. The onset of CHE-1 expression is in the mother of the ASE neurons, based on single-molecule fluorescence *in situ* hybridization (smFISH) and imaging of endogenously tagged CHE-1 (Cochella and Hobert, 2012; Leyva-Díaz and Hobert, 2019). TBX-37/38 were reported, based on immunostaining, to be expressed at the 8 ABa stage (when the ABa branch has produced 8 descendants) and at lower level one cell division later, at the 16 ABa stage (Good et al., 2004) (Figure 3A). We set out to further rigorously examine the temporal separation of these TFs.

First, we used the endogenous TBX-GFP fusions to follow expression of TBX-37/38 by GFP fluorescence and smFISH to visualize *tbx-37/38* transcripts. Our data largely confirmed the published spatio-temporal restriction of TBX-37/38 (Figures 3B and S1). We did, however, observe dim GFP fluorescence also at the 32 ABa stage, which was not reported by immunostaining. This suggests that either the GFP fusion provides a more sensitive read out or, alternatively, that it could stabilize TBX-37/38. Nevertheless, we did not detect fluorescence above background at later stages (Figures S1A and S1B). Given the onset of expression of CHE-1 in the mother of the ASE neurons, these data suggest that its action on the *lsy-6* locus is separated from TBX-37/38 by at least three, most likely four cell divisions, out of a total of 11 cell divisions from fertilized zygote to post-mitotic ASE (Sulston et al., 1983).

Second, to investigate whether TBX-37/38 might be continuously required even if these factors are expressed below the limit of detection, we designed a timed, forced degradation experiment. We targeted GFP-TBX-37 for degradation using an

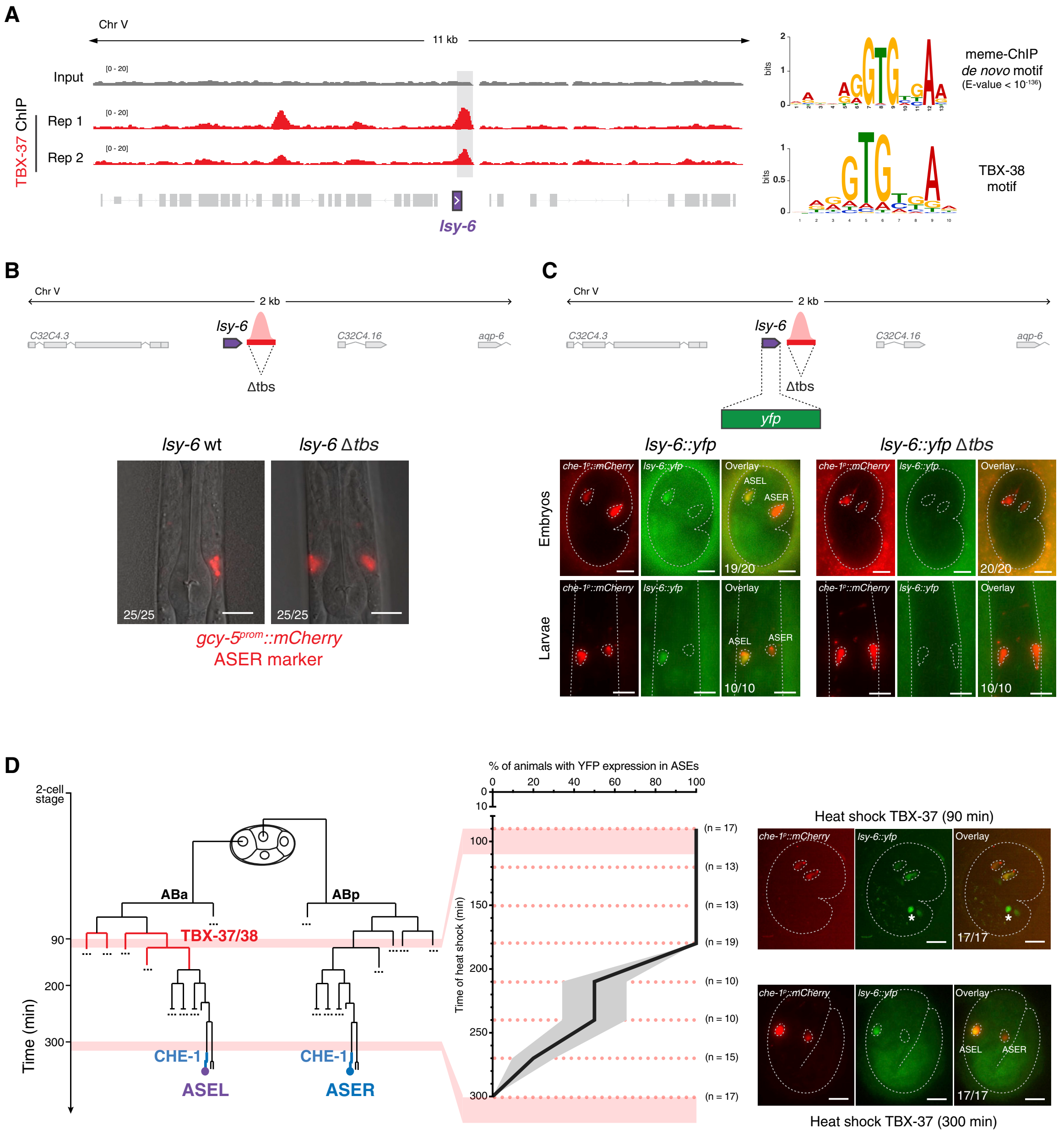

Figure 2. Early, Direct Binding of TBX-37/38 to *lsy-6* Is Necessary and Sufficient to Establish Competence for Transcription in the ASE Neurons

(A) GFP-TBX-37 ChIP-seq signal over the *lsy-6* locus and flanking regions obtained from embryos staged at the peak of TBX-37/38 expression (90 min post 2-cell); two biological replicates. A peak of GFP-TBX-37 slightly downstream of *lsy-6* (gray bar) was detected in all individual replicates (p value $< 10^{-5}$ in each replicate). *De-novo* motif discovery within TBX-37/38 binding sites, retrieved a highly enriched motif that matches the binding site for TBX-38 derived from *in vitro* binding studies (Narasimhan et al., 2015). The peak in the *lsy-6* locus overlaps two such motifs.

(B) Deletion of a 150-bp region containing the TBX-37/38 binding site (*Δtbs*) using CRISPR-Cas9, from the wild-type *lsy-6* locus, caused fully penetrant transformation of ASEL into ASER, monitored by expression of the ASER-specific marker *gcy-5^{prom}::mCherry* (n = 25 for each genotype).

(C) A *yfp* reporter inserted in the endogenous *lsy-6* locus via CRISPR-Cas9 resulted in exclusive YFP expression in ASEL in embryos and larvae (n = 20 and n = 10 for each genotype, respectively). Deletion of the TBX-37/38 binding site (*Δtbs*) in this context completely abolished expression of YFP at every stage. ASEs are marked by a genome-integrated *che-1^{prom}::mCherry* reporter. Representative images are shown.

(D) Embryos carrying the engineered *lsy-6::yfp* endogenous locus (C) and a *heat-shock^{prom}::tbx-37* transgene, were subjected to heat shock at the indicated times (dashed lines) (n ≥ 10 for each time point). Ectopic TBX-37 expression in the whole embryo at early time points induced additional *lsy-6::yfp* expression in ASER (*heat-shock treatment alone caused variable expression in two cells in the tail). Beyond the 180-min time point, TBX-37 progressively lost ability to activate *lsy-6::yfp* expression in ASER or any other cell. Plot shows proportion and standard error of proportion (SEP). Representative images are shown for the 90- and 300-min time points. All scale bars represent 10 μm.

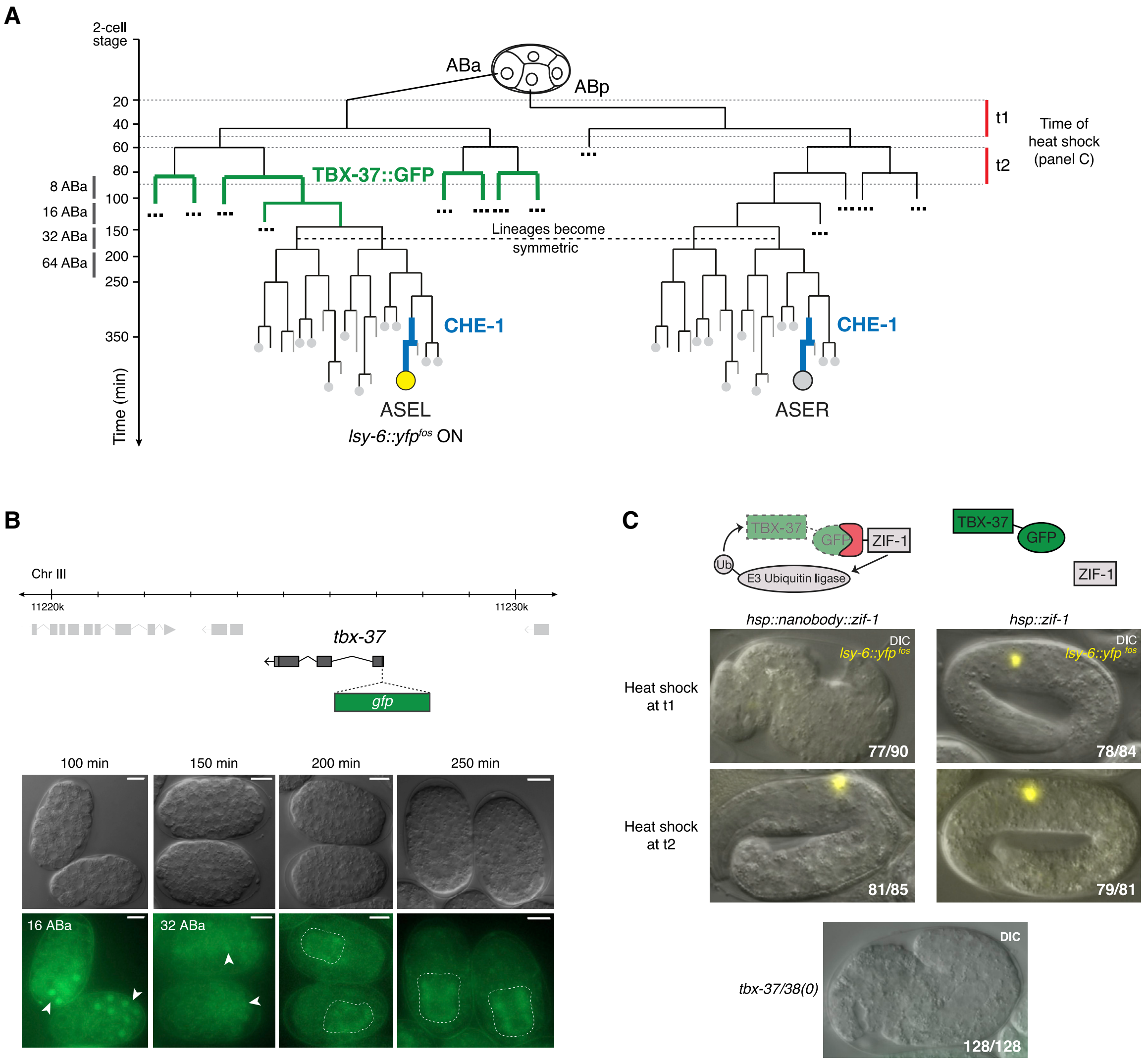

Figure 3. TBX-37/38 Are Not Continuously Required for *lsy-6* Expression

(A) Developmental lineage of ASEL and ASER with relevant timing information for (B) and (C).

(B) Expression of N-terminally tagged, endogenous TBX-37 (or TBX-38, Figure S1) was followed by GFP fluorescence over time. Representative images are shown. Fluorescence was clearly visible at 16 ABa stage (arrowheads) and was dim in a few nuclei at 32 ABa stage but no signal was visible beyond this time point (also Figures S2A and S2B). Autofluorescence from the developing gut is outlined with a dashed line. DIC (differential interference contrast) and fluorescence are shown. Scale bars represent 10 μm.

(C) Degradation of GFP-TBX-37 (in a *tbx-38(0)* background) was induced using an anti-GFP nanobody fused to the ubiquitin-ligase adaptor ZIF-1, expressed under control of a heat-shock promoter. Representative images show embryos heat shocked at two different times (see A): t1 targeted the peak of TBX-37/38 expression (Figure S2) and caused 77/90 embryos to phenocopy *tbx-37/38(0)* morphological defects and fail to express *lsy-6::yfp*^{fosmid}; t2 (onset of degradation 40 min later, Figure S2) caused no defects in morphology or *lsy-6* expression in 81/85 embryos. Heat shock of animals expressing ZIF-1 without the targeting nanobody had no effect at either time. Comparison of control and nanobody treatments was done using a chi-squared test: p value$_{t1}$ < 0.0001, p value$_{t2}$ = 0.297 (Table S1). DIC and fluorescence were overlaid in the same image.

anti-GFP nanobody fused to the ubiquitin-ligase adaptor ZIF-1 (Wang et al., 2017b). The nanobody-ZIF-1 fusion was expressed from a single-copy transgene (Frøkjaer-Jensen et al., 2012) under the heat-shock promoter, enabling inducible degradation of endogenous GFP-TBX-37, in a *tbx-38* deletion background. Upon induction at different time points, we assessed expression of a *lsy-6::yfp* fosmid reporter with 40 kb of genomic context (Cochella and Hobert, 2012), as well as the embryo morphology at the end of embryogenesis. Degradation of GFP-TBX-37 at 8–16 ABa (t1) caused penetrant loss of *lsy-6* expression and phenocopied complete TBX-37/38 loss of function, with embryos lacking the anterior part of the pharynx and showing severely impaired morphogenesis (Good et al., 2004) (Figures 3C and S2). However, if the onset of GFP-TBX-37 degradation was 40 min later (t2), roughly the duration of a cell cycle, there was no effect on either *lsy-6* expression or morphogenesis (Figures 3C and S2). We further confirmed that the degradation system works efficiently at both time points by also monitoring

degradation of an abundant GFP-tagged TF, PHA-4, which is expressed in many cells from the ABa lineage (Gaudet and Mango, 2002) (Figure S2). Moreover, a control strain expressing ZIF-1 without the anti-GFP nanobody did not display defects when subjected to heat shock at either time point.

Together, these data strongly support the early, transient requirement of TBX-37/38 for later expression of *lsy-6*.

TBX-37/38 Establish a Lineage-Specific Accessible State of the *lsy-6* Locus

We hypothesized that transient action of TBX-37/38 would establish a lineage-specific, competent chromatin state of *lsy-6*. Decompaction of a *lsy-6* transgene in ABa grand-daughters was previously reported to correlate with TBX-37/38 expression, using a lac-operator-tagging strategy (Cochella and Hobert, 2012). However, this technique did not allow to follow decompaction at later time points and lacked resolution to distinguish between different *cis*-regulatory elements within the *lsy-6* locus. We, thus, set out to specifically isolate cells derived from the two ASE-originating lineages (ABa and ABp) for analysis by ATAC-seq (Corces et al., 2017). We generated a transgenic strain carrying lineage-specific reporters (Figures 4A, S3A, and S3B), synchronized and dissociated embryos, and sorted viable cells from ABa or ABp based on fluorescence, at different time points (Figure S3C).

The earliest time point at which we could separate cells from the different lineages was at the onset of TBX-37/38 expression in the ABa lineage (~90 min post 2-cell). At this time point, genome-wide accessibility patterns of ABa- and ABp-derived cells are extremely similar (Pearson correlation coefficient = 0.95; Figure S3D). However, the *lsy-6* locus was clearly accessible only in ABa-derived cells, in a region overlapping with the TBX-37/38 binding site defined by ChIP-seq (Figures 4B and 4C). TBX-37/38 are likely the main determinants of differences in accessibility between ABa and ABp at this time, as *de-novo* motif discovery among ABa-specific accessible regions recovered the known TBX-38 motif as a clear top hit (Figure S3E). We also performed ATAC-seq at the 200-min time point and found that the specific difference in accessibility of the the *lsy-6* locus remains (Figures 4B, 4C, and S3D), although TBX-37/38 are no longer present at that time point. At this time, there are 64 descendants of each ABa or ABp. The substantial signal observed at the *lsy-6* locus in ABa descendants suggests that it must be accessible in a large fraction of ABa descendants.

At the time point when the ASEs are born (350 min), ABa and ABp have each produced ~180 descendants. When we sampled all of them, the *lsy-6* locus appeared equally inaccessible in both lineages (Figure 4B), even though these datasets were of high quality and globally highly correlated to the 200 min datasets (Pearson correlation coefficient = 0.85 for ABa and 0.87 for ABp). However, at this time most cells are differentiated, and we could not exclude that *lsy-6* remained accessible in the one ASEL neuron among the ~180 ABa descendants. To directly assess *lsy-6* accessibility in ASEs, we used fluorescence-activated cell sorting (FACS) to isolate the left and right neurons from embryos expressing cell-specific labels. The genome-wide patterns of accessibility between ASEL and ASER neurons are highly similar (Pearson correlation coefficient = 0.94) (Figure S3D). Both are largely determined by CHE-1, consistent with its known expression and function (Etchberger et al., 2007; Leyva-Díaz and Hobert, 2019; Uchida et al., 2003), as *de-novo* motif discovery among ASE-specific accessible regions recovered the known CHE-1 motif in the top five hits, both in ASEL and ASER (Figure S3E). However, ATAC-seq analysis revealed an accessible region upstream of *lsy-6* in ASEL, which was inaccessible in ASER (Figures 4B and 4C). The peak of accessibility in ASEL overlaps with a CHE-1 binding site that has been well-characterized genetically and biochemically (Etchberger et al., 2007, 2009). This suggests that TBX-37/38 binding during early development impacts later accessibility of the CHE-1 binding site. Further supporting this, accessibility of the CHE-1 binding region was fully lost upon deletion of the TBX-37/38 binding sites (*Δtbs*) (Figures 4C and 4D).

Together, these data indicate that TBX-37/38 establish an early asymmetry in *lsy-6* accessibility that impacts later ability of CHE-1 to bind and promote *lsy-6* transcription.

TBX-37/38 Promotes Bidirectional Transcription of *lsy-6*, which Is Necessary to Establish the Competent State

We envisioned two possible mechanisms for establishment of the *lsy-6* competent state by TBX-37/38. In the first, binding by TBX-37/38 is sufficient to induce accessibility, which is then relayed by other intermediate TFs that could keep the locus open for CHE-1, as it has been proposed for many pioneer TFs (Zaret and Carroll, 2011). In the second, TBX-37/38-activated transcription of *lsy-6* establishes the competent state and is required for later CHE-1-mediated boosting. Here, we set out to distinguish between these two possibilities.

Although robust transcription of *lsy-6* is only observed upon onset of CHE-1, previous smFISH of the *lsy-6::yfp* fosmid transgene revealed a low level of transcription from the *lsy-6* locus starting shortly after onset of TBX-37/38 expression (Cochella and Hobert, 2012). Given that TBX-37/38 bind downstream of *lsy-6*, we asked whether the locus was also transcribed in the antisense orientation. Indeed, many cells in the anterior part of the embryo displayed antisense transcription of the *lsy-6::yfp* fosmid transgene, at levels considerably higher than those of the sense transcription (Figures 5A and S4A). Both sense and antisense signals were fully dependent on TBX-37/38 and the *tbs*-containing region (Figure S4B). In wild-type embryos, antisense transcription persisted until the birth of ASEL, as determined by the presence of high-intensity nuclear foci (Figures 5A and S4A). At this stage, sense transcription became restricted to ASEL and dramatically increased in level, consistent with the onset of expression and known activity of CHE-1 (Etchberger et al., 2007, 2009), whereas antisense transcription remained at relatively low levels and in a few cells (Figure 5A).

To examine the function of early transcription at the *lsy-6* locus, we first attempted to bypass the requirement for TBX-37/38 with a general transcriptional activator. To this end, we replaced the region containing the two TBX-binding sites in the *lsy-6::gfp* fosmid reporter with five upstream activation sequence (UAS) binding sites, which recruit the *S. kudriavzevii* TF GAL4 (Wang et al., 2017a) (*lsy-6::gfp::Δtbs::5xUAS*) (Figure 5B). This reporter did not drive the GFP expression as expected, given the lack of the TBX-37/38 binding sites (Table S1). We then used the *tbx-37* promoter to drive expression of the GAL4 DNA binding domain alone (GAL4^{DBD}) or fused to the

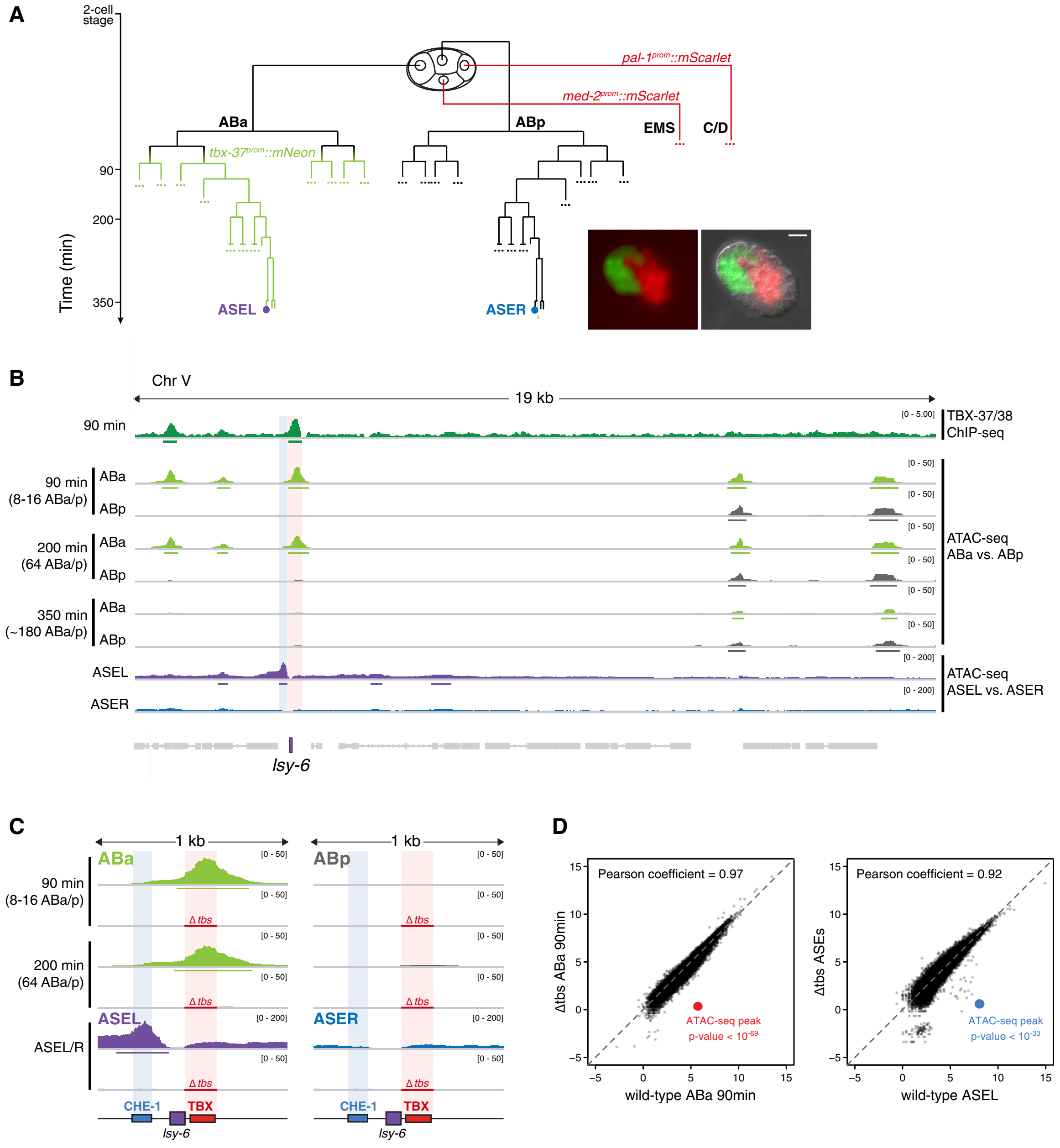

Figure 4. TBX-37/38 Establish a Differentially Accessible State of the *lsy-6* Locus

(A) Schematic of the embryo-labeling strategy for ABa and ABp isolation. The indicated time points (90, 200, and 350 min) were used for the experiments in (B) and (C). Representative image of an embryo at the 8 ABa stage carrying the three-reporter combination (Figures S3A–S3C).

(B) (Top) Aggregated GFP-TBX-37/38 ChIP-seq signal over the *lsy-6* locus and flanking sequences. (Middle) ATAC-seq signal in ABa- and ABp-derived cells at three different time points showing ABa-specific accessibility of *lsy-6*, overlapping with the TBX-37/38 binding site (red shading); two biological replicates were analyzed per condition. For reference, downstream locus shows equal accessibility in both lineages (also Figure S3D). Number of ABa/ABp descendants at the different time points are shown. (Bottom) ATAC-seq signal from sorted ASEL and ASER shows accessibility in ASEL upstream of *lsy-6*, overlapping with a CHE-1 binding site (blue shading). Peaks called by MACS2 are marked with a bar.

(C) Close-up view of the *lsy-6* locus and its ATAC-seq signal in ABa, ABp, ASEL, and ASER isolated from wild-type or *Δtbs* embryos (deletion marked with a red line); two biological replicates were analyzed per condition. Loss of TBX-37/38 binding sites causes loss of accessibility of the CHE-1 binding site in mature ASEs. For *Δtbs* embryos, ASEL and ASER cannot be distinguished as *lsy-6* is not expressed and the cells become symmetric (Figure 2B); the ASE ATAC-seq in this case was done on *che-1^{prom}::mCherry*-expressing cells, and the same track is shown in duplicate under ASEL and ASER.

(D) Correlation analysis between ATAC-seq data from wild-type or *Δtbs* embryos shows that datasets are highly similar and differ almost exclusively in their signal over the *lsy-6* locus. Plotted are signals in $\log_2$ (cpm) for all peaks called by MACS2 in at least one ATAC-seq sample. The p values for the called *lsy-6* peaks in ABa (overlapping with TBX-binding sites, red) and ASEL (overlapping with CHE-1 binding site, blue) are shown.

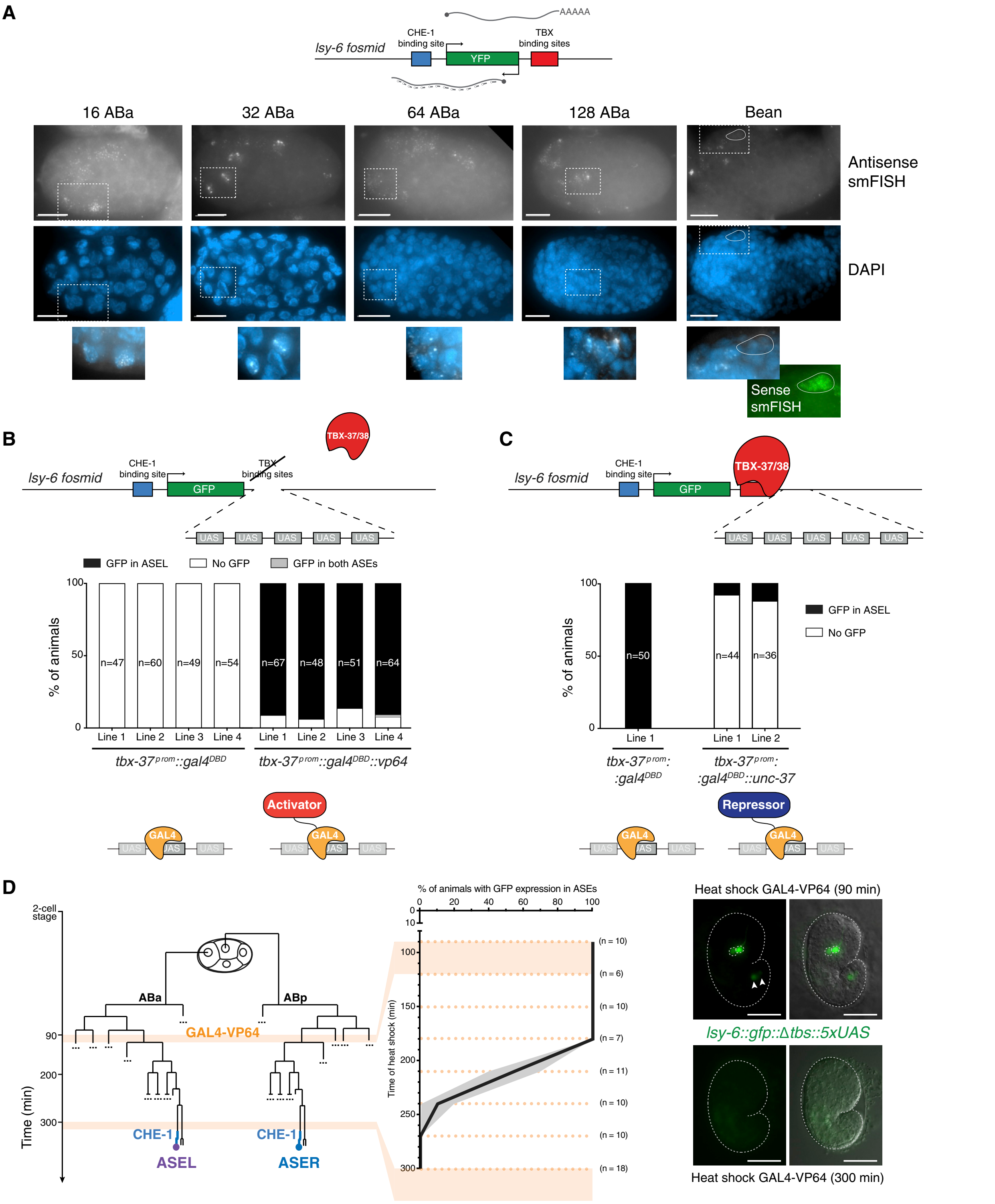

Figure 5. Transcription over the *lsy-6* Locus Occurs Bidirectionally and Is Required for Priming

(A) smFISH on embryos carrying the *lsy-6::yfp*^{fosmid} showed robust antisense transcription of the *lsy-6* locus (see Figure S4 for sense transcript and dependence on TBX-37/38 and their binding sites). Representative images are shown (n ≥ 10). Dashed boxes indicate zoomed regions showing bright nuclear foci. At the bean stage, the mature ASEL neuron is outlined as determined by strong signal against the sense *yfp* transcript. Scale bars represent 10 μm.

(B) The TBX-37/38 binding sites were replaced by five UAS sites in the context of the *lsy-6::gfp* fosmid reporter. YFP expression was restored exclusively in ASEL by expression of the GAL4^{DBD}-VP64 transcriptional activator under the *tbx-37*^{prom} (Table S2). Four independent, extrachromosomal transgenic lines were scored for each condition. Number of animals scored per line are shown.

(legend continued on next page)

CellPress
OPEN ACCESS

VP64 transcriptional activator (GAL4^{DBD}-VP64), consisting of four copies of the VP16 minimal activation domain (Beerli et al., 1998). GAL4^{DBD}-VP64 restored the exclusive expression of GFP in ASEL, whereas the GAL4^{DBD} alone did not (Figure 5B). These data suggest that early transcriptional activation of the *lsy-6* locus is sufficient for the establishment of the competent state, and further suggest that TBX-37/38 are not uniquely required to prime the *lsy-6* locus, as their function can be replaced by a heterologous, unrelated transcriptional activator.

We further validated that this heterologous system replicates the expression pattern of *lsy-6* through the combinatorial action of an early primer and later activation by CHE-1. To this aim, we asked if ectopic expression of GAL4^{DBD}-VP64 using a heat-shock promoter would replicate the time window for priming observed for TBX-37 (Figure 2D). Almost indistinguishably from TBX-37, GAL4^{DBD}-VP64 was sufficient to prime *lsy-6::gfp::Δtbs::5xUAS* during the first ~180 min of development but not beyond this time point (Figure 5D). Priming by GAL4^{DBD}-VP64 also recapitulated the specificity of *lsy-6* activation: even though this activator is expressed in all embryonic cells upon heat shock, we observed GFP expression exclusively in the ASE neurons suggesting that specificity in this heterologous system is also ultimately determined by CHE-1 (Figure 5D). Consistent with this, expression of the reporter fails to be boosted in absence of CHE-1 (Figures S4C and S4D).

Based on the finding that early transcriptional activation seems sufficient for priming, we hypothesized that blocking this early transcription, via the recruitment of a transcriptional repressor, would impair it. Briefly, we inserted five UAS sites downstream of the intact TBX-37/38 binding sites in the *lsy-6::gfp* fosmid reporter (*lsy-6::gfp::5xUAS*) and used the *tbx-37* promoter to drive expression of GAL4^{DBD} or GAL4^{DBD} fused to the transcriptional repressor Groucho/UNC-37 (GAL4^{DBD}-UNC-37) (Chambers et al., 2017; Kaul et al., 2014) (Figure 5C). Absence of tethering, or tethering of GAL4^{DBD} alone, did not affect *lsy-6* expression (Table S1; Figure 5C). However, early recruitment of GAL4^{DBD}-UNC-37 repressor to the UAS sites abolished GFP expression in ASEL (Figure 5C). We conclude that early adoption of a transcriptionally active state is necessary and sufficient to prime *lsy-6* for later robust activation and explains the requirement for TBX-37/38.

The Activity of TBX-37/38 Is Not Relayed by Other T-Box Transcription Factors

Given that TBX-37/38 bind the *lsy-6* locus early and are not continuously required, we set out to investigate how this competent state of *lsy-6* is maintained. Based on the continuous transcription over the *lsy-6* locus, we hypothesized that the competent state would be maintained by intermediate TFs, likely accompanied by a specific chromatin state. We envisioned that other T-box TFs could relay the initial activation by TBX-37/38, based on observations that sequentially expressed Sox TFs can bind to the same enhancer at different times during neurogenesis (Bergsland et al., 2011). The *C. elegans* genome encodes 22 T-box TFs, and many of them share similar binding sites (Narasimhan et al., 2015; Okkema, 2017). Theoretically, other T-box factors could use the same TBX-37/38 binding sites at later time points. Moreover, we found TBX-37/38 binding sites in the vicinity of six *tbx* genes (including *tbx-37* and *tbx-38*), which could be putative targets of TBX-37/38 activation and, thus, be expressed in the ABa lineage (Figure S5A). To determine whether other T-box TFs propagate the competent state of *lsy-6* over time, we followed expression of reporters for most T-box TFs through *C. elegans* development. For factors expressed before the ASE neurons are born, we traced expression throughout the whole lineage, with single-cell resolution (Figure S5B). We found seven T-box TFs expressed in the ABalpp lineage branch that gives rise to the ASEL neuron. To examine their potential role in *lsy-6* transcription, we analyzed *lsy-6* expression or function upon depletion of T-box factors either by RNA interference (RNAi) or by crossing to mutant alleles. Because some of these T-box factors occur in recently duplicated pairs, we also included conditions where two factors were ablated at the same time. None of the tested manipulations for individual or pairs of T-box TFs resulted in loss of *lsy-6* expression (Figures S5C and S5D). These results strongly argue that the asymmetric active state of *lsy-6* is not maintained by a cascade of T-box TFs.

The Asymmetric Competence of *lsy-6* Is Maintained in a Symmetric *Trans*-Acting Factor Environment

The competent state of *lsy-6* could be maintained by two distinct classes of factors. On one hand, it could be maintained by other TFs (or regulatory factors) that are induced by TBX-37/38 and are, thus, asymmetrically expressed across ABa and ABp. These factors (or their targets) could ultimately be present in ASEL (but not ASER) and act together with CHE-1 to activate *lsy-6* in a more conventional, cooperative manner (Figure 1A). In this model, TBX-37/38 would play both a *direct* role in establishing the *lsy-6* active state and an *indirect* role in maintaining this state through the activity of one or more of their transcriptional targets. Alternatively, TBX-37/38 could provide an asymmetric trigger that could be propagated by symmetrically expressed factors in cells that develop through paths that share extensive gene expression similarity: the two branches that will give rise to ASEL and ASER (ABalppp and ABpraaa) become symmetric around the 32 AB stage, share common gene-expression profiles over time (Poole et al., 2011; Sarin et al., 2009), and eventually give rise to the same set of 13 cells on each side of the head. The *lsy-6* competent state could, thus, be maintained by TFs and other regulators present in both lineage branches, but only if the locus was previously primed.

(C) Five UAS sites were inserted downstream of the TBX-37/38 binding sites in the context of the *lsy-6::gfp* fosmid reporter. Expression of GAL4^{DBD}-UNC-37 under the *tbx-37^{prom}* prevented later expression in ASEL (Table S2). Two independent, extrachromosomal transgenic lines were scored.

(D) Embryos carrying the integrated *lsy-6::gfp::Δtbs::5xUAS* reporter and a transgene containing GAL4^{DBD}-VP64 under a heat-shock promoter were subjected to heat shock at the indicated times (dashed lines). GAL4^{DBD}-VP64 expression in the whole embryo at early time points induced *lsy-6::gfp::Δtbs::5xUAS* expression in both ASEs. Beyond the 180-min time point, GAL4^{DBD}-VP64 progressively lost ability to activate *lsy-6::gfp::Δtbs::5xUAS* expression. Plot shows proportion and SEP (n ≥ 6 per time point). Representative images are shown for the 90- and 300-min time points. Arrowheads point to sporadic ectopic expression in two cells in the tail upon heat-shock treatment (independently of GAL4-VP64 expression). Scale bars represent 20 μm.

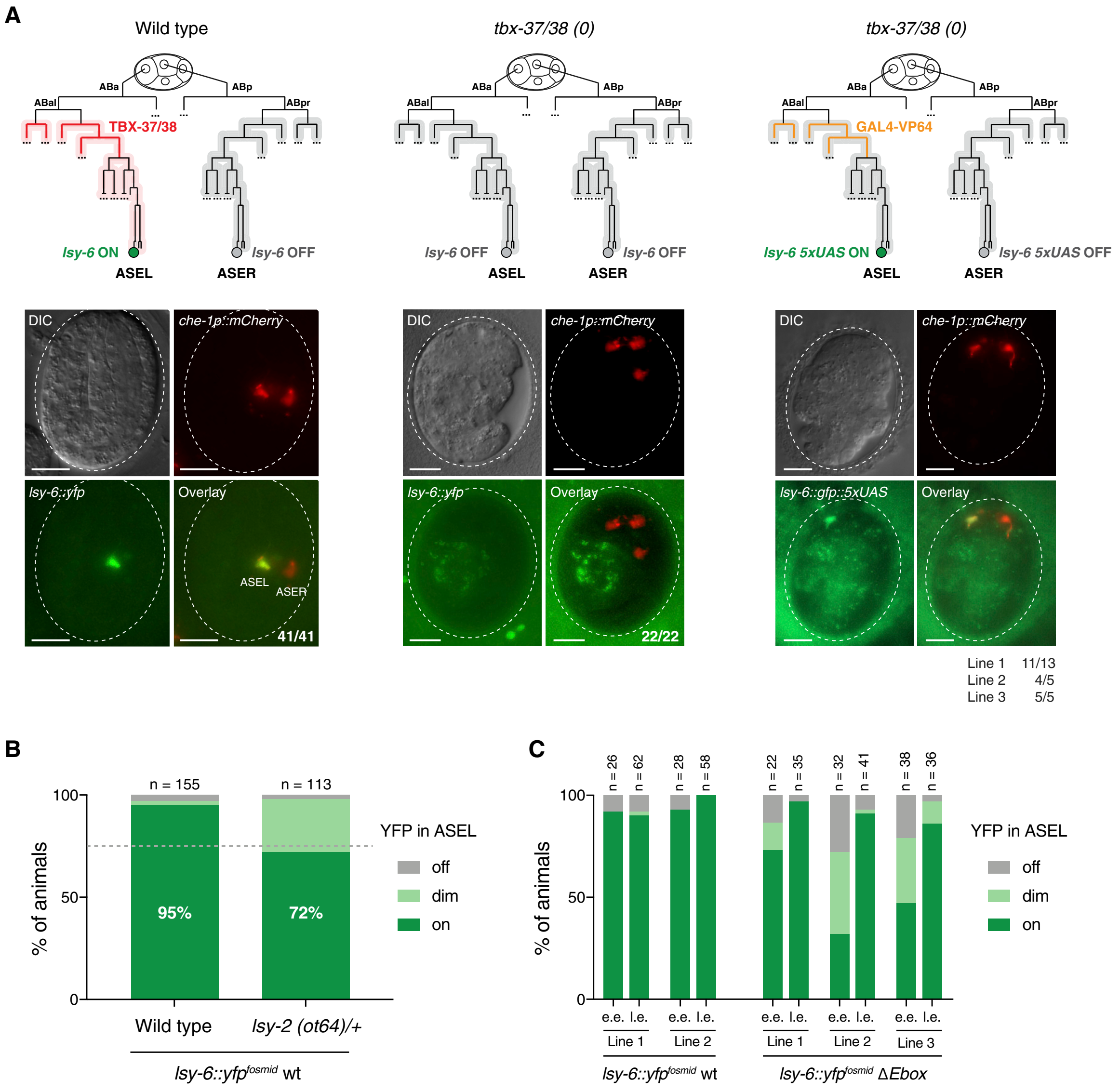

Figure 6. Asymmetric *lsy-6* Competence Is Maintained in a Symmetric *Trans*-acting Factor Environment

(A) Schematics of the developmental lineage and representative images of embryos of the indicated genotypes. The ASE neurons are marked by *che-1*^{prom}*::mCherry*. (Left) 41/41 wild-type embryos express the *lsy-6::yfp*^{fosmid} reporter in ASEL. Light-red highlight of the lineage indicates potential downstream asymmetries triggered by TBX-37/38. (Middle) Deletion of *tbx-37/38* resulted in 22/22 embryos without *lsy-6::yfp*^{fosmid} expression. (Right) Heterologous, asymmetric priming using the *lsy-6::gfp::Δtbs::5xUAS* + *tbx-37*^{prom}*::gal4::vp64* system (Figure 5B) was sufficient to activate *lsy-6::yfp* expression in only one ASE neuron in *tbx-37/38(0)* animals. Three independent transgenic lines were scored, numbers of embryos are shown. All scale bars represent 10 μm.

(B) The ubiquitous TF *lsy-2* is necessary for robust *lsy-6::yfp*^{fosmid} expression in ASEL (Table S3). Progeny of *lsy-2* heterozygous mothers were scored as larvae for YFP expression and compared with wild type. 25% of larvae are expected to be homozygous for *lsy-2*, setting the maximum possible effect. Animals scored as "dim" had a barely visible signal. n ≥ 113 per genotype.

(C) Deletion of an E-box in the *lsy-6* locus causes a delay in onset of *lsy-6::yfp* expression. Expression was scored in embryos from comma to 2-fold stage (early embryos, e.e.) or at the 3-fold stage (late embryos, l.e.). Three independent extrachromosomal lines show a delay in onset of YFP expression, n ≥ 22 per condition (Table S3).

To distinguish between these two possibilities, we asked whether asymmetrically primed *lsy-6* could retain competence in complete absence of TBX-37/38, which ensures the two branches develop symmetrically from an early time point and eliminates any possible indirect effect of TBX-37/38. To do this, we took advantage of the heterologous priming of the *lsy-6::gfp::Δtbs::5xUAS* reporter by the *tbx-37*^{prom}-driven GAL4^{DBD}-VP64 (Figure 5B) and asked whether ABa-specific priming can be maintained in a *tbx-37/38* double-mutant background. In embryos carrying *tbx-37/38* deletions, ABa adopts an ABp identity, and its descendants produce one or sometimes two ASE neurons that express ASER markers (Poole and Hobert, 2006) and never express *lsy-6* (Figure 6A). We found that *tbx-37*^{prom}-driven GAL4^{DBD}-VP64 activated the *lsy-6::gfp::Δ150::5xUAS* reporter in one (or sometimes two out of three ASE neurons) in the *tbx-37/38* null background, indicating

that asymmetric priming of *lsy-6* could be maintained in a symmetric *trans*-acting factor environment (Figure 6A). These results indicate that other TBX-37/38 targets are not necessary for maintenance of *lsy-6* competence in ABa descendants, and if additional factors are indeed required, they must be symmetrically expressed across ABa and ABp.

These findings provide an explanation for the observation that genetic screens recovered mutations in a number of symmetric *trans*-acting factors that cause loss of ASE neuron asymmetry (Sarin et al., 2007). For example, LSY-2 is a zinc finger TF that is ubiquitously expressed yet necessary specifically for ASEL identity (Johnston and Hobert, 2005). Given that *lsy-2*-deficient animals are sterile, we examined the progeny of heterozygous mothers and found that they failed to robustly express the *lsy-6::yfp* fosmid reporter (Figure 6B). Moreover, a consensus binding motif for a bHLH TF is necessary for early onset of *lsy-6::yfp* expression (Figure 6C), suggesting that a TF of this family may also be necessary to efficiently relay the competent state. A good candidate is the symmetrically expressed HLH-14, which is necessary to specify the neuronal identity of the ASEs (Poole et al., 2011). Unfortunately, we could not test the role of HLH-14 in *lsy-6* expression, as in *hlh-14* mutant animals the ASE grandmother cells fail to adopt their neuroblast identity and instead become epidermal cells (Poole et al., 2011). In addition, two ubiquitously expressed chromatin modifying complexes: the Set1/COMPASS complex, which acts as a histone H3K4 methyltransferase, and a complex containing a MYST histone acetyltransferase are necessary for *lsy-6* transcription (O'Meara et al., 2010; Poole et al., 2011). Together, these are good candidates for propagation of the *lsy-6* competent state until the onset of CHE-1 expression.

TBX-37/38 Regulates Left/Right Asymmetric Gene Expression in Additional Neuron Pairs

TBX-37/38 and CHE-1 activities are integrated on the *lsy-6* locus, resulting in ASEL-specific transcription. We postulated that TBX-37/38 might prime other loci to achieve asymmetric gene expression in other neuron pairs derived from the ABa and ABp lineages (Figure 7). To assess this, we examined the expression pattern of the gene downstream of *lsy-6*, C32C4.16 (Figure 7A), which shares the TBX-37/38 binding sites with *lsy-6* and is expressed in multiple neurons. A fosmid-based reporter for C32C4.16 was combined with a red fluorescent ASE marker (*che-1^{prom}::mCherry*) that enables the distiction between the left and right sides of the head based on axon morphology. Indeed, we found that C32C4.16 was not only asymmetrically expressed across the ASE neurons but also showed expression in a number of additional nuclei on the left side of the head of the worm, without obvious counterparts on the right side (Figure 7A).

To identify the precise neurons in which C32C4.16 is expressed, we took advantage of the NeuroPal system, which uses a combination of >40 neuron-specific markers in three different colors, making neurons easily identifiable by their unique color combination and position (Yemini et al., 2019). We identified an additional five neuron pairs with dual-lineage origin that display C32C4.16 expression in the left, but not the right member of the pair: AUAL, AWBL, AVEL, RMDL, and OLLL (Figure S6). Notably, none of these neuron pairs were previously known to display any molecular asymmetry, but they are all specified by bilaterally expressed terminal selectors akin to CHE-1 in the ASE neurons (Pereira et al., 2015; Serrano-Saiz et al., 2013). We confirmed the asymmetric expression across the AVE and AUA pairs by combining the C32C4.16 reporter with red fluorescent reporters expressed bilaterally in these neurons (Figure 7B).

Consistent with TBX-37/38 dependence, neuron pairs in which both members arise from ABa showed symmetric expression of C32C4.16 (e.g., AVJL/R, SMDVL/R). We further tested this dependence by crossing the C32C4.16 fosmid reporter into the *tbx-37/38(0)* background and by generating a fosmid reporter in which we deleted the TBX-37/38 binding sites. In both cases, expression of the reporter was lost in most cells (Figure 7C). Together, these observations suggest that priming by TBX-37/38 is integrated with other, yet to be identified TFs to achieve directed molecular asymmetries in the *C. elegans* nervous system (Figure 7D).

To define additional candidate loci that could be regulated in a manner similar to *lsy-6* and C32C4.16, we further mined the GFP-TBX ChIP-seq and ABa and ABp ATAC-seq datasets described above. We identified 86 genomic regions bound by TBX-37/38 and accessible in ABa, but not in ABp, at the 90-min time point. We assigned these to the nearest promoter, defining 86 genes, including protein-coding genes but also, intriguingly, two additional miRNAs. These genes form a set of compelling candidate loci that could be similarly primed by TBX-37/38 and could contribute to additional asymmetries among these neurons, but also among other ABa and ABp-derived lineage branches (Figure S6C).

DISCUSSION

A Molecular Mechanism for Temporal Integration of Transcriptional Inputs

The use of TF combinations for gene activation is a widespread phenomenon that has been long recognized to enable generation of cell-type-specific gene-expression patterns and, in turn, cell-type diversity. Here, we revealed mechanistic insight into how two temporally segregated TFs can act combinatorially to achieve cell-type specific transcription of a locus that controls neuronal identity. We also provide evidence that temporal integration of TFs may be a more general mechanism for cellular diversification.

The transiently expressed, lineage-specific TFs, TBX-37/38, bind directly to the *lsy-6* locus and promote an accessible state in cells that derive from the ABa blastomere. In contrast, in ABp descendants that do not express TBX-37/38, the *lsy-6* locus remains inaccessible throughout embryogenesis. Binding of TBX-37/38 promotes a transcriptionally active state, which primes *lsy-6* for later activation by CHE-1. Early transcription is primarily in the antisense orientation and does not produce active *lsy-6*. TBX-37/38 are not expressed or functionally required beyond the priming event, and no other downstream targets of TBX-37/38 are needed to establish or maintain the competence of the *lsy-6* locus. CHE-1 expression in the mother of both ASE neurons boosts *lsy-6* expression, specifically in ASEL. The locus remains inaccessible and refractory to activation in ASER despite the presence of CHE-1, which is otherwise able to promote the

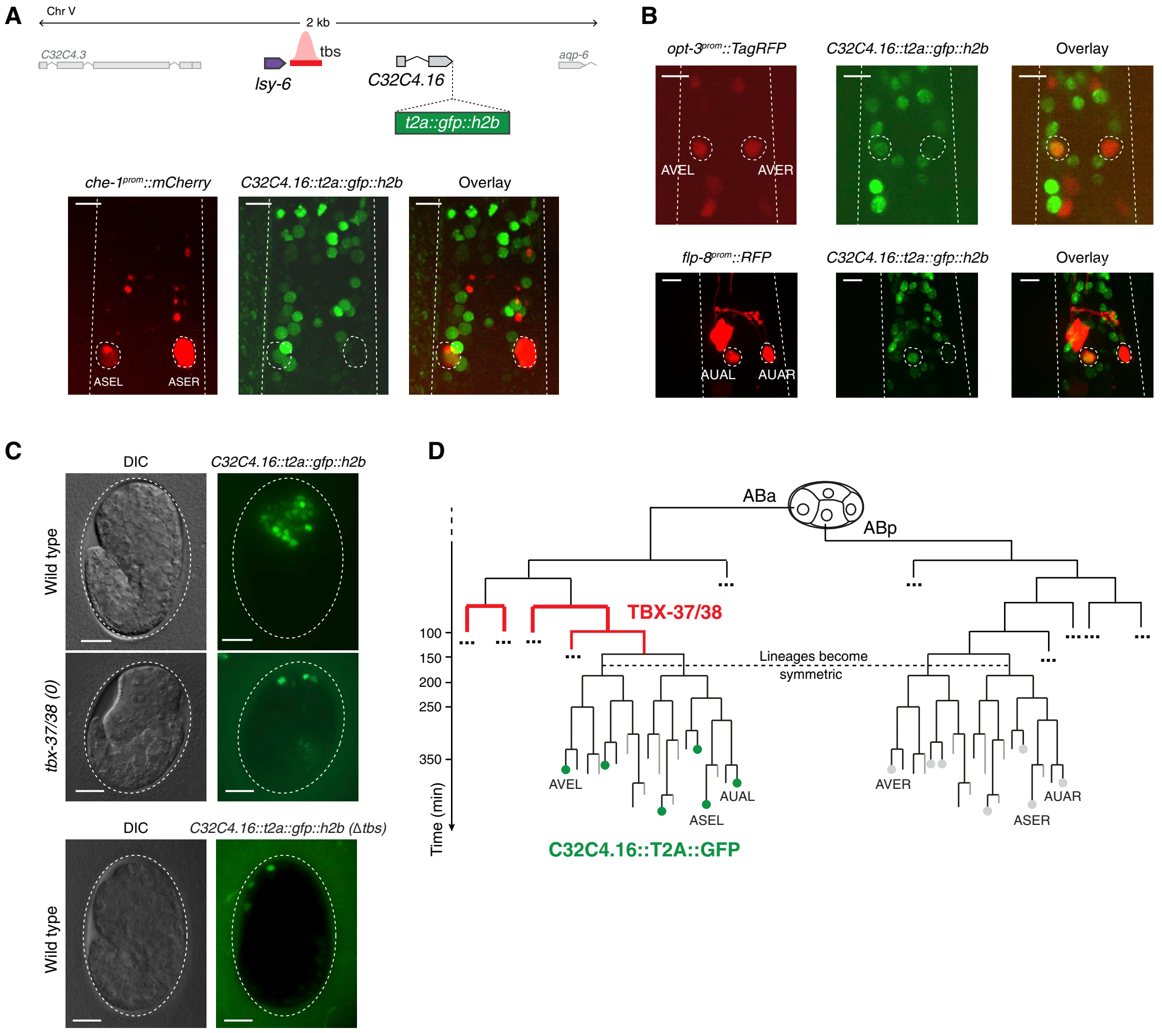

Figure 7. TBX-37/38 Determine Left-Right Molecular Asymmetry across Multiple Bilateral Neuron Pairs with ABa/ABp Lineage Origin
(A) Schematic of the reporter used to monitor expression of C32C4.16 (in the context of a fosmid clone containing ~40 kb of flanking genomic sequence), producing nuclear GFP separated from the C32C4.16 protein product through a 2A peptide (Ahier and Jarriault, 2014). Representative images show a larval head with left-specific expression across ASE neurons (labeled with *che-1^{prom}::mCherry*) and potentially other neurons on the left side.
(B) C32C4.16 expression is also lateralized across AVE and AUA neuron pairs (labeled with *opt-3^{prom}::TagRFP* and *flp-8^{prom}:: RFP*, respectively) (n = 10 per genotype).
(C) Expression of C32C4.16 requires TBX-37/38 and their binding sites for the majority of its expression pattern (n = 20 per genotype). All scale bars represent 10 μm.
(D) Schematic of the lineage origin of the AVE, AUA, and other neuron pairs that were also identified as having asymmetric C32C4.16 expression (Figure S6).

transcription of hundreds of other genes in this neuron (Etchberger et al., 2007). We propose that the transcriptionally active state in the ASEL precursors prevents *lsy-6* from adopting a repressed, inaccessible state that remains molecularly undefined.

The role of TBX-37/38 may seem similar to that of pioneer TFs, which are defined by their ability to bind nucleosomal DNA and unmask binding sites for other TFs (Zaret and Carroll, 2011; Zaret and Mango, 2016). However, we argue that there are important mechanistic distinctions between the two; unlike pioneer TFs, which initiate chromatin remodeling within inactive chromatin, TBX-37/38 must act during a permissive time window, before the inactive state sets in. Supporting this, a recent large-scale screen for TFs that can bind nucleosomal DNA suggested that TFs of the immunoglobulin superfamily, such as T-box factors, lack the molecular features that would enable pioneer function (Fernandez Garcia et al., 2019). A different study, however, showed some T-box factors can bind nucleosomes when reconstituted with DNA containing two binding sites on adjacent DNA gyres (Zhu et al., 2018). Nevertheless, our data indicate that TBX-37/38 fail to activate the *lsy-6* transcription once the locus becomes restricted and, thus, do not seem to act as pioneer TFs.

The ability of pioneer TFs to bind within inactive chromatin may explain why many of them do not need to activate transcription to establish accessibility (Sartorelli and Puri, 2018; Zaret and Carroll, 2011; Zaret and Mango, 2016). In fact, the best-studied

pioneer TFs have been shown to bind at least hours before the onset of transcription, which is typically triggered by the additional binding of other TFs to their exposed sites (e.g., Charney et al., 2017; Gualdi et al., 1996). The pioneer TF PHA-4, the *C. elegans* homolog of FoxA, does recruit RNA polymerase II (RNA pol II). However, this remains poised at the TSS and transcription occurs only at a later time point (Hsu et al., 2015). In contrast, our work suggests that binding of TBX-37/38 alone may not be sufficient, but that immediate transcriptional activation is necessary to impart competence to the *lsy-6* locus.

An important consequence of this distinction between the priming we describe and the action of pioneer TFs becomes evident in the context of efforts to program and reprogram different cell types *in vitro*. Whereas pioneer TFs are able to remodel the chromatin state of the cells to be reprogrammed (e.g., the pioneer activity of the Yamanaka factors has been studied in depth *in vivo* and *in vitro*; Iwafuchi-Doi and Zaret, 2016; Soufi et al., 2015), priming events that need to occur under specific chromatin contexts may not be fully recapitulated by protocols in which cells do not undergo the transcriptional histories they normally experience during development.

Transcriptional Activation as an Integral Player for Establishing Competence

How could establishing an active transcriptional state determine later competence for robust transcription? A role for transcription in establishing a competent epigenetic state was described for *Zdbf2* in mouse embryos (Greenberg et al., 2017), which requires early embryonic transcription from an alternative, upstream promoter to become competent for transcription during adulthood. The early transcription promotes DNA methylation (on cytosines) and this in turn evicts H3K27me3 marks that cover the adult promoter and prevents re-establishment of this mark by Polycomb. In absence of early transcription, the adult promoter remains silenced by Polycomb (Greenberg et al., 2017). The system we describe here cannot rely on cytosine methylation as this mark does not occur in *C. elegans*. Adenosine methylation has been shown to occur in *C. elegans* but at levels at least an order of magnitude lower than cytosine methylation in mammals (0.01%–0.4% of adenosines) (Greer et al., 2015). Relying on DNA methylation enables maintenance of the active state across many cell divisions, from the embryo to the adult. In contrast, we suggest that the competent state that is established on *lsy-6* may require a more active maintenance mechanism, for example, through continuous, low-level transcription.

The recruitment and action of RNA pol II and associated factors during transcription initiation and elongation could change the chromatin landscape of the *lsy-6* locus either directly, by displacing nucleosomes (Venkatesh and Workman, 2015), or indirectly, through recruitment of histone modifiers. Elongating RNA pol II can recruit the Set1/COMPASS complex, which methylates histone H3 at position K4 (Ng et al., 2003). This modification has been shown to promote acetylation of histones on multiple different residues (Ginsburg et al., 2014; Noma and Grewal, 2002; Taverna et al., 2006) and inhibits methylation on H3K9 (Greenstein et al., 2020). Together, this can result in the destabilization of nucleosomes and prevent spreading of repressive chromatin onto transcriptionally active genes, all of which would favor active transcription (Greenstein et al., 2020). All components of the Set1/COMPASS complex were identified in a genetic screen as being necessary for robust *lsy-6* expression and establishment of ASE asymmetry (Poole et al., 2011). Our work provides a framework to further address the potential contribution of Set1/COMPASS to establishing and/or maintaining developmental competence.

The transcriptionally competent state of *lsy-6* needs to be maintained through three to four cell divisions. Our smFISH analysis revealed high-intensity nuclear foci that typically mark the site of active transcription, even at time points when TBX-37/38 are no longer active. We could discard the idea that continuous transcription is mediated by other targets of TBX-37/38 that may be asymmetrically expressed between ABa and ABp descendants. This indicates that if other TFs are necessary for maintenance of the active state, they are likely symmetrically expressed across both lineages. Broadly acting TFs could promote low levels of transcription of the *lsy-6* locus that, together with the active chromatin marks, may propagate the competent state until the onset of CHE-1.

Temporal Integration of Transcriptional Inputs as a General Mechanism for Cell Diversification

Priming of enhancers prior to the time point when robust transcription is required is a broadly observed phenomenon that can be caused by different mechanisms, e.g., pre-establishment of contact between promoter and distal enhancers (Ghavi-Helm et al., 2014; Ng et al., 2018; Rubin et al., 2017), action of pioneer TFs (Charney et al., 2017; Gualdi et al., 1996; Hsu et al., 2015; Kueh et al., 2016), acquisition of chromatin modifications (Creyghton et al., 2010; Rada-Iglesias et al., 2011), or as shown here by promoting early antisense transcription. However, this pre-activation step is most often studied and interpreted in the context of achieving robust and/or timely gene activation, by reducing the rate-limiting step of chromatin remodeling. For example, multiple enhancers carry histone modifications associated with activity (e.g., H3K4me1) in progenitor cells, even though they are not associated with robust transcription at that stage, but this is predictive for future transcription upon differentiation into specific cell types (Creyghton et al., 2010; Rada-Iglesias et al., 2011). In *Drosophila* embryos, priming of Notch-responsive enhancers enables a deterministic and sustained transcriptional response, whereas in the absence of priming, transcription is stochastic and "bursty" (Falo-Sanjuan et al., 2019).

Our work places these priming mechanisms under a different light. Lineage-specific priming can be used to generate unique temporal combinations of TFs with later-acting TFs that act both within and outside the lineage where priming occurred. Thus, we suggest that temporal integration of TFs, much like spatial integration, may be a more general mechanism for creating intersectional gene-expression patterns and, therefore, cellular diversity. One scenario in which this mechanism could be used in a manner analogous to what we describe for the ASE neurons is to diversify cells that develop through different trajectories but then acquire the same terminal identity, i.e., convergent cell types. Priming events would exploit transient differences in developmental trajectories, which if maintained through development could result in distinct

outcomes from the execution of an otherwise identical terminal differentiation program.

The *C. elegans* lineage reveals multiple cases of cells that seemingly adopt the same terminal fate, through expression of the same terminal TFs, but do so through developmental trajectories with distinct transcriptional histories (Sulston et al., 1983). Instances of such convergence are also known in vertebrate systems, e.g., the gut endoderm of the mouse is formed by intercalation of cells from embryonic and extra-embryonic lineages (Kwon et al., 2008), and multiple cell types can originate both from the neural crest or from mesodermal lineages (Dupin et al., 2018). Cases of convergence were until recently limited to specific systems studied in depth using classical lineage tracing; recent single-cell studies combining transcriptome and lineage analysis have revealed that cell-fate convergence is widespread during animal development (Chan et al., 2019; Konstantinides et al., 2018; Liu et al., 2019; Raj et al., 2018; Wagner et al., 2018). These cases provide a rich substrate to further explore the contribution of temporal integration of TFs to diversifying cell types.

Our work shows that priming does not require a unique activity of the transient TF, but the general ability to induce an active transcriptional state. Moreover, our data indicate that other terminal selectors, in addition to CHE-1, can "read out" the priming event. These observations make this mechanism highly versatile, and we, therefore, suggest this may be a more general, yet, unexplored source of cellular diversification.

STAR★METHODS

Detailed methods are provided in the online version of this paper and include the following:

- KEY RESOURCES TABLE
- RESOURCE AVAILABILITY
  - Lead Contact
  - Materials Availability
  - Data and Code Availability
- EXPERIMENTAL MODEL AND SUBJECT DETAILS
  - Strains
- METHOD DETAILS
  - Strain Generation
  - Fosmid Recombineering
  - Plasmid Construction
  - Chromatin Immunoprecipitation Sequencing (ChIP-Seq)
  - Worm Synchronization and Eggs Extraction
  - Chromatin Immunoprecipitation
  - Library Preparation
  - ChIP-seq Data Processing and Analysis
  - Single Molecule Fluorescence *In Situ* Hybridisation (smFISH)
  - Genetically Induced Protein Degradation
  - Assay for Transposase-Accessible Chromatin Using Sequencing (ATAC-seq)
  - Heterologous Gal4-UAS Tethering Assays
  - RNA Interference (RNAi)
  - Differential Interference Contrast and Fluorescence Microscopy
  - 4D Microscropy and Lineage Analysis
  - Confocal Microscopy
- QUANTIFICATION AND STATISTICAL ANALYSIS

SUPPLEMENTAL INFORMATION

Supplemental Information can be found online at https://doi.org/10.1016/j.devcel.2020.09.002.

ACKNOWLEDGMENTS

We thank Alexander Stark, Oliver Hobert, Angela Andersen (Life Science Editors), and the members of the Cochella Lab for feedback. Also, Sevinç Ercan for ChIP protocols, Paul Sternberg's lab for GAL4-UAS reagents, Manuel Zimmer's lab for neuronal markers, and WormBase. Some strains were provided by the CGC, funded by NIH Office of Research Infrastructure Programs (P40 OD010440).

Funding: This work was supported by an FP7/2007-2013 grant from the European Research Council to L.C. (ERC-StG-337161) and grants from the Austrian Science Fund (SFB-F43-23 and P 32636-B). J.C. received a scholarship from the Fonds de Recherche du Québec–Santé (35049). Basic research at IMP is supported by Boehringer Ingelheim GmbH.

AUTHOR CONTRIBUTIONS

J.C., T.D., and L.C. designed experiments, wrote the manuscript, and made figures with contributions from other authors. J.C. and T.D. conducted most of the experiments. A.M. and A.B. did the ATAC-seq experiments. J.W. did the bioinformatic analyses. M.A. generated strains and performed NeuroPal imaging. J.R. generated strains and conducted time course experiments. P.G.-P. carried out confocal microscopy.

DECLARATION OF INTERESTS

The authors declare no competing interests.

Received: April 27, 2020
Revised: July 30, 2020
Accepted: September 1, 2020
Published: September 30, 2020

REFERENCES

Ahier, A., and Jarriault, S. (2014). Simultaneous expression of multiple proteins under a single promoter in Caenorhabditis elegans via a versatile 2A-based toolkit. Genetics *196*, 605–613.

Alaynick, W.A., Jessell, T.M., and Pfaff, S.L. (2011). SnapShot: spinal cord development. Cell *146*, 178–178.e1.

Allan, D.W., and Thor, S. (2015). Transcriptional selectors, masters, and combinatorial codes: regulatory principles of neural subtype specification. Wiley Interdiscip. Rev. Dev. Biol. *4*, 505–528.

Askjaer, P., Ercan, S., and Meister, P. (2014). Modern techniques for the analysis of chromatin and nuclear organization in C. elegans. In WormBook: the online review of C. elegans biology, pp. 1–35, https://doi.org/10.1895/wormbook.1.169.1.

Beerli, R.R., Segal, D.J., Dreier, B., and Barbas, C.F. (1998). Toward controlling gene expression at will: specific regulation of the erbB-2/HER-2 promoter by using polydactyl zinc finger proteins constructed from modular building blocks. Proc. Natl. Acad. Sci. USA *95*, 14628–14633.

Bergsland, M., Ramsköld, D., Zaouter, C., Klum, S., Sandberg, R., and Muhr, J. (2011). Sequentially acting Sox transcription factors in neural lineage development. Genes Dev. *25*, 2453–2464.

Bindels, D.S., Haarbosch, L., van Weeren, L., Postma, M., Wiese, K.E., Mastop, M., Aumonier, S., Gotthard, G., Royant, A., Hink, M.A., and Gadella,

T.W.J., Jr. (2017). mScarlet: a bright monomeric red fluorescent protein for cellular imaging. Nat Methods *14*, 53–56.

Boeck, M.E., Huynh, C., Gevirtzman, L., Thompson, O.A., Wang, G., Kasper, D.M., Reinke, V., Hillier, L.W., and Waterston, R.H. (2016). The time-resolved transcriptome of C. elegans. Genome Res. *26*, 1441–1450.

Brenner, S. (1974). The genetics of Caenorhabditis elegans. Genetics *77*, 71–94.

Chambers, M., Turki-Judeh, W., Kim, M.W., Chen, K., Gallaher, S.D., and Courey, A.J. (2017). Mechanisms of Groucho-mediated repression revealed by genome-wide analysis of Groucho binding and activity. BMC Genomics *18*, 215.

Chan, M.M., Smith, Z.D., Grosswendt, S., Kretzmer, H., Norman, T.M., Adamson, B., Jost, M., Quinn, J.J., Yang, D., Jones, M.G., et al. (2019). Molecular recording of mammalian embryogenesis. Nature *570*, 77–82.

Charney, R.M., Forouzmand, E., Cho, J.S., Cheung, J., Paraiso, K.D., Yasuoka, Y., Takahashi, S., Taira, M., Blitz, I.L., Xie, X., et al. (2017). Foxh1 occupies cis-regulatory modules prior to dynamic transcription factor interactions controlling the mesendoderm gene program. Dev. Cell *40*, 595–607.e4.

Cochella, L., and Hobert, O. (2012). Embryonic priming of a miRNA locus predetermines postmitotic neuronal left/right asymmetry in C. elegans. Cell *151*, 1229–1242.

Conte, D., Jr., MacNeil, L.T., Walhout, A.J.M., and Mello, C.C. (2015). RNA interference in Caenorhabditis elegans. Curr. Protoc. Mol. Biol. *109*, 26.3.1–26.3.30.

Corces, M.R., Trevino, A.E., Hamilton, E.G., Greenside, P.G., Sinnott-Armstrong, N.A., Vesuna, S., Satpathy, A.T., Rubin, A.J., Montine, K.S., Wu, B., et al. (2017). An improved ATAC-seq protocol reduces background and enables interrogation of frozen tissues. Nat. Methods *14*, 959–962.

Creyghton, M.P., Cheng, A.W., Welstead, G.G., Kooistra, T., Carey, B.W., Steine, E.J., Hanna, J., Lodato, M.A., Frampton, G.M., Sharp, P.A., et al. (2010). Histone H3K27ac separates active from poised enhancers and predicts developmental state. Proc. Natl. Acad. Sci. USA *107*, 21931–21936.

Dupin, E., Calloni, G.W., Coelho-Aguiar, J.M., and Le Douarin, N.M. (2018). The issue of the multipotency of the neural crest cells. Dev. Biol. *444*, S47–S59.

Etchberger, J.F., Flowers, E.B., Poole, R.J., Bashllari, E., and Hobert, O. (2009). Cis-regulatory mechanisms of left/right asymmetric neuron-subtype specification in C. elegans. Development *136*, 147–160.

Etchberger, J.F., Lorch, A., Sleumer, M.C., Zapf, R., Jones, S.J., Marra, M.A., Holt, R.A., Moerman, D.G., and Hobert, O. (2007). The molecular signature and cis-regulatory architecture of a C. elegans gustatory neuron. Genes Dev. *21*, 1653–1674.

Falo-Sanjuan, J., Lammers, N.C., Garcia, H.G., and Bray, S.J. (2019). Enhancer priming enables fast and sustained transcriptional responses to Notch signaling. Dev. Cell *50*, 411–425.e8.

Fernandez Garcia, M., Moore, C.D., Schulz, K.N., Alberto, O., Donague, G., Harrison, M.M., Zhu, H., and Zaret, K.S. (2019). Structural features of transcription factors associating with nucleosome binding. Mol. Cell *75*, 921–932.e6.

Frøkjaer-Jensen, C., Davis, M.W., Ailion, M., and Jorgensen, E.M. (2012). Improved Mos1-mediated transgenesis in C. elegans. Nat. Methods *9*, 117–118.

Gaudet, J., and Mango, S.E. (2002). Regulation of organogenesis by the Caenorhabditis elegans FoxA protein PHA-4. Science *295*, 821–825.

Ghavi-Helm, Y., Klein, F.A., Pakozdi, T., Ciglar, L., Noordermeer, D., Huber, W., and Furlong, E.E.M. (2014). Enhancer loops appear stable during development and are associated with paused polymerase. Nature *512*, 96–100.

Ginsburg, D.S., Anlembom, T.E., Wang, J., Patel, S.R., Li, B., and Hinnebusch, A.G. (2014). NuA4 links methylation of histone H3 lysines 4 and 36 to acetylation of histones H4 and H3. J. Biol. Chem. *289*, 32656–32670.

Good, K., Ciosk, R., Nance, J., Neves, A., Hill, R.J., and Priess, J.R. (2004). The T-box transcription factors TBX-37 and TBX-38 link GLP-1/Notch signaling to mesoderm induction in C. elegans embryos. Development *131*, 1967–1978.

Greenberg, M.V.C., Glaser, J., Borsos, M., Marjou, F.E., Walter, M., Teissandier, A., and Bourc'his, D. (2017). Transient transcription in the early embryo sets an epigenetic state that programs postnatal growth. Nat. Genet. *49*, 110–118.

Greenstein, R.A., Barrales, R.R., Sanchez, N.A., Bisanz, J.E., Braun, S., and Al-Sady, B. (2020). Set1/COMPASS repels heterochromatin invasion at euchromatic sites by disrupting Suv39/Clr4 activity and nucleosome stability. Genes Dev. *34*, 99–117.

Greer, E.L., Blanco, M.A., Gu, L., Sendinc, E., Liu, J., Aristizábal-Corrales, D., Hsu, C.H., Aravind, L., He, C., and Shi, Y. (2015). DNA methylation on N6-adenine in C. elegans. Cell *161*, 868–878.

Gualdi, R., Bossard, P., Zheng, M., Hamada, Y., Coleman, J.R., and Zaret, K.S. (1996). Hepatic specification of the gut endoderm in vitro: cell signaling and transcriptional control. Genes Dev. *10*, 1670–1682.

Hobert, O. (2008). Regulatory logic of neuronal diversity: terminal selector genes and selector motifs. Proc. Natl. Acad. Sci. USA *105*, 20067–20071.

Hobert, O. (2014). Development of left/right asymmetry in the Caenorhabditis elegans nervous system: from zygote to postmitotic neuron. Genesis *52*, 528–543.

Hobert, O. (2016). Terminal selectors of neuronal identity. Curr. Top. Dev. Biol. *116*, 455–475.

Hostettler, L., Grundy, L., Käser-Pébernard, S., Wicky, C., Schafer, W.R., and Glauser, D.A. (2017). The bright fluorescent protein mNeonGreen facilitates protein expression analysis in vivo. G3 (Bethesda) *7*, 607–615.

Hsu, H.T., Chen, H.M., Yang, Z., Wang, J., Lee, N.K., Burger, A., Zaret, K., Liu, T., Levine, E., and Mango, S.E. (2015). Transcription. Recruitment of RNA polymerase II by the pioneer transcription factor PHA-4. Science *348*, 1372–1376.

Iwafuchi-Doi, M., and Zaret, K.S. (2016). Cell fate control by pioneer transcription factors. Development *143*, 1833–1837.

Johnston, R.J., and Hobert, O. (2005). A novel C. elegans zinc finger transcription factor, lsy-2, required for the cell type-specific expression of the lsy-6 microRNA. Development *132*, 5451–5460.

Johnston, R.J., Jr., and Hobert, O. (2003). A microRNA controlling left/right neuronal asymmetry in Caenorhabditis elegans. Nature *426*, 845–849.

Kamath, R.S., and Ahringer, J. (2003). Genome-wide RNAi screening in Caenorhabditis elegans. Methods *30*, 313–321.

Kaul, A., Schuster, E., and Jennings, B.H. (2014). The Groucho co-repressor is primarily recruited to local target sites in active chromatin to attenuate transcription. PLoS Genet. *10*, e1004595.

Kent, D.M., Rothwell, P.M., Ioannidis, J.P., Altman, D.G., and Hayward, R.A. (2010). Assessing and reporting heterogeneity in treatment effects in clinical trials: a proposal. Trials *11*, 85.

Konstantinides, N., Kapuralin, K., Fadil, C., Barboza, L., Satija, R., and Desplan, C. (2018). Phenotypic convergence: distinct transcription factors regulate common terminal features. Cell *174*, 622–635.e13.

Kueh, H.Y., Yui, M.A., Ng, K.K.H., Pease, S.S., Zhang, J.A., Damle, S.S., Freedman, G., Siu, S., Bernstein, I.D., Elowitz, M.B., and Rothenberg, E.V. (2016). Asynchronous combinatorial action of four regulatory factors activates Bcl11b for T cell commitment. Nat. Immunol. *17*, 956–965.

Kwon, G.S., Viotti, M., and Hadjantonakis, A.K. (2008). The endoderm of the mouse embryo arises by dynamic widespread intercalation of embryonic and extraembryonic lineages. Dev. Cell *15*, 509–520.

Langmead, B., and Salzberg, S.L. (2012). Fast gapped-read alignment with Bowtie 2. Nat. Methods *9*, 357–359.

Lawrence, M., Huber, W., Pagès, H., Aboyoun, P., Carlson, M., Gentleman, R., Morgan, M.T., and Carey, V.J. (2013). Software for computing and annotating genomic ranges. PLoS Comput. Biol. *9*, e1003118.

Lawrence, M.F., Gentleman, R., and Carey, V.J. (2009). rtracklayer: an R package for interfacing with genome browsers. Bioinformatics *25*, 1841–1842.

Lee, S., Lee, B., Joshi, K., Pfaff, S.L., Lee, J.W., and Lee, S.K. (2008). A regulatory network to segregate the identity of neuronal subtypes. Dev. Cell *14*, 877–889.

Leyva-Díaz, E., and Hobert, O. (2019). Transcription factor autoregulation is required for acquisition and maintenance of neuronal identity. Development *146*, dev177378.

Li, H., Handsaker, B., Wysoker, A., Fennell, T., Ruan, J., Homer, N., Marth, G., Abecasis, G., and Durbin, R.; 1000 Genome Project Data Processing Subgroup (2009). The Sequence Alignment/Map format and SAMtools. Bioinformatics *25*, 2078–2079.

Liao, Y., Smyth, G.K., and Shi, W. (2019). The R package Rsubread is easier, faster, cheaper and better for alignment and quantification of RNA sequencing reads. Nucleic Acids Res. *47*, e47.

Liu, X., Chen, W., Li, W., Li, Y., Priest, J.R., Zhou, B., Wang, J., and Zhou, Z. (2019). Single-cell RNA-seq of the developing cardiac outflow tract reveals convergent development of the vascular smooth muscle cells. Cell Rep. *28*, 1346–1361.e4.

Long, H.K., Prescott, S.L., and Wysocka, J. (2016). Ever-changing landscapes: transcriptional enhancers in development and evolution. Cell *167*, 1170–1187.

Machanick, P., and Bailey, T.L. (2011). MEME-ChIP: motif analysis of large DNA datasets. Bioinformatics *27*, 1696–1697.

Martin, M. (2011). Cutadapt removes adapter sequences from high-throughput sequencing reads. EMBnet J. *17*, 10–12.

Mello, C., and Fire, A. (1995). DNA transformation. Methods Cell Biol. *48*, 451–482.

Narasimhan, K., Lambert, S.A., Yang, A.W.H., Riddell, J., Mnaimneh, S., Zheng, H., Albu, M., Najafabadi, H.S., Reece-Hoyes, J.S., Fuxman Bass, J.I., et al. (2015). Mapping and analysis of Caenorhabditis elegans transcription factor sequence specificities. eLife *4*, e06967.

Ng, H.H., Robert, F., Young, R.A., and Struhl, K. (2003). Targeted recruitment of Set1 histone methylase by elongating Pol II provides a localized mark and memory of recent transcriptional activity. Mol. Cell *11*, 709–719.

Ng, K.K., Yui, M.A., Mehta, A., Siu, S., Irwin, B., Pease, S., Hirose, S., Elowitz, M.B., Rothenberg, E.V., and Kueh, H.Y. (2018). A stochastic epigenetic switch controls the dynamics of T-cell lineage commitment. eLife *7*, e37851.

Noma, K.-I., and Grewal, S.I.S. (2002). Histone H3 lysine 4 methylation is mediated by Set1 and promotes maintenance of active chromatin states in fission yeast. Proc. Natl. Acad. Sci. USA *99*, 16438–16445.

Okkema, P.G. (2017). The remarkably diverse family of T-box factors in Caenorhabditis elegans. Curr. Top. Dev. Biol. *122*, 27–54.

O'Meara, M.M., Zhang, F., and Hobert, O. (2010). Maintenance of neuronal laterality in Caenorhabditis elegans through MYST histone acetyltransferase complex components LSY-12, LSY-13 and LIN-49. Genetics *186*, 1497–1502.

Paix, A., Folkmann, A., Rasoloson, D., and Seydoux, G. (2015). High efficiency, homology-directed genome editing in Caenorhabditis elegans using CRISPR-Cas9 ribonucleoprotein complexes. Genetics *201*, 47–54.

Pereira, L., Kratsios, P.P., Serrano-Saiz, E., Sheftel, H., Mayo, A.E., Hall, D.H., White, J.G., LeBoeuf, B., Garcia, L.R., Alon, U., and Hobert, O. (2015). A cellular and regulatory map of the cholinergic nervous system of C. elegans. eLife *4*, e12432.

Pierce-Shimomura, J.T., Faumont, S., Gaston, M.R., Pearson, B.J., and Lockery, S.R. (2001). The homeobox gene lim-6 is required for distinct chemosensory representations in C. elegans. Nature *410*, 694–698.

Poole, R.J., Bashllari, E., Cochella, L., Flowers, E.B., and Hobert, O. (2011). A genome-wide RNAi screen for factors involved in neuronal specification in Caenorhabditis elegans. PLoS Genet. *7*, e1002109.

Poole, R.J., and Hobert, O. (2006). Early embryonic programming of neuronal left/right asymmetry in C. elegans. Curr. Biol. *16*, 2279–2292.

Rada-Iglesias, A., Bajpai, R., Swigut, T., Brugmann, S.A., Flynn, R.A., and Wysocka, J. (2011). A unique chromatin signature uncovers early developmental enhancers in humans. Nature *470*, 279–283.

Raj, A., van den Bogaard, P., Rifkin, S.A., van Oudenaarden, A., and Tyagi, S. (2008). Imaging individual mRNA molecules using multiple singly labeled probes. Nat. Methods *5*, 877–879.

Raj, B., Wagner, D.E., McKenna, A., Pandey, S., Klein, A.M., Shendure, J., Gagnon, J.A., and Schier, A.F. (2018). Simultaneous single-cell profiling of lineages and cell types in the vertebrate brain. Nat. Biotechnol. *36*, 442–450.

Reiter, F., Wienerroither, S., and Stark, A. (2017). Combinatorial function of transcription factors and cofactors. Curr. Opin. Genet. Dev. *43*, 73–81.

Rubin, A.J., Barajas, B.C., Furlan-Magaril, M., Lopez-Pajares, V., Mumbach, M.R., Howard, I., Kim, D.S., Boxer, L.D., Cairns, J., Spivakov, M., et al. (2017). Lineage-specific dynamic and pre-established enhancer-promoter contacts cooperate in terminal differentiation. Nat. Genet. *49*, 1522–1528.

Sarin, S., Antonio, C., Tursun, B., and Hobert, O. (2009). The C. elegans Tailless/TLX transcription factor nhr-67 controls neuronal identity and left/right asymmetric fate diversification. Development *136*, 2933–2944.

Sarin, S., O'Meara, M.M., Flowers, E.B., Antonio, C., Poole, R.J., Didiano, D., Johnston, R.J., Chang, S., Narula, S., and Hobert, O. (2007). Genetic screens for Caenorhabditis elegans mutants defective in left/right asymmetric neuronal fate specification. Genetics *176*, 2109–2130.

Sartorelli, V., and Puri, P.L. (2018). Shaping gene expression by landscaping chromatin architecture: lessons from a master. Mol. Cell *71*, 375–388.

Schnabel, R., Hutter, H., Moerman, D., and Schnabel, H. (1997). Assessing normal embryogenesis in Caenorhabditis elegans using a 4D microscope: variability of development and regional specification. Dev. Biol. *184*, 234–265.

Serrano-Saiz, E., Poole, R.J., Felton, T., Zhang, F., De La Cruz, E.D., and Hobert, O. (2013). Modular control of glutamatergic neuronal identity in C. elegans by distinct homeodomain proteins. Cell *155*, 659–673.

Shaner, N.C., Lambert, G.G., Chammas, A., Ni, Y., Cranfill, P.J., Baird, M.A., Sell, B.R., Allen, J.R., Day, R.N., Israelsson, M., et al. (2013). A bright monomeric green fluorescent protein derived from Branchiostoma lanceolatum. Nat. Methods *10*, 407–409.

Soufi, A., Garcia, M.F., Jaroszewicz, A., Osman, N., Pellegrini, M., and Zaret, K.S. (2015). Pioneer transcription factors target partial DNA motifs on nucleosomes to initiate reprogramming. Cell *161*, 555–568.

Spitz, F., and Furlong, E.E.M. (2012). Transcription factors: from enhancer binding to developmental control. Nat. Rev. Genet. *13*, 613–626.

Sulston, J.E., Schierenberg, E., White, J.G., and Thomson, J.N. (1983). The embryonic cell lineage of the nematode Caenorhabditis elegans. Dev. Biol. *100*, 64–119.

Suzuki, H., Thiele, T.R., Faumont, S., Ezcurra, M., Lockery, S.R., and Schafer, W.R. (2008). Functional asymmetry in Caenorhabditis elegans taste neurons and its computational role in chemotaxis. Nature *454*, 114–117.

Taverna, S.D., Ilin, S., Rogers, R.S., Tanny, J.C., Lavender, H., Li, H., Baker, L., Boyle, J., Blair, L.P., Chait, B.T., et al. (2006). Yng1 PHD finger binding to H3 trimethylated at K4 promotes NuA3 HAT activity at K14 of H3 and transcription at a subset of targeted ORFs. Mol. Cell *24*, 785–796.

Tursun, B., Cochella, L., Carrera, I., and Hobert, O. (2009). A toolkit and robust pipeline for the generation of fosmid-based reporter genes in C. elegans. PLoS One *4*, e4625.

Uchida, O., Nakano, H., Koga, M., and Ohshima, Y. (2003). The C. elegans che-1 gene encodes a zinc finger transcription factor required for specification of the ASE chemosensory neurons. Development *130*, 1215–1224.

Venkatesh, S., and Workman, J.L. (2015). Histone exchange, chromatin structure and the regulation of transcription. Nat. Rev. Mol. Cell Biol. *16*, 178–189.

Virtanen, P., Gommers, R., Oliphant, T.E., Haberland, M., Reddy, T., Cournapeau, D., Burovski, E., Peterson, P., Weckesser, W., Bright, J., et al. (2020). SciPy 1.0: fundamental algorithms for scientific computing in Python. Nat. Methods *17*, 261–272.

Wagner, D.E., Weinreb, C., Collins, Z.M., Briggs, J.A., Megason, S.G., and Klein, A.M. (2018). Single-cell mapping of gene expression landscapes and lineage in the zebrafish embryo. Science *360*, 981–987.

Wang, H., Liu, J., Gharib, S., Chai, C.M., Schwarz, E.M., Pokala, N., and Sternberg, P.W. (2017a). cGAL, a temperature-robust GAL4-UAS system for Caenorhabditis elegans. Nat. Methods *14*, 145–148.

Wang, S., Tang, N.H., Lara-Gonzalez, P., Zhao, Z., Cheerambathur, D.K., Prevo, B., Chisholm, A.D., Desai, A., and Oegema, K. (2017b). A toolkit for

GFP-mediated tissue-specific protein degradation in C. elegans. Development *144*, 2694–2701.

Wenick, A.S., and Hobert, O. (2004). Genomic cis-regulatory architecture and trans-acting regulators of a single interneuron-specific gene battery in C. elegans. Dev. Cell *6*, 757–770.

Xue, D., Tu, Y., and Chalfie, M. (1993). Cooperative interactions between the Caenorhabditis elegans homeoproteins UNC-86 and MEC-3. Science *261*, 1324–1328.

Yemini, E., Lin, A., Nejatbakhsh, A., Varol, E., Sun, R., Mena, G.E., Samuel, A.D.T., Paninski, L., Venkatachalam, V., and Hobert, O. (2019). NeuroPAL: a neuronal polychromatic atlas of landmarks for whole-brain imaging in C. elegans. bioRxiv. https://doi.org/10.1101/676312.

Yu, G., Wang, L.G., and He, Q.Y. (2015). ChIPseeker: an R/Bioconductor package for ChIP peak annotation, comparison and visualization. Bioinformatics *31*, 2382–2383.

Zaret, K.S., and Carroll, J.S. (2011). Pioneer transcription factors: establishing competence for gene expression. Genes Dev. *25*, 2227–2241.

Zaret, K.S., and Mango, S.E. (2016). Pioneer transcription factors, chromatin dynamics, and cell fate control. Curr. Opin. Genet. Dev. *37*, 76–81.

Zhang, F., Bhattacharya, A., Nelson, J.C., Abe, N., Gordon, P., Lloret-Fernandez, C., Maicas, M., Flames, N., Mann, R.S., Colón-Ramos, D.A., and Hobert, O. (2014). The LIM and POU homeobox genes ttx-3 and unc-86 act as terminal selectors in distinct cholinergic and serotonergic neuron types. Development *141*, 422–435.

Zhang, Y., Liu, T., Meyer, C.A., Eeckhoute, J., Johnson, D.S., Bernstein, B.E., Nusbaum, C., Myers, R.M., Brown, M., Li, W., and Liu, X.S. (2008). Model-based analysis of ChIP-seq (MACS). Genome Biol. *9*, R137.

Zhu, F., Farnung, L., Kaasinen, E., Sahu, B., Yin, Y., Wei, B., Dodonova, S.O., Nitta, K.R., Morgunova, E., Taipale, M., et al. (2018). The interaction landscape between transcription factors and the nucleosome. Nature *562*, 76–81.

STAR★METHODS

KEY RESOURCES TABLE

REAGENT or RESOURCE	SOURCE	IDENTIFIER
Antibodies		
Rabbit polyclonal anti-GFP	Abcam	Cat# ab290; RRID: AB_303395
Bacterial and Virus Strains		
Escherichia coli: HB101 Strain	CGC	HB101
Escherichia coli: HT115 Strain	CGC	HT115
Escherichia coli: OP50 Strain	CGC	OP50
Chemicals, Peptides, and Recombinant Proteins		
Chitinase	Sigma	Cat# C6137
cOmplete™, Mini Protease Inhibitor Cocktail	Roche	Cat# 11836153001
Dextran sulfate sodium salt	Sigma	Cat# D6001
EvaGreen® Dye	Biotium	Cat# 31000
Formaldehyde solution	Sigma	Cat# F8775
Formamide (Deionized)	Ambion	Cat# AM9342
Leibovitz;s L-15 Medium, no phenol red	Gibco	Cat# 21083027
Penicillin-Streptomycin	Sigma	Cat# P4458
ProLong™ Gold Antifade reagent with DAPI	Invitrogen	Cat# P36935
Pronase	Sigma	Cat# P6911
SYBR™ Green I Nucleic Acid Gel Stain	ThermoFisher	Cat# S7563
SYTOX™ AADvanced Dead Cell Stain Kit	Invitrogen	Cat# S10349
Critical Commercial Assays		
AMPure XP Beads	Beckman Coulter	Cat# A63882
DNA Clean & Concentrator Kit-5	Zymo Research	Cat# D4013
Dynabeads™ Protein A Immunoprecipitation Kit	ThermoFisher	Cat# 100006D
NEBNext® High Fidelity 2x PCR Master Mix	New England BioLabs	Cat# M0541L
NEBNext® Ultra II DNA Library Prep Kit for Illumina®	New England BioLabs	Cat# E7645S
Nextera DNA Library Prep Kit	Illumina	Cat# 15028212
Nextera i7 and i5 adapters	Illumina	Cat# 20027213
Protein Assay Dye Reagent Concentrate	Bio-Rad	Cat# 5000006
Quasar 670-conjugated RNA FISH Probe Sets	Biosearch Technologies	N/A
Deposited Data		
ATAC-seq & ChIP-seq data	This Study	GEO: GSE155392
Experimental Models: Organisms/Strains		
Please see Table S4 for a complete list of strains used in this study		
Oligonucleotides		
crRNA targeting *che-1* #1: TAAAGAGGGTGGAGCTTCAG	IDT	N/A
crRNA targeting *che-1* #2: CACAGAGTGGGAACTTGCAT	IDT	N/A
crRNA targeting *lsy-6* #1: ATGAGACGCATTTCGATGAC	IDT	N/A
crRNA targeting *tbx-37* #1: GCTCAAAATTACAATAATTT	IDT	N/A
crRNA targeting *tbx-37* #2: GAGCAGCAAACTGTGGCTGG	IDT	N/A

(*Continued on next page*)

Continued

REAGENT or RESOURCE	SOURCE	IDENTIFIER
crRNA targeting *tbx-38* #1: ATTGTACTGCACTGATCTCC	IDT	N/A
crRNA targeting *tbx-38* #2: TACCTATTGCCTTTCTCCCC	IDT	N/A
crRNA targeting *tbx-11* #1: AACAGGAAAAATACACCCGG	IDT	N/A
crRNA targeting *tbx-11* #2: CCGCCCAACACGTGGCAAGG	IDT	N/A
crRNA targeting *tbx-43* #1: ATTTTTCCATAAGCGCCACG	IDT	N/A
crRNA targeting *tbx-43* #2: GGCCGAGAACTCCTCCAACG	IDT	N/A
Recombinant DNA		
Please see Table S5 for a complete list of Recombinant DNA used in this study		
Software and Algorithms		
bowtie2 (v2.2.4)	Langmead and Salzberg, 2012	N/A
ChIPseeker (v1.22.1)	Yu et al., 2015	N/A
Cutadapt (v1.18)	Martin, 2011	N/A
GenomicRanges (v1.38.0)	Lawrence et al., 2013	N/A
ImageJ (Fiji)	NIH	N/A
kent-uscs (v2.79)	Kent et al., 2010	N/A
macs2 (v2.1.0)	Zhang et al., 2008	N/A
MEME Suite (v5.1.1)	Machanick & Bailey, 2011	N/A
MetaMorph	Molecular Devices	N/A
rtracklayer (v1.46.0)	Lawrence et al., 2009	N/A
Picard-tools (v2.18.27)	Broad Institute	N/A
Prism 7	GraphPad Prism	N/A
Rsubread (v2.0.1)	Liao et al., 2019	N/A
SAMtools (v0.1.18)	Li et al., 2009	N/A
SciPy	Virtanen et al., 2020	N/A
SIMI BioCell	Schnabel et al., 1997	N/A
Time to Live	Caenotec	N/A
Zen	Zeiss	N/A
Other		
Axio Imager.Z2	Zeiss	N/A
Axio Observer Confocal with Airyscan	Zeiss	Cat# LSM880
Bioruptor® Plus sonication device	Diagenode	Cat# B01020001
HiSeq 2500 Sequencing System	Illumina	N/A
NanoDrop™ 3300 Fluorospectrometer	ThermoFisher	Cat# ND-3300
pluriStrainer® 5 µm cell strainer	pluriSelect	Cat# 43-10005-40
SH800S Cell Sorter	Sony	N/A
Sorting Chip 100 µm	Sony	Cat# LE-C3210
Visiscope Spinning Disc Confocal	Visitron Systems GmbH	N/A

RESOURCE AVAILABILITY

Lead Contact

Further information and requests concerning resources and reagents should be directly addressed to Luisa Cochella (cochella@imp.ac.at).

Materials Availability

All *C. elegans* strains generated in this study will be made available through the CGC. Plasmid and fosmid-based reporters generated in this study are available from the corresponding author upon request.

Data and Code Availability

All datasets (ChIP-seq and ATAC-seq) generated in this study are available under GEO accession number GSE155392. This study did not generate code.

EXPERIMENTAL MODEL AND SUBJECT DETAILS

Strains

All *Caenorhabditis elegans* strains were maintained on nematode growth media (NGM) plates seeded with OP50 bacteria (Brenner, 1974), at 20°C unless otherwise indicated. A full list of strains used in this study can be found in Table S4.

METHOD DETAILS

Strain Generation

Standard microinjection procedures were used to generate transgenic worms with extra chromosomal arrays (Mello and Fire, 1995). Briefly, DNA was injected as complex arrays in the gonads of young adults. Injection mixes contained 1-5 ng/μL of the plasmid of interest and a similar amount of a co-injection marker (typically *che-1*^{prom}*::mCherry, ttx-3*^{prom}*::mCherry* or *unc-122*^{prom}*::mCherry*), in addition to 100 ng/μL of sonicated genomic DNA from *E. coli* OP50. Some extrachromosomal arrays were then integrated into the genome by random integration stimulated by gamma irradiation (14 minutes; 4000 rads). All integrants were outcrossed 3-6 times with the wild type strain N2.

CRISPR-Cas9 Directed Homology Repair

Homology-directed genome editing using *in vitro* assembled Cas9, CRISPR RNA (crRNA) and trans-activating crRNA (tracrRNA) ribonucleoprotein complexes was performed as previously described (Paix et al., 2015). Briefly, purified Cas9 and synthetic RNAs were pre-incubated to enable complex formation and injected into the gonad of young adults. Alt-R® CRISPR-Cas9 crRNA targeting the DNA sequences available in the Key Resources Table were obtained from Integrated DNA Technologies (IDT). *che-1(luc174)* was created by deleting 3,384 bp of the *che-1* locus, including most of its coding sequence. *lsy-6(luc157)* was created by replacing the *lsy-6* hairpin sequence with the *yfp* sequence. *lsy-6(luc160)* was created by deleting 150 bp downstream of *lsy-6*, overlapping with the TBX-37/38 binding sites. *lsy-6(luc156)* was created by replacing the *lsy-6* hairpin sequence with *yfp* and simultaneously introducing the same 150 bp deletion downstream. *tbx-37(luc41)* and *tbx-38(luc54)* were created by insertion of a *gfp* with a flexible linker (4x GGGGS) at the N terminus of *tbx-37* or *tbx-38*. *tbx-11(luc144)* was created by deleting 1303 bp of the *tbx-11* locus, including the whole coding sequence. *tbx-43(luc131)* was created by deleting 952 bp of the *tbx-43* promoter region and part of the first exon.

Mos1-Mediated Single-Copy Insertions (mosSCI)

The published procedure (Frøkjaer-Jensen et al., 2012) was used to generate transgenic worms with targeted single-copy insertions of the *hsp-16.41p::vhhGFP4::zif-1::SL2::mCherry::his-11::tbb-2 3'UTR* transgene (*lucSi100*) or *hsp-16.41p::zif-1::SL2::mCherry::his-11::tbb-2 3'UTR* (*lucSi102*) transgene at the Chr II landing site *ttTi5605* (www.wormbuilder.org). Briefly, a defined Mos1 transposon insertion in Chr II (in an *unc-119* mutant background which is paralyzed) is mobilized by transgenic expression of the transposase and the desired insert is provided flanked by homology arms to the excision site to promote homology-directed repair. The inserted cassette also contains a rescuing *unc-119* minigene and thus selection of animals with the desired insertion is done based on rescue of the paralysis phenotype. Counterselection for the formation of extrachromosomal arrays is performed using heat-shock inducible *peel-1* toxin.

Fosmid Recombineering

Fosmid-based reporters have been generated as previously described (Tursun et al., 2009). Fosmid clones carrying 35-45 kb of genomic sequence (containing the gene of interest) in the pCC1Fos backbone were transformed into SW105 *E. coli*. A GFP or T2A::GFP::H2B cassette containing a galK minigene for positive selection was amplified while adding 50 bp of homology to the desired insertion site. The linear PCR products were transformed into SW105 *E. coli* carrying the fosmid of interest and induced for expression of Red recombinase. Bacteria carrying the desired insertion were selected on minimal medium with galactose as sole carbon source. Positive clones were further isolated according to (Tursun et al., 2009) and verified by Sanger sequencing. Primer sequences used to generate all fosmid constructs in this work, as well as sequences, are available upon request. A full list of fosmid-based reporters used in this study can be found in Table S5.

Plasmid Construction

All constructs were generated by standard molecular cloning procedures with restriction digest, PCR, and Gibson assembly or T4 ligation. The coding sequences in the constructs were verified by Sanger sequencing. Plasmid maps and sequences are available upon request. A full list of plasmids used in this study can be found in the Table S5.

CellPress
OPEN ACCESS

Chromatin Immunoprecipitation Sequencing (ChIP-Seq)

ChIP-seq experiments were performed as previously described in (Askjaer et al., 2014) with minor modifications as described below. We performed two biological replicates for TBX-37 and one for TBX-38.

Worm Synchronization and Eggs Extraction

N2, MLC813 (endogenous GFP-TBX-37 and *tbx-38* deleted) and MLC893 (endogenous GFP-TBX-38 and *tbx-37* deleted) worms were cultivated on at least 10 x 10 cm peptone enriched plates seeded with concentrated HB101 *E. coli* bacteria. The resulting adult worms were bleached using hypochlorite solution (1% NaOCl; 1 M NaOH) and isolated eggs were washed and incubated overnight in M9 buffer (22 mM KH_2PO_4; 42 mM Na_2HPO_4; 86 mM NaCl; 1 mM $MgSO_4$) at 20°C under agitation. Up to 90000 synchronized L1 larvae were incubated at 18°C for ~71 h on 16 cm peptone enriched plates with concentrated HB101 to obtain gravid adult worms about to lay their first eggs. Worms were collected, washed 3 times with M9 buffer, and bleached with hypochlorite solution to release synchronized eggs at 1–2-cell stage. These embryos were allowed to develop for 90 min at 20°C and then used for the ChIP assay.

Chromatin Immunoprecipitation

Eggs were washed 3 times with M9 and resuspended in 45 mL of M9 and fixed with 2% formaldehyde solution [Sigma; cat #F8775] for 30 min at room temperature under agitation. Fixation was stopped by adding glycine to 125 mM final concentration for 5 min at room temperature, under agitation. Fixed embryos were then washed 2 times with M9 buffer, resuspended in 1 mL PBS, 1 mM PMSF and protease inhibitor solution [Roche; cat #11836153001], transferred to microfuge tube and pelleted at 6000 g for 1 min at room temperature and washed one more time in 1 mL PBS, 1 mM PMSF with protease inhibitor solution. About 100 μL of packed embryos were washed once with 1 mL of FA buffer (50 mM HEPES/KOH pH 7,5; 1 mM EDTA; 1% Triton™ X-100; 0,1% sodium deoxycholate; 150 mM NaCl; 1mM PMSF; 0,1% sarkosyl and protease inhibitor). Embryos were dounced on ice for at least 30 strokes using a glass dounce homogenizer pestle type B. Resulting embryos were transferred to polystyrene 15 mL tubes and sonicated at 4°C for 15 min using a Bioruptor® [Diagenode; cat #B01020001] with the following settings: High, 30 seconds ON, 30 seconds OFF. Chromatin was cleared by centrifugation at top speed (13000-17000 g) for 15 min at 4°C. Cleared chromatin was transferred into a new microcentrifuge tube and 25 μL of the sample was saved to determine quality of DNA shearing. Protein concentration of the cleared chromatin was then determined using Bradford assay [Bio-rad; cat #5000006]; 4 mg of total chromatin was used for each assay. The reaction volume was brought up to 1 mL in FA buffer + 1 mM PMSF, 0,1% sarkosyl and protease inhibitor. 5% of the reaction volume was saved as Input fraction. 1 μg of Anti-GFP antibody [Abcam; cat #ab290] was added to the reaction and incubated at 4°C overnight under rotation. The next day 40 μL of magnetic protein A beads [Thermofisher; cat #10006D] equilibrated with FA buffer were added to the reaction for 2h at 4°C. Beads were subsequently washed 2 times with FA buffer for 5 min, 1 time with FA Buffer + 1 M NaCl for 5 min, transferred to a new microfuge tube and washed with FA-500 mM NaCl (50 mM HEPES/KOH pH 7,5; 1 mM EDTA; 1% Triton X-100; 0.1% sodium deoxycholate; 500 mM NaCl) for 10 min, 1 time with TEL buffer (0.25 M LiCl, 1% NP-40, 1% sodium deoxycholate, 1 mM EDTA, 10 mM Tris-HCl, pH 8.0) for 10 min and finally 2 times with TE for 5 min (10 mM Tris-HCl, 1 mM EDTA).

To elute, 125 μL of ChIP elution buffer (1% SDS; 250 mM NaCl; 10 mM Tris pH 8,0; 1 mM EDTA) was added to the beads and incubated at 65°C for 15 min with vortexing every 5 min. Beads were precipitated at 6000 g for 1 min, the supernatant was kept in a new microfuge tube and elution was repeated a second time before pooling the supernatants together. Input samples were eluted by adding 200 μL of ChIP elution buffer + 2 μg proteinase K and incubated for 1h at 50°C. Samples were reverse-crosslinked by incubation at 65°C overnight. The resulting DNA was purified using DNA Clean and Concentration kit [Zymo research; ref #D4013]. ChIP DNA was eluted in 32 μL and Input DNA in 50 μL.

Library Preparation

DNA concentration was assessed using SYBR™ Green I [ThermoFisher; cat #S7563] and a Fluorospectrophotometer [ThermoFisher; cat #ND-3300]. Libraries were prepared using NEBNext® Ultra™ II DNA Library Prep Kit [New England Biolabs; cat #E7645S] according to the instructions of the manufacturer. The ChIP-seq libraries were sequenced on Hiseq 2500 Sequencing System (Illumina) with single-end 50 nt parameter.

ChIP-seq Data Processing and Analysis

The single-end raw reads of ChIP-seq dataset were mapped to the *C. elegans* genome (ce11) with bowtie2 (v2.2.4) (Langmead and Salzberg, 2012). Uniquely mapped reads were retained and duplicate reads were marked and discarded using SAMtools (v0.1.18) (Li et al., 2009). For visualization, the coverage tracks (i.e. bigWig files) were generated from bam files using kent-ucsc (v2.79) (Kent et al., 2010). Shaped-base identification was used to call the peaks with MACS2 (v2.1.0) against the input sample with default parameters (Zhang et al., 2008). The identified peaks conserved in two out of three TBX-37 and TBX-38 ChIP-seq replicates were retained. *De novo* motif discovery was performed using the MEME Suite (v5.1.1) (Machanick and Bailey, 2011). ChIP-seq datasets were deposited in GEO under accession number GSE155392.

Genetically Induced Ectopic TBX-37 Expression

MLC2203 strain, carrying the endogenously tagged *lsy-6::yfp* allele, a red fluorescent ASE marker (*che-1^{prom}::mCherry*) and an extrachromosomal transgene for expression of the *tbx-37* cDNA under a heat-shock promoter, was grown at 20°C prior to heat-shock experiments. 2-cells embryos were picked with a capillary mouth pipette, following dissection of gravid mothers, and transferred to a plate without food. Embryos were allowed to develop at 20°C for 90 min (first time point – 8 ABa; TBX-37/38 peak of expression),

120 min (16 ABa), 150 min (32 ABa), 180 min (64 ABa), 210 min (128 ABa), 240 min, 270 min or 300 min (final time point – bean stage; prior the birth of the ASEs). The embryos were then heat shocked for 20 min at 30°C to induce ectopic TBX-37 expression and put back at 20°C to continue development. Embryos were mounted after 400 min on a 5% agarose pad on a glass slide and examined for YFP expression in both ASEs using a Zeiss Axio Imager.Z2 with sCMOS camera, Sola SM2 solid state white light excitation system and 63x/1.4 plan-apochromat Oil DIC objective.

Single Molecule Fluorescence *In Situ* Hybridisation (smFISH)

smFISH was performed as previously described in (Raj et al., 2008) with minor modifications as described in detail below. Probe sets targeting the GFP transcript (GFP sense) and its reverse complement (GFP antisense) were designed using the Stellaris RNA FISH probe designer. The purified Quasar 670-conjugated probes were obtained from Biosearch Technologies. The sequences used for the probe sets design are available here:

GFP Sense

atgagtaaaggagaagaacttttcactggagttgtcccaattcttgttgaattagatggtgatgttaatgggcacaaattttctgtcagtggagagggtgaaggtgatgcaacatacgga
aaacttacccttaaatttatttgcactactggaaaactacctgttccatggccaacacttgtcactactttctgttatggtgttcaatgcttctcgagatacccagatcatatgaaacggca
tgactttttcaagagtgccatgcccgaaggttatgtacaggaaagaactatatttttcaaagatgacgggaactacaagacacgtgctgaagtcaagtttgaaggtgatacccttgtta
atagaatcgagttaaaaggtattgattttaaagaagatggaaacattcttggacacaaattggaatacaactataactcacacaatgtatacatcatggcagacaaacaaaagaatg
gaatcaaagttaacttcaaaattagacacaacattgaagatggaagcgttcaactagcagaccattatcaacaaaatactccaattggcgatggccctgtccttttaccagacaacc
attacctgtccacacaatctgccctttcgaaagatcccaacgaaaagagagaccacatggtccttcttgagtttgtaacagctgctgggattacacatggcatggatgaactatacaaa

GFP Antisense

tttgtatagttcatccatgccatgtgtaatcccagcagctgttacaaactcaagaaggaccatgtggtctctcttttcgttgggatctttcgaaagggcagattgtgtggacaggtaatgg
ttgtctggtaaaaggacagggccatcgccaattggagtattttgttgataatggtctgctagttgaacgcttccatcttcaatgttgtgtctaattttgaagttaactttgattccattcttttgtt
tgtctgccatgatgtatacattgtgtgagttatagttgtattccaatttgtgtccaagaatgtttccatcttctttaaaatcaataccttttaactcgattctattaacaagggtatcaccttcaaa
cttgacttcagcacgtgtcttgtagttcccgtcatctttgaaaaatatagttctttcctgtacataaccttcgggcatggcactcttgaaaaagtcatgccgtttcatatgatctgggtatctc
gagaagcattgaacaccataacagaaagtagtgacaagtgttggccatggaacaggtagttttccagtagtgcaaataaatttaagggtaagttttccgtatgttgcatcaccttcacc
ctctccactgacagaaaatttgtgcccattaacatcaccatctaattcaacaagaattgggacaactccagtgaaaagttcttctcctttactcat

Egg Extraction and Fixation

MLC813 [tbx-37(luc41[GFP::flex::TBX-37]) tbx-38(tm581) III], MLC893 [tbx-37(tm314) tbx-38(luc54[GFP::flex::TBX-38]) III], OH8993 [otIs252[lsy-6::YFP (fosmid); rol-6(su1006)] II], OH11115 [otIs386[lsy-6::GFP::Δ150 (fosmid); ttx-3p::mCherry]] and MLC954 [otIs252 II; tbx-37(tm314) tbx-38(tm581) III/qC1[dpy-19(e1259) glp-1(q339) qIs26[lag-2::GFP; rol-6(su1006)]] worms were cultivated on 5 x 10 cm peptone enriched plates seeded with concentrated HB101 E. coli bacteria. Adult worms were bleached using hypochlorite solution and isolated eggs were washed three times in M9 buffer. Eggs were resuspended in 1 mL of fixation buffer (3,7% formaldehyde; 1X PBS), incubated 15 minu at room temperature, vortexed and then immediately submerged in liquid nitrogen for 1 min to freeze crack the eggshell. Eggs were then thawed at room temperature in water, vortexed and fixed on ice for an additional 20 min. Eggs were washed twice in 1 mL of 1X PBS, resuspended in 1 mL of 70% ethanol and permeabilized at 4°C for 48 h.

Hybridization

Following permeabilization, the eggs were washed in 1 mL of Wash Buffer (2X SSC; 5% formamide [Ambion; cat #AM9342]), incubated at room temperature for 5 min before resuspension in 100 μL of Hybridization Buffer (2X SSC; 10% formamide; 10% dextran sulfate [Sigma; cat #D6001]) containing 100 nM of fluorescently labeled RNA probes. Samples were incubated overnight at 37°C, in the dark. The next day, the samples were washed twice in 1 mL of Wash Buffer for 30 min each time (in the dark). The Wash Buffer was then removed and ProLong™ Gold Antifade reagent with DAPI [Invitrogen; cat #P36935] added to the samples.

Genetically Induced Protein Degradation

MLC1219 [lucSi102[hsp-16.41p::zif-1::SL2::mCherry::his-11::tbb-2 3'UTR] otIs252[lsy-6::YFP (fosmid); rol-6(su1006)] II; tbx-37(luc41[GFP::flex::tbx-37]) tbx-38(tm581) III], MLC1300 [lucSi100[hsp-16.41p::vhhGFP4::zif-1::SL2::mCherry::his-11::tbb-2 3'UTR] otIs252 II; tbx-37(luc41) tbx-38(tm581) III], MLC1466 [lucSi100 II; wgIs37[pha-4::TY1::EGFP::3xFLAG; unc-119]] and MLC1467 [lucSi102 II; wgIs37] strains were grown at 25°C prior to heat-shock experiments. 1- or 2-cells embryos were picked with a mouth pipette, following dissection of gravid mothers, and transferred on a plate without food, at 25°C, for 20 min (first time point (t_1) – TBX-37/38 expression peak) or 60 min (second time point (t_2) – following TBX-37/38 peak of expression). The embryos were heat-shocked at 30°C for 30 min to induce zif-1 or vhhGFP4::zif-1 expression. The plates were kept at 20°C for 8h and embryos were scored for lsy-6::YFP expression in ASEL, as well as for the tbx-37/38(0) phenotype, using a Zeiss Axio Imager.Z2 with sCMOS camera, Sola SM2 solid state white light excitation system and 40x/1.3 EC plan-neofluar Oil DIC objective.

Assay for Transposase-Accessible Chromatin Using Sequencing (ATAC-seq)

ATAC-seq libraries were prepared as described in (Corces et al., 2017) with minor modifications as described below:

Strain Design

To enable the sorting of cells from the ABa and ABp lineage, we generated an integrated array (*lucIs39*) which allows for the labeling and fluorescence-activated cell sorting (FACS) of the ABa descendants using *tbx-37^{prom}::mNeon* (Hostettler et al., 2017; Shaner et al., 2013), the EMS lineage by *med-2^{prom}::mScarlet-I* and the C and D lineages by *pal-1^{prom}::mScarlet-I* (Bindels et al., 2017).

The choice of promoters used for the design of *lucIs398* is based on previously reported time-resolved transcriptomic data (Boeck et al., 2016). This leaves cells from the ABp lineage, as well cells from the P lineage (Z2, Z3), unlabeled.

Worm Synchronization and Egg Extraction

MLC1480 [lucIs39[tbx-37p::mNeonGreen::2xNLS::tbx-37 3'UTR; pal-1p::mScarlet-I::2xNLS::tbb-2 3'UTR; med-2p::mScarlet-I::2xNLS::tbb-2 3'UTR]], MLC2239 [otIs252[lsy-6::YFP (fosmid); rol-6(su1006)] II; otIs235[che-1p::mChopti; rol-6(su1006)] V], MLC2309 [lucIs39; lsy-6(luc160[lsy-6::d150]) V] and MLC2310 [otIs232[che-1p::mChopti; rol-6] II; lsy-6(luc160) V] strains were synchronized through two cycles of bleaching as described above. On the first round, worms were cultivated on 150 mm peptone enriched plate seeded with concentrated HB101 E. coli bacteria. Adult worms were bleached using hypochlorite solution and isolated eggs were washed three times in M9 buffer and hatched for 14 h in M9 buffer at 20°C, with gentle agitation. The next day, 75000 starved L1s were plated on 150 mm peptone enriched plates with concentrated HB101 and incubated at 21°C for ~52h. As the worms reached the stage of young adults, the population was closely monitored and worms were collected when the first 2-4 eggs could be observed in the gonads of ~30% of the population. Collected worms were washed with ice-cold M9 buffer to remove residual bacteria and bleached with hypochlorite solution to release the eggs used for the ATAC-seq. Collected embryos were washed in egg buffer (25 mM HEPES pH 7,3; 118 mM NaCl; 48 mM KCl; 48 mM KCl; 2 mM $CaCl_2$; 2 mM $MgCl_2$) to remove residual M9 buffer and incubated at 21°C for 90, 200 and 350 min (ABa/ABp experiments) or 350 min (ASEL/R experiment). The developmental stages of embryos were monitored using a Zeiss Axio Imager.Z2 with sCMOS camera, Sola SM2 solid state white light excitation system and 40x/1.3 EC plan-neofluar Oil DIC objective.

Embryo Dissociation and Cell Sorting

The embryo suspension was concentrated to 0,5 mL by centrifugation at 1200 rcf for 1minute. To dissociate the eggshells, 0,5 mL of 2 mg/mL chitinase solution [Sigma; cat #C6137] was added to the embryo suspension and incubated for 25-30 min on ice with periodical shaking. 100 μL of 15 mg/mL pronase solution [Sigma; cat #P6911] was then added to the sample and a 2,5 mL syringe fitted with a 21 g needle was used to dissociate the embryos. The suspension was repeatedly passed through the needle until ~80% of embryos were dissociated. The digestion reaction was stopped by adding complete L-15 medium (L-15 no phenol red [Gibco; cat #21083027]; 10% FBS; 50 U/mL penicillin; 50 μg/mL streptomycin [Sigma; cat #P4458]) and cell suspension was filtered on 5 μm cell strainer [pluriStrainer; cat #43-10005-40] to remove undissociated embryos or cell aggregates. To label the dead cells, SYTOX AADvanced [Invitrogen; cat #S10349] was added to the sample to a final concentration of 1 μL/mL and the sample was incubated for 5 min, protected from light, and Fluorescent Activated Cell Sorting (FACS) was performed on a Sony SH800, equipped with 488 and 561 nm lasers. Sorting was performed using 100 μm microfluidic chips [Sony, cat #LE-C3210] with standard settings.

Library Preparation

For ATAC-seq library preparation, 15000-25000 cells (for isolated ABa/ABp descendants) or 1000-2500 cells (for sorted ASEL/ASER) were sorted in 300 μl of complete L-15 media and resuspended in cytosol extraction buffer (15 mM Tris-HCl pH 7,5; 5 mM $MgCl_2$; 60 mM KCl; 0,5 mM DTT; 15 mM NaCl; 30 mM sucrose and 1% IGEPAL CA-630). Nuclei were precipitated by centrifugation and washed in ATAC-Resuspension Buffer (10 mM Tris-HCl pH 7,4; 10 mM NaCl, 3 mM $MgCl_2$). DNA was tagmented using the Nextera DNA Library Prep Kit [Illumina; cat #15028212] and purified using the DNA Clean & Concentrator-5 [Zymo Research; cat #D4004]. The libraries were amplified using the NEBNext High Fidelity 2x PCR Master Mix [New England Biolabs; cat #M0541L] and Nextera i7 and i5 adapters [Nextera; cat #20027213] using the following thermocycling protocol: 72°C for 5 min, 98°C for 30 seconds, and several cycles of 98°C for 10 seconds, 63°C for 30 seconds and 1 min at 72°C. The number of PCR cycles required for amplification of individual libraries was determined empirically by real-time monitoring of the amplification profiles of the samples via their relative fluorescence units (RFUs) using EvaGreen [Biotium; cat #31000]. Typically, 10-18 cycles were needed. Libraries were cleaned-up using AMPure XP Beads [Beckman Coulter; cat #A63882].

Sequencing

Sequencing was performed by the Next Generation Sequencing Facility (VBCF) on a HiSeq 2500 system (Illumina) generating paired-end 50 bp reads. Barcoded libraries were pooled based on their measured concentration and sequenced on one lane (~ 20-40 million reads per sample). Two biological replicates were obtained for each time point.

Data Processing and Analysis

The paired-end raw reads of ATAC-seq data were trimmed with Cutadapt (v1.18) (Martin, 2011) and mapped to the *C. elegans* genome (ce11) with bowtie2 (v2.2.4) (Langmead and Salzberg, 2012). For all samples, multi-mapped reads were filtered with SAMtools (v0.1.20) (Li et al., 2009) and duplicate reads were marked and discarded with Picard-tools (v2.18.27; Broad Institute). For visualization, the normalized coverage tracks (i.e. bigWig files) were generated from bam files with the R packages GenomicRanges (v1.38.0) (Lawrence et al., 2013) and rtracklayer (v1.46.0) with paired-end option (Lawrence et al., 2009). Shape-based identification was used to call the peaks with MACS2 (v2.1.0) with default parameters (Zhang et al., 2008). The read counts for the pooled peaks were quantified using the Rsubread (v2.0.1) package (Liao et al., 2019) and the read counts per million (cpm) values were calculated as normalized peak signal. The R package ChIPseeker (v1.22.1) was used to assign the peaks to the closest genes (Yu et al., 2015). *De novo* motif discovery was performed using the MEME Suite (v5.1.1) (Machanick and Bailey, 2011) to uncover motifs specific to the ABa lineage (using the sequences under the peaks detected in the ABa lineage, non-overlapping with peaks from the ABp lineage at 90 min), ASEL-specific motifs (using ASEL peaks non-overlapping with ABa lineage peaks at 350 min) and ASER-specific motifs (using ASER peaks non-overlapping with ABp lineage peaks at 350 min).

ATAC-seq datasets were deposited in GEO under accession number GSE155392.

Heterologous Gal4-UAS Tethering Assays

Strain Design

To test the requirement for transcriptional activation in *lsy-6* priming, we generated reporters in which the 150 bp region containing the TBX-37/38 binding sites were replaced by five repeats of the Upstream Activation Sequence (UAS) (*lsy-6::gfp::Δtbs::5xUAS* reporter), or in which the 5xUAS repeat was inserted downstream of the 150 bp element (*lsy-6::gfp::5xUAS*), in the context of the *lsy-6::GFP* fosmid. We then used the tbx-37 promoter to dive expression of the *S. kudriavzevii* GAL4 DNA binding domain alone ($GAL4^{DBD}$), fused to four repeats of the VP16 transcriptional activation domain ($GAL4^{DBD}$-VP64) or fused to the transcriptional repressor Groucho/UNC-37 ($GAL4^{DBD}$-UNC-37) (Beerli et al., 1998; Chambers et al., 2017; Kaul et al., 2014; Wang et al., 2017a). The $GAL4^{DBD}$-VP64 fusion was also cloned downstream of a heat-shock promoter for the time-controlled activation experiments.

$GAL4^{DBD}$-VP64 Heat-Shock Time-Course

MLC2242 [*syIs400[hsp-16.42p::2xNLS::GAL4SK::VP64::let-858 3'UTR; unc-122p::RFP] V*; *luIs42[lsy-6::GFP::d150::5xUAS; ttx-3p::mCherry]*] animals were grown at 20°C prior to heat-shock experiments. 2-cells embryos were collected following dissection of gravid mothers, using a mouth pipette, and transferred on a plate without food. Embryos were allowed to develop at 20°C for 90 min (first time point – 8 ABa; TBX-37/38 peak of expression), 120 min (16 ABa), 150 min (32 ABa), 180 min (64 ABa), 210 min (128 ABa), 240 min, 270 min or 300 min (final time point – bean stage; prior the birth of the ASEs). The embryos were then heat-shocked for 30 min at 30°C to induce $GAL4^{DBD}$-VP64 expression and put back at 20°C to continue development. The following day, L1 animals were mounted on a glass slide covered with a 5% agarose pad and examined for GFP expression in ASEs using a Zeiss Axio Imager.Z2 with sCMOS camera, Sola SM2 solid state white light excitation system and 40x/1.3 EC plan-neofluar Oil DIC objective.

RNA Interference (RNAi)

RNA interference assays were performed by feeding worms with bacteria expressing dsRNA as described in (Conte et al., 2015). All feeding RNAi experiments with OH8996 were performed at 20° C. *E. coli* HT115 clones expressing either dsRNA of target transcripts or empty vector control (pL4440) were fed to L4 animals on NGM agar plates containing 25 μg/mL carbenicillin and 1 mM IPTG. Progenies were scored 3 days later for loss of *lsy-6* using a Zeiss Axio Imager.Z2 with sCMOS camera with 40x objective by assessing *gcy-5p::GFP* expression in 1 or 2 ASEs. The majority of the bacterial clones used in the study were obtained for the Ahringer library (Kamath and Ahringer, 2003). A full list of dsRNA expressing clones used in this study is available in the Key Resources Table.

Differential Interference Contrast and Fluorescence Microscopy

A Zeiss Axio Imager.Z2 fitted for DIC optics and with motorized stage, with sCMOS camera and Sola SM2 solid state white light excitation system was used for all scoring and acquisition of single timepoint z-stacks. Images were acquired using ZEN software from Zeiss.

4D Microscropy and Lineage Analysis

2-cell embryos were mounted in water, on microscopy slides with a thin 5% bacto-agar pad. Time-lapsed images were acquired using a Zeiss Axioplan 2 with Normaski/DIC and fluorescence optics. A LED emitting at 470 nm was used for GFP excitation and images were collected with Time to Live software (Caenotec). Lineage analysis was aided by SIMI BioCell software (Schnabel et al., 1997). Blastomere identities, positions, and divisions were followed through embryogenesis to precisely assess spatial and temporal expression onset of GFP reporters.

Confocal Microscopy

Neuropal

NeuroPal images were acquired and analyzed as described in (Yemini et al., 2019). Animals were mounted on 5% bacto-agar pad and immobilized in 50 mM NaN_3. Images were acquired using a LSM880 inverted point laser scanning confocal microscope equipped with Airyscan GaSaP detector, plan-apochromat 63x/1.4 oil objective and 405 nm 25 mW, argon 458/488/514 nm 30 mW, DPSS 561 nm 15 mW & HeNe 633 5mW lasers.

Spinning Disk Confocal Microscopy

Animals were mounted on 5% bacto-agar pad and immobilized in 50mM NaN_3. Images were acquired using a Visiscope Spinning Disc Confocal (Visitron Systems GmbH, Puching, Germany) with PCO Edge 4.2m sCMOS camera, CFI plan Apo lambda 100x/1.45 oil and 100% power of lasers 488 nm 200 mW and 561 nm 150 mW (300 ms exposure time for both). Z-stack space 0.5 um.

QUANTIFICATION AND STATISTICAL ANALYSIS

Statistical analyses were performed using the SciPy library for Python 3.0 (Virtanen et al., 2020). Differences in proportions were determined using the chi-squared test. Statistical details of the experiments can be found in the figure legends. All data are expressed as the proportion ± standard error of proportion (SEP).

Developmental Cell, Volume *55*

Supplemental Information

Combinatorial Action of Temporally

Segregated Transcription Factors

Julien Charest, Thomas Daniele, Jingkui Wang, Aleksandr Bykov, Ariane Mandlbauer, Mila Asparuhova, Josef Röhsner, Paula Gutiérrez-Pérez, and Luisa Cochella

Supplementary Information

Combinatorial action of temporally segregated transcription factors

Julien Charest[1], Thomas Daniele[1], Jingkui Wang[2], Aleksandr Bykov[2], Ariane Mandlbauer[2], Mila Asparuhova, Josef Röhsner, Paula Gutiérrez-Pérez and Luisa Cochella*

Research Institute of Molecular Pathology (IMP), Vienna BioCenter (VBC), Campus-Vienna-Biocenter 1, 1030 Vienna, Austria.

[1] Equal contribution

[2] Equal contribution

* Corresponding Author and Lead Contact: cochella@imp.ac.at

Supplementary Tables 1-5

Supplementary Figures 1-6

Supplementary Tables

	Heat shock T1				Heat shock T2			
	ASEL On	N	% ON	p-value	ASEL On	N	% ON	p-value
zif-1	78	84	93 ± 3	N/A	79	81	98 ± 2	N/A
vhhGFP4::zif-1	13	90	14 ± 4	<0.0001	81	85	95 ± 2	0.297

Table S1. ***lsy-6::yfp*** **expression in ASEL upon TBX-37 degradation (related to Figure 3)**

Number of animals scored, percentages and statistics for the experiments shown in **Fig. 3C**. YFP expression from the *lsy-6* locus was scored in the number of animals shown (N). YFP expression was either absent or observed exclusively in ASEL. Errors shown correspond to the standard error of the proportion. The proportions of animals expressing YFP were compared between experimental (nanobody-zif-1 line) and control (zif-1 alone) strains using a chi-squared test and p-values from these are shown for each of the two timepoints.

		lsy-6::gfp::Δtbs::5xUAS (*lucIs41*)				*lsy-6::gfp::5xUAS* (*lucIs54*)			
		ASEL On	N	%ON	p-value	ASEL On	N	%ON	p-value
		0	50	0 ± 0	N/A	50	50	100 ± 0	N/A
tbx-37p::gal4sk **(DBD alone)**	lucEx959	0	47	0 ± 0	N/A	50	50	100 ± 0	N/A
	lucEx960	0	60	0 ± 0	N/A				
	lucEx961	0	49	0 ± 0	N/A				
	lucEx962	0	54	0 ± 0	N/A				
tbx-37p::gal4sk::vp64 **(+ activator)**	lucEx937	61	67	91 ± 3	<0.0001				
	lucEx938	45	48	94 ± 3	<0.0001				
	lucEx939	44	51	86 ± 5	<0.0001				
	lucEx940	58	64	91 ± 4	<0.0001				
tbx-37p::gal4sk::unc-37 **(+ repressor)**	lucEx1024					3	44	7 ± 4	<0.0001
	lucEx1025					4	36	11 ± 5	<0.0001

Table S2. ***lsy-6::gfp*** **expression in ASEL upon GAL4-UAS tethering (related to Figure 5)**

Number of animals scored, percentages and statistics for the experiments shown in **Fig. 5B, C**. GFP expression from the *lsy-6* locus was scored in the number of animals shown (N) for each independent transgenic line. GFP expression was either absent or observed exclusively in ASEL. Errors shown correspond to the standard error of the proportion. The proportions of animals expressing GFP were compared using a chi-squared test and p-values from these are shown. The proportion in each independent experimental line (GAL4-VP64 or GAL4-UNC-37) was compared to tethering of the GAL4 DBD alone using the transgene *lucEx959*.

***lsy-2* Scoring**

	On	Dim	Off	N	% ON	% Dim	% OFF	p-value
otIs283 (lsy-6:yfp)	147	3	5	155	95 ± 2	2 ± 1	3 ± 1	N/A
otIs283, lsy-2 (ot64/+)	82	29	2	113	73 ± 4	26 ± 4	2 ± 1	<0.0001

delta Ebox Scoring

		On	Dim	Off	N	% ON	% Dim	% OFF	p-value
**wt Line 1**	**early emb**	24	0	2	26	92 ± 5	0 ± 0	8 ± 5	N/A
	**3-fold**	56	1	5	62	90 ± 4	2 ± 2	8 ± 3	N/A
**wt Line 2**	**early emb**	26	0	2	28	93 ± 25	0 ± 0	7 ± 25	N/A
	**3-fold**	58	0	0	58	100 ± 0	0 ± 0	0 ± 0	N/A
**ΔEbox Line 1**	**early emb**	16	3	3	22	73 ± 10	14 ± 7	14 ± 7	0.082
	**3-fold**	34	0	1	35	97 ± 3	0 ± 0	3 ± 3	0.210
**ΔEbox Line 2**	**early emb**	10	13	9	32	31.25	40.63	28.13	<0.0001
	**3-fold**	37	1	3	41	90.24	2.44	7.32	1
**ΔEbox Line 3**	**early emb**	18	12	8	38	47.37	31.58	21.05	0.0002
	**3-fold**	31	4	1	36	86.11	11.11	2.78	0.551

Table S3. *lsy-6::yfp* expression in ASEL in *lsy-2* mutants or upon E-box deletion (related to Figure 6)

Number of animals scored, percentages and statistics for the experiments shown in **Fig. 6B, C**. YFP expression from the *lsy-6* locus was scored in the number of animals shown (N). YFP expression was either absent or observed exclusively in ASEL. Errors shown correspond to the standard error of the proportion. The proportions of animals expressing YFP were compared using a chi-squared test and p-values from these are shown. Progeny of *lsy-2* heterozygous mothers were compared to wild-type animals carrying the same *lsy-6::yfp* fosmid reporter. For the E-box deletion experiment, the p-values for the comparisons between each independent experimental line (ΔEbox) to wild-type line 1 are shown (for each early embryos and 3-fold embryos); comparison to wild-type line 2 yielded lower p-values in all cases.

Table S4. Experiment Models: Organisms/Strains used in this study (related to STAR Methods and Key Resources Table)

Experimental Models: Organisms/Strains		
Caenorhabditis elegans: Wild type Bristol isolate	Caenorhabditis Genetics Center	WB Strain: N2
Caenorhabditis elegans: tbx-37(luc41[GFP::flex::tbx-37]) tbx-38(tm581) III	This study	MLC813
Caenorhabditis elegans: tbx-37(tm314) tbx-38(luc54[GFP::flex::tbx-38]) III	This study	MLC893
Caenorhabditis elegans: otIs252[lsy-6::YFP (fosmid); rol-6(su1006)] II; otIs220[gcy-5p::mChopti; rol-6(su1006)] IV	Hobert Lab Columbia University	OH9241
Caenorhabditis elegans: otIs220[gcy-5p::mChopti; rol-6(su1006)] IV; lsy-6(luc160[lsy-6::Δ150]) V	This study	MLC2219
Caenorhabditis elegans: lsy-6(luc157[lsy-6::YFP]), otIs235[che-1p::mChopti; rol-6(su1006)] V	This study	MLC2183
Caenorhabditis elegans: otIs232[che-1p::mChopti; rol-6] II; lsy-6(luc156[lsy-6::yfp::d150]) V	This study	MLC2311
Caenorhabditis elegans: lsy-6(luc157[lsy-6::YFP]), otIs235[che-1p::mChopti; rol-6(su1006)] V; otEx5161[hsp-16p::tbx-37; elt-2p::dsRed]	This study	MLC2203
Caenorhabditis elegans: lucSi100[hsp-16.41p::vhhGFP4::zif-1::SL2::mCherry::his-11::tbb-2 3'UTR] otIs252[lsy-6::YFP (fosmid); rol-6(su1006)] II; tbx-37(luc41[GFP::flex::tbx-37]) tbx-38(tm581) III	This study	MLC1300
Caenorhabditis elegans: lucSi102[hsp-16.41p::zif-1::SL2::mCherry::his-11::tbb-2 3'UTR] otIs252[lsy-6::YFP (fosmid); rol-6(su1006)] II; tbx-37(luc41[GFP::flex::tbx-37]) tbx-38(tm581) III	This study	MLC1219
Caenorhabditis elegans: otIs252[lsy-6::YFP (fosmid); rol-6(su1006)] II; tbx-37(tm314) tbx-38(tm581) III/qC1[dpy-19(e1259) glp-1(q339) qIs26[lag-2::GFP; rol-6(su1006)]	This study	MLC954
Caenorhabditis elegans: lucSi102[hsp-16.41p::zif-1::SL2::mCherry::his-11::tbb-2 3'UTR] II; wgIs37[pha-4::TY1::EGFP::3xFLAG; unc-119]	This study	MLC1467
Caenorhabditis elegans: lucSi100[hsp-16.41p::vhhGFP4::zif-1::SL2::mCherry::his-11::tbb-2 3'UTR] II; wgIs37[pha-4::TY1::EGFP::3xFLAG; unc-119]	This study	MLC1466
Caenorhabditis elegans: lucIs39[tbx-37p::mNeonGreen::2xNLS::tbx-37 3'UTR; pal-1p::mScarlet-I::2xNLS::tbb-2 3'UTR; med-2p::mScarlet-I::2xNLS::tbb-2 3'UTR]	This study	MLC1480
Caenorhabditis elegans: lucIs39[tbx-37p::mNeonGreen::2xNLS::tbx-37 3'UTR; pal-1p::mScarlet-I::2xNLS::tbb-2 3'UTR; med-2p::mScarlet-I::2xNLS::tbb-2 3'UTR)]; lsy-6(luc160[lsy-6::d150]) V	This study	MLC2309
Caenorhabditis elegans: otIs232[che-1p::mChopti; rol-6] II; lsy-6(luc160[lsy-6::d150]) V	This study	MLC2310

Caenorhabditis elegans: otIs252[lsy-6::YFP (fosmid); rol-6(su1006)] II	Caenorhabditis Genetics Center	WB Strain: OH8993
Caenorhabditis elegans: lucIs41[lsy-6::GFP::Δ150::5xUAS (fosmid); ttx-3p::mCherry]; lucEx959[tbx-37p::GAL4SK::tbx-37 3'UTR; elt-2p::mCherry]	This study	MLC1622
Caenorhabditis elegans: lucIs41[lsy-6::GFP::Δ150::5xUAS (fosmid); ttx-3p::mCherry]; lucEx960[tbx-37p::GAL4SK::tbx-37 3'UTR; elt-2p::mCherry]	This study	MLC1623
Caenorhabditis elegans: lucIs41[lsy-6::GFP::Δ150::5xUAS (fosmid); ttx-3p::mCherry]; lucEx961[tbx-37p::GAL4SK::tbx-37 3'UTR; elt-2p::mCherry]	This study	MLC1624
Caenorhabditis elegans: lucIs41[lsy-6::GFP::Δ150::5xUAS (fosmid); ttx-3p::mCherry]; lucEx962[tbx-37p::GAL4SK::tbx-37 3'UTR; elt-2p::mCherry]	This study	MLC1625
Caenorhabditis elegans: lucIs41[lsy-6::GFP::Δ150::5xUAS (fosmid); ttx-3p::mCherry]; lucEx937[tbx-37p::GAL4SK::VP64::tbx-37 3'UTR; elt-2p::mCherry]	This study	MLC1588
Caenorhabditis elegans: lucIs41[lsy-6::GFP::Δ150::5xUAS (fosmid); ttx-3p::mCherry]; lucEx938[tbx-37p::GAL4SK::VP64::tbx-37 3'UTR; elt-2p::mCherry]	This study	MLC1589
Caenorhabditis elegans: lucIs41[lsy-6::GFP::Δ150::5xUAS (fosmid); ttx-3p::mCherry]; lucEx939[tbx-37p::GAL4SK::VP64::tbx-37 3'UTR; elt-2p::mCherry]	This study	MLC1590
Caenorhabditis elegans: lucIs41[lsy-6::GFP::Δ150::5xUAS (fosmid); ttx-3p::mCherry]; lucEx940[tbx-37p::GAL4SK::VP64::tbx-37 3'UTR; elt-2p::mCherry]	This study	MLC1591
Caenorhabditis elegans: che-1(luc174) I; lucIs41[lsy-6p::GFP::d150::5xUAS; ttx-3p::mCherry] V; lucEx937[tbx-37p::GAL4SK::VP64; elt-2p::dsRed]	This study	MLC2320
Caenorhabditis elegans: che-1(luc174) I; lucIs41[lsy-6p::GFP::d150::5xUAS; ttx-3p::mCherry] V; lucEx938[tbx-37p::GAL4SK::VP64; elt-2p::dsRed]	This study	MLC2321
Caenorhabditis elegans: lucIs54[lsy-6::GFP::5xUAS (fosmid); ttx-3p::mCherry]; lucEx959[tbx-37p::GAL4SK::tbx-37 3'UTR; elt-2p::mCherry]	This study	MLC1920
Caenorhabditis elegans: lucIs54[lsy-6::GFP::5xUAS (fosmid); ttx-3p::mCherry]; lucEx1024[tbx-37p::GAL4SK::UNC-37::tbx-37 3'UTR; elt-2p::mCherry]	This study	MLC1741
Caenorhabditis elegans: lucIs54[lsy-6::GFP::5xUAS (fosmid); ttx-3p::mCherry]; lucEx1025[tbx-37p::GAL4SK::UNC-37::tbx-37 3'UTR; elt-2p::mCherry]	This study	MLC1742

Caenorhabditis elegans: lucIs41[lsy-6::GFP::Δ150::5xUAS (fosmid); ttx-3p::mCherry]	This study	MLC1557
Caenorhabditis elegans: lucIs54[lsy-6::GFP::5xUAS (fosmid); ttx-3p::mCherry]	This study	MLC1703
Caenorhabditis elegans: otIs386[lsy-6::GFP::Δ150 (fosmid); ttx-3p::mCherry]	Caenorhabditis Genetics Center	WB Strain: OH11115
Caenorhabditis elegans: unc-119(ed3) III; *wgIs159[tbx-2::TY1::eGFP::3xFLAG; unc-119]*	Caenorhabditis Genetics Center	WB Strain: OP159
Caenorhabditis elegans: unc-119(ed3) III; *wgIs311[tbx-7::TY1::eGFP::3xFLAG; unc-119]*	Caenorhabditis Genetics Center	WB Strain: OP311
Caenorhabditis elegans: lucEx552[tbx-8::GFP (fosmid); myo-2p::mCherry]	This study	MLC898
Caenorhabditis elegans: lucEx553[tbx-8::GFP (fosmid); myo-2p::mCherry]	This study	MLC899
Caenorhabditis elegans: unc-119(tm4063) III; *wgIs636[tbx-9::TY1::eGFP::3xFLAG; unc-119]*	Caenorhabditis Genetics Center	WB Strain: OP636
Caenorhabditis elegans: unc-119(tm4063) III; *wgIs368[tbx-11::TY1::eGFP::3xFLAG; unc-119]*	Caenorhabditis Genetics Center	WB Strain: OP368
Caenorhabditis elegans: lucEx822[mab-9p::MAB-9::T2A::GFP::H2B::mab-9 3'UTR; ttx-3p::mCherry]	This study	MLC1387
Caenorhabditis elegans: lucEx823[mab-9p::MAB-9::T2A::GFP::H2B::mab-9 3'UTR; ttx-3p::mCherry]	This study	MLC1388
Caenorhabditis elegans: lucEx824[mab-9p::MAB-9::T2A::GFP::H2B::mab-9 3'UTR; ttx-3p::mCherry]	This study	MLC1389
Caenorhabditis elegans: unc-119(ed3) III; *wgIs129[sea-1::TY1::eGFP::3xFLAG; unc-119]*	Caenorhabditis Genetics Center	WB Strain: OP129
Caenorhabditis elegans: lucEx818[tbx-31::GFP (fosmid); ttx-3p:mCherry]	This study	MLC1378
Caenorhabditis elegans: lucEx755[tbx-32::GFP (fosmid); ttx-3p::mCherry]	This study	MLC1276
Caenorhabditis elegans: lucEx628[tbx-33::GFP (fosmid); myo-2p:mCherry]	This study	MLC1019
Caenorhabditis elegans: lucEx757[tbx-33::GFP (fosmid); ttx-3p::mCherry]	This study	MLC1278
Caenorhabditis elegans: lucEx758[tbx-33::GFP (fosmid); ttx-3p::mCherry]	This study	MLC1279
Caenorhabditis elegans: lucEx825[tbx-34p::TBX-34::T2A::GFP::H2B::tbx-34 3'UTR; ttx-3p::mCherry]	This study	MLC1390
Caenorhabditis elegans: lucEx853[tbx-34p::TBX-34::T2A::GFP::H2B::tbx-34 3'UTR; ttx-3p::mCherry]	This study	MLC1435
Caenorhabditis elegans: lucEx756[tbx-35::GFP (fosmid); ttx-3p-mCherry]	This study	MLC1277
Caenorhabditis elegans: lucEx826[tbx-36p::TBX-36::T2A::GFP::H2B::tbx-36 3'UTR; ttx-3p::mCherry]	This study	MLC1391
Caenorhabditis elegans: lucEx827[tbx-36p::TBX-36::T2A::GFP::H2B::tbx-36 3'UTR; ttx-3p::mCherry]	This study	MLC1392
Caenorhabditis elegans: lucEx935[tbx-39p::TBX-39::T2A::GFP::H2B::tbx-39 3'UTR; ttx-3p::mCherry]	This study	MLC1578
Caenorhabditis elegans: lucEx936[tbx-39p::TBX-39::T2A::GFP::H2B::tbx-39 3'UTR; ttx-3p::mCherry]	This study	MLC1579

Caenorhabditis elegans: lucEx883[tbx-43p::TBX-43::T2A::GFP::H2B::tbx-43 3'UTR; ttx-3p::mCherry]	This study	MLC1493
Caenorhabditis elegans: lucEx893[tbx-43p::TBX-43::T2A::GFP::H2B::tbx-43 3'UTR; ttx-3p::mCherry]	This study	MLC1506
Caenorhabditis elegans: lsy-6(ot150) V; nre-1(hd20) lin-15b(hd126) X; otIs186[gcy-5p::GFP; rol-6(su1006)]	Hobert Lab Columbia University	OH8996
Caenorhabditis elegans: otIs252[lsy-6::YFP (fosmid); rol-6(su1006)] II; tbx-11(luc144) III	This study	MLC1977
Caenorhabditis elegans: otIs252[lsy-6::YFP (fosmid); rol-6(su1006)] II; tbx-33(gk3098) /hT2[qIs48[myo-2::GFP; pes-10::GFP; F22B7.9::GFP] III	This study	MLC1928
Caenorhabditis elegans: otIs252[lsy-6::YFP (fosmid); rol-6(su1006)] II; tbx-43(luc131) III; otIs220[gcy-5p::mCherry; rol-6(su1006)] IV	This study	MLC1866
Caenorhabditis elegans: otIs252[lsy-6::YFP (fosmid); rol-6(su1006)] II; otIs235[che-1p::mChopti; rol-6(su1006)] V	This study	MLC2239
Caenorhabditis elegans: otIs252[lsy-6::YFP; rol-6(su1006)] II; tbx-37(tm314) tbx-38(tm581)/qC1[dpy-19(e1259) glp-1(q339) qIs26] III; lucEx1191[che-1p::mChopti; unc-122p::RFP]	This study	MLC2180
Caenorhabditis elegans: tbx-37(tm314) tbx-38(tm581)/qC1[dpy-19(e1259) glp-1(q339) qIs26] III; lucEx1179[lsy-6p::GFP::Δ150::5xUAS (fosmid); che-1p::mChopti; tbx-37p::GAL4SK::VP64; unc-122p::RFP]	This study	MLC2110
Caenorhabditis elegans: tbx-37(tm314) tbx-38(tm581)/qC1[dpy-19(e1259) glp-1(q339) qIs26] III, lucEx1180[lsy-6p::GFP::Δ150::5xUAS (fosmid); che-1p::mChopti; tbx-37p::GAL4SK::VP64; unc-122p::RFP]	This study	MLC2111
Caenorhabditis elegans: tbx-37(tm314) tbx-38(tm581)/qC1[dpy-19(e1259) glp-1(q339) qIs26] III, lucEx1181[lsy-6p::GFP::Δ150::5xUAS (fosmid); che-1p::mChopti; tbx-37p::GAL4SK::VP64; unc-122p::RFP]	This study	MLC2112
Caenorhabditis elegans: otIs283[lsy-6::YFP (fosmid); rol-6(su1006)] V; lsy-2(ot64)/+ X	Hobert Lab Columbia University	OH9593
Caenorhabditis elegans: otEx4375[lsy-6::YFP (fosmid); ttx-3p::mCherry]	Hobert Lab Columbia University	OH9863
Caenorhabditis elegans: otEx4376[lsy-6::YFP (fosmid); ttx-3p::mCherry]	Hobert Lab Columbia University	OH9864
Caenorhabditis elegans: otEx4379[lsy-6::YFP::ΔEbox (fosmid); ttx-3p::mCherry]	Hobert Lab Columbia University	OH9867
Caenorhabditis elegans: otEx4380[lsy-6::YFP::ΔEbox (fosmid); ttx-3p::mCherry]	Hobert Lab Columbia University	OH9868
Caenorhabditis elegans: otEx4381[lsy-6::YFP::ΔEbox (fosmid); ttx-3p::mCherry]	Hobert Lab Columbia University	OH9869

Caenorhabditis elegans: otIs235[che-1p::mChopti; rol-6(su1006)] V; lucEx881[C32C4.16::T2A::GFP::H2B; unc-122p::RFP]	This study	MLC2240
Caenorhabditis elegans: lucEx1129[C32C4.16::T2A::GFP::H2B (fosmid); opt-3p::tagRFP; unc-122p::RFP]	This study	MLC1976
Caenorhabditis elegans: lucEx1130[C32C4.16::T2A::GFP::H2B (fosmid); flp-8p::mCherry; unc-122p::RFP]	This study	MLC1978
Caenorhabditis elegans: lucEx881[C32C4.16::T2A::GFP::H2B (fosmid); unc-122p::RFP]	This study	MLC1491
Caenorhabditis elegans: tbx-37(tm314) tbx-38(tm581)/qC1[dpy-19(e1259) glp-1(q339) qIs26[lag-2::GFP; rol-6(su1006)]] III, *lucEx881 [C32C4.16::T2A::GFP::H2B (fosmid); unc-122p::RFP]*	This study	MLC1961
Caenorhabditis elegans: otIs235[che-1p::mChopti; rol-6(su1006)] V; *lucEx1176[lsy-6::Δ150::C32C4.16::T2A::GFP::H2B (fosmid); unc-122p::RFP]*	This study	MLC2104
Caenorhabditis elegans: otIs670[NeuroPal] V, *lucEx881[C32C4.16::T2A::GFP::H2B (fosmid); unc-122p::RFP]*	This study	MLC1962

Table S5. Recombinant DNA used in this study (related to STAR Methods and Key Resources Table)

Recombinant DNA		
Fosmid:<br>pCC1 - *tbx-8::GFP* (WRM065aG10)	This study	N/A
Fosmid:<br>pCC1 - *tbx-31::GFP* (WRM067dD02)	This study	N/A
Fosmid:<br>pCC1 - *tbx-32::GFP* (WRM0641aB04)	This study	N/A
Fosmid:<br>pCC1 - *tbx-33::GFP* (WRM0619bA09)	This study	N/A
Fosmid:<br>pCC1 - *tbx-35::GFP* (WRM0619dE06)	This study	N/A
Fosmid:<br>pCC1 - *lsy-6::GFP::Δ150::5xUAS* (WRM0628bA07)	This study	N/A
Fosmid:<br>pCC1 - *lsy-6::GFP::5xUAS* (WRM0628bA07)	This study	N/A
Fosmid:<br>pCC1 - *C32C4.16::T2A::GFP::H2B* (WRM0628bA07)	This study	N/A
Fosmid:<br>pCC1 - *lsy-6::Δ150::C32C4.16::T2A::GFP::H2B* (WRM0628bA07)	This study	N/A
Plasmid:<br>puc19 - *tbx-37::GFP::FLEX*	This study	N/A
Plasmid:<br>puc19 - *tbx-38::GFP::FLEX*	This study	N/A
Plasmid:<br>pCFJ350 – *hsp-16.41p::ZIF-1::SL2::mCherry::his-11::tbb-2 3'UTR*	This study	N/A
Plasmid:<br>pCFJ350 – *hsp-16.41p::vhhGFP4::ZIF-1::SL2::mCherry::his-11::tbb-2 3'UTR*	This study	N/A
Plasmid:<br>psC-B-AmpKan - *tbx-37p::mNeonGreen::2xNLS::tbx-37 3'UTR*	This study	N/A
Plasmid:<br>puc19 - *pal-1p::mScarlet-I::2xNLS::tbb-2 3'UTR*	This study	N/A
Plasmid:<br>puc19 - *med-2p::mScarlet-I::2xNLS::tbb-2 3'UTR*	This study	N/A
Plasmid:<br>psC-B-AmpKan – *tbx-34p::tbx-34::T2A::GFP::H2B::tbx-34 3'UTR*	This study	N/A
Plasmid:<br>psC-B-AmpKan – *tbx-36p::tbx-36::T2A::GFP::H2B::tbx-36 3'UTR*	This study	N/A
Plasmid:<br>psC-B-AmpKan – *tbx-39p::tbx-39::T2A::GFP::H2B::tbx-39 3'UTR*	This study	N/A

Plasmid:<br>psC-B-AmpKan – *tbx-43p::tbx-43::T2A::GFP::H2B::tbx-43 3’UTR*	This study	N/A
Plasmid:<br>psC-B-AmpKan – *tbx-37p::GAL4SK::tbx-37 3'UTR*	This study	N/A
Plasmid:<br>psC-B-AmpKan – *tbx-37p::GAL4SK::VP64::tbx-37 3'UTR*	This study	N/A
Plasmid:<br>psC-B-AmpKan – *tbx-37p::GAL4SK::UNC-37::tbx-37 3'UTR*	This study	N/A
Plasmid:<br>pSM – *myo-2p::mCherry*	Zimmer lab<br>University of Vienna	N/A
Plasmid:<br>pPD – *ttx-3p::mCherry*	Hobert Lab<br>Columbia University	N/A
Plasmid:<br>pPD – *elt-2p::dsRED*	Hobert Lab<br>Columbia University	N/A
Plasmid:<br>pPD – *unc-122p::RFP*	Hobert Lab<br>Columbia University	N/A
Plasmid:<br>pSM – *flp-8p::mCherry*	Zimmer lab<br>University of Vienna	N/A
Plasmid:<br>pSM – *opt-3p::tagRFP*	Zimmer lab<br>University of Vienna	N/A
Plasmid:<br>pPD – *che-1p::mChopti*	Hobert Lab<br>Columbia University	N/A
Plasmid:<br>pL440 control RNAi	Kamath & Ahringer, 2003	N/A
Plasmid:<br>pL440 – *mab-9* RNAi	Kamath & Ahringer, 2003	*T27A1.6*
Plasmid:<br>pL440 – *mls-1* RNAi	Kamath & Ahringer, 2003	*H14A12.4*
Plasmid:<br>pL440 – *sea-1* RNAi	Kamath & Ahringer, 2003	*F19B10.9*
Plasmid:<br>pL440 – *tbx-2* RNAi	Kamath & Ahringer, 2003	*F21H11.3*
Plasmid:<br>pL440 – *tbx-7* RNAi	Kamath & Ahringer, 2003	*ZK328.8*
Plasmid:<br>pL440 – *tbx-8* RNAi	Kamath & Ahringer, 2003	*T07C4.2*
Plasmid:<br>pL440 – *tbx-9* RNAi	Kamath & Ahringer, 2003	*T07C4.6*
Plasmid:<br>pL440 – *tbx-11* RNAi	Kamath & Ahringer, 2003	*F40H6.4*
Plasmid:<br>pL440 – *tbx-30* RNAi	This study	N/A
Plasmid:<br>pL440 – *tbx-31* RNAi	Kamath & Ahringer, 2003	*C36C9.2*
Plasmid:<br>pL440 – *tbx-32* RNAi	This study	N/A
Plasmid:<br>pL440 – *tbx-33* RNAi	Kamath & Ahringer, 2003	*Y66A7A.8*

Plasmid:<br>pL440 – *tbx-34* RNAi	This study	N/A
Plasmid:<br>pL440 – *tbx-35* RNAi	This study	N/A
Plasmid:<br>pL440 – *tbx-36* RNAi	Kamath & Ahringer, 2003	*ZK829.5*
Plasmid:<br>pL440 – *tbx-37* RNAi	This study	N/A
Plasmid:<br>pL440 – *tbx-38* RNAi	Kamath & Ahringer, 2003	*C24H11.3*
Plasmid:<br>pL440 – *tbx-39* RNAi	This study	N/A
Plasmid:<br>pL440 – *tbx-40* RNAi	This study	N/A
Plasmid:<br>pL440 – *tbx-41* RNAi	This study	N/A
Plasmid:<br>pL440 – *tbx-42* RNAi	Kamath & Ahringer, 2003	*Y59E9AR.5*
Plasmid:<br>pL440 – *tbx-43* RNAi	This study	N/A

Supplementary Figures

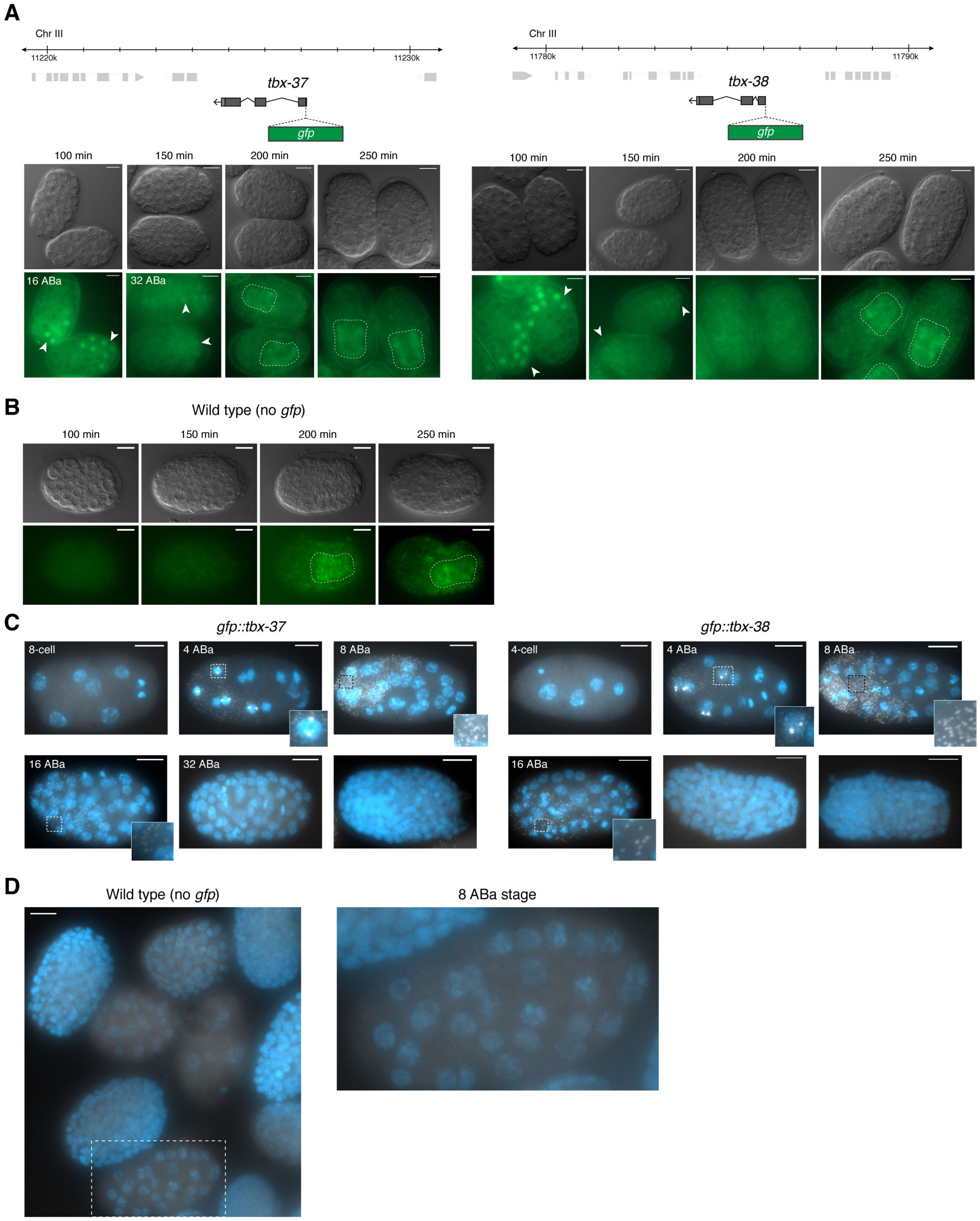

Figure S1. TBX-37/38 are transiently expressed (related to Figures 2 and 3)
A. Schematic of the CRISPR engineered *tbx-37* and *tbx-38* loci and GFP expression from embryos carrying these alleles. DIC (single plane) and GFP fluorescence (max. intensity projections) are shown. Embryos were collected at the 2-cell stage and allowed to develop for the indicated times at 20ºC. Arrowheads point to nuclei with visible GFP signal. At later timepoints the developing gut displays autofluorescence and is delimited by the dashed line. All scale bars represent 10 µm. **B.** Representative images of wt, non-transgenic embryos at the same timepoints as in A to show autofluorescence levels (max. intensity projections). **C.** Analysis of transcription from the *gfp::tbx-37* and *gfp::tbx-38* loci by smFISH with probes against *gfp.* The smFISH signal is shown in white and DAPI staining in blue (max. intensity projections), both signals are overlayed. Embryos were staged by counting DAPI-stained nuclei. Insets show the onset of transcription at the 4 ABa stage, then mRNA in the cytoplasm peaking at the 8 ABa stage and then rapidly disappearing. All scale bars represent 10 µm. **D.** Negative control for smFISH staining, on wild-type, non-transgenic embryos

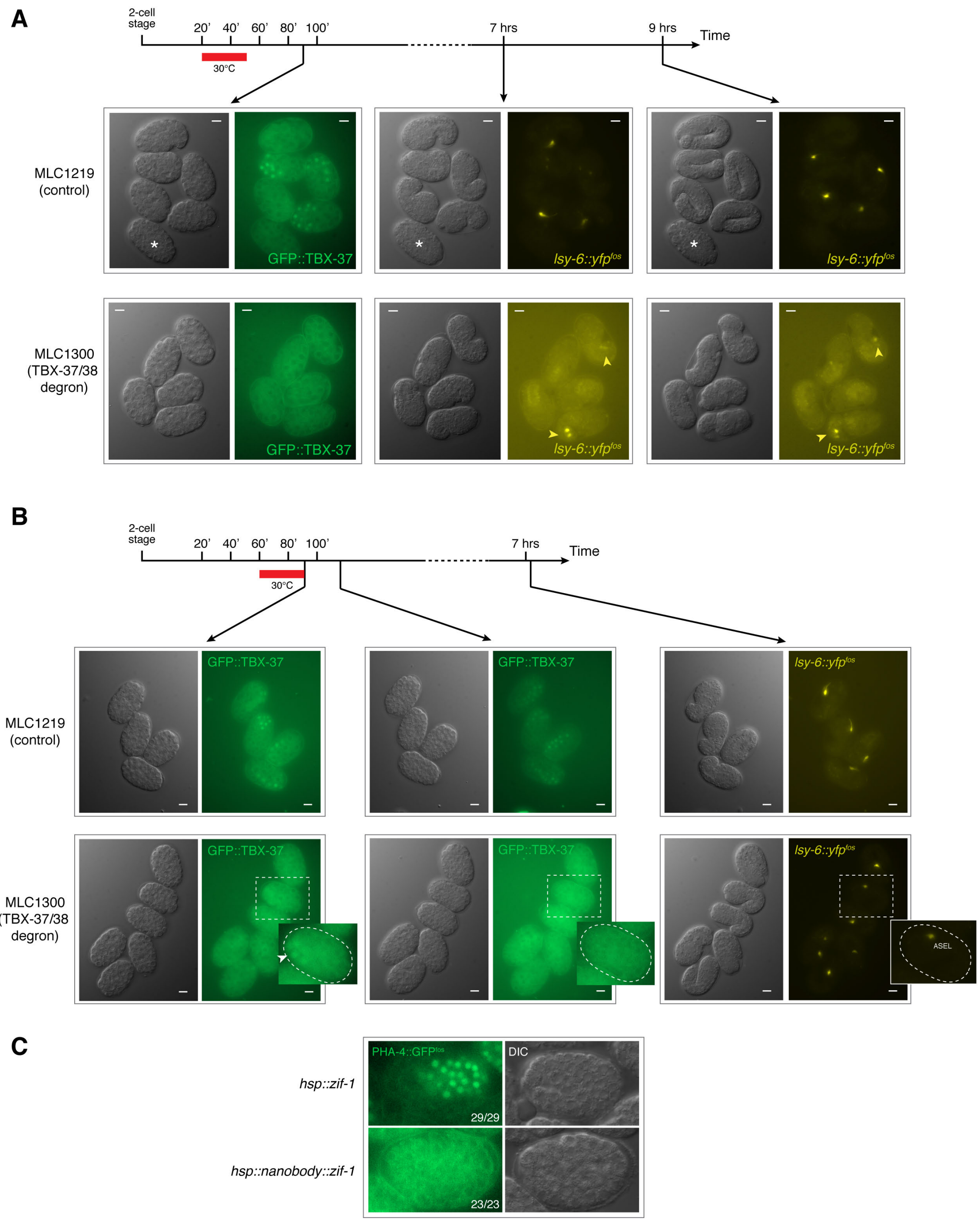

Figure S2. Experimental design and representative images for the TBX-37 degron experiment (related to Figure 3)

A. Timescale of the forced degradation experiment in Figure 3. Activation of the degron at the first time point (t1 = 20-50 minutes) resulted in robust degradation of GFP::TBX-37 (in a *tbx-38(0)* background), at its peak of expression (compare GFP::TBX-37 signal in control vs. degron at 90 min). This degradation prevented *lsy-6::yfp* activation (compare YFP signal in control vs. degron at 7 and 9 hours) and phenocopied the *tbx-37/38(0)* mutant morphology (compare DIC images). The arrowheads point to ectopic expression of *lsy-6::yfp* in a pair of cells in the tail of the embryo that we sometimes observe after heat shock treatment. The asterisk marks an embryo that arrested and died early in the experiment. **B.** Activation of the degron at the second time point (t2 = 60-90 minutes) already caused lower GFP::TBX-37 signal at the 90 min timepoint but we observed loss of signal 30 min later (120 min timepoint). This robust degradation of GFP::TBX-37 (in a *tbx-38(0)* background) slightly after their peak of expression did not affect *lsy-6::yfp* activation nor did it cause morphological defects as in the *tbx-37/38(0)* mutant. **C.** Activation of the degron at the second timepoint (t2 = 60-90 min) is able to clear PHA-4::GFP, indicating that the system is able to efficiently degrade a highly-expressed nuclear protein at this time point. The observed green signal in the degron strain is autofluorescence, different from the nuclear localized PHA-4::GFP signal.

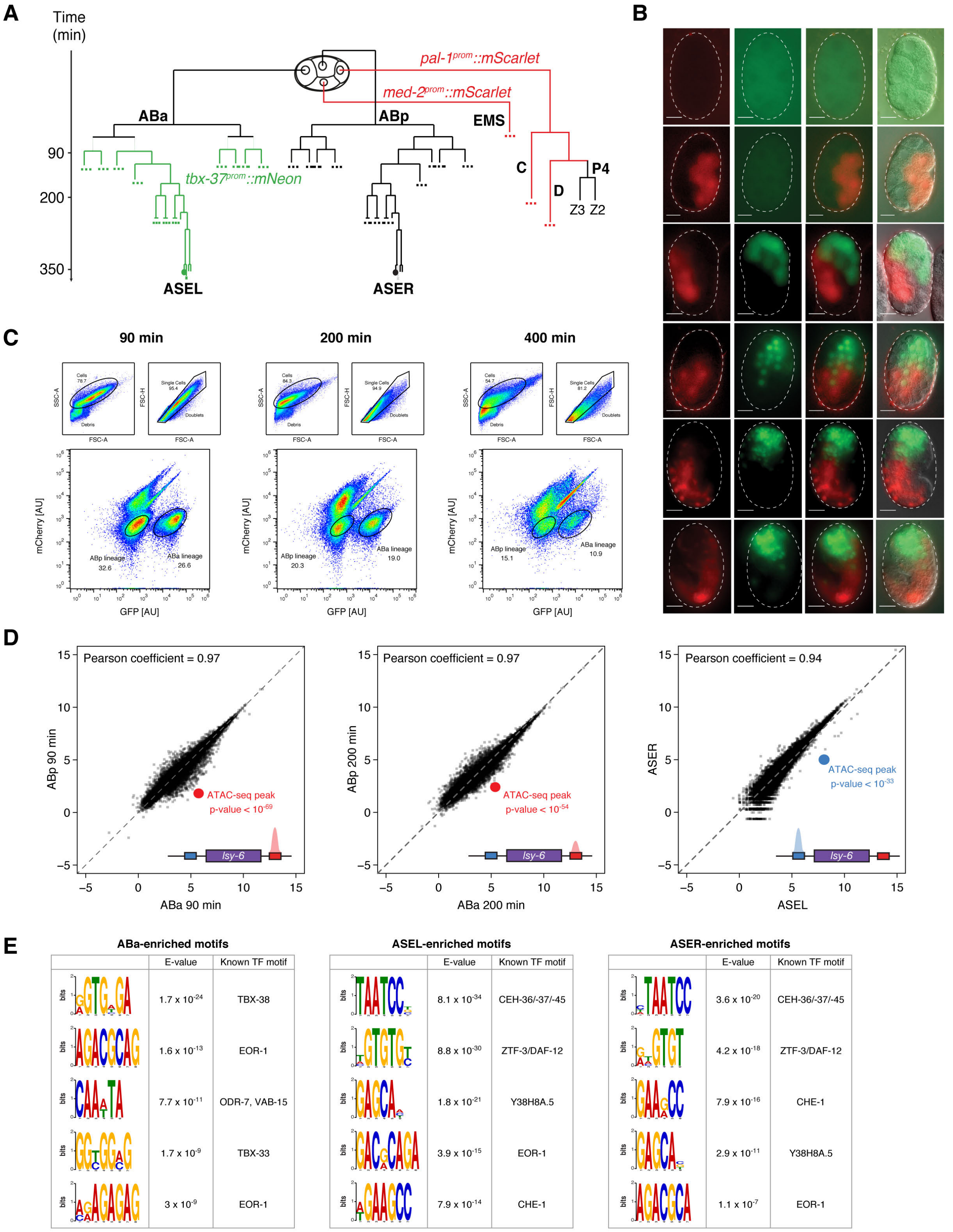

Figure S3. Experimental design for lineage specific FACS (related to Figure 4)

A. Schematic of the embryo labeling strategy that enables separation of ABa and ABp derived cells by FACS. Descendants from the ABa blastomere are labeled with a *tbx-37^{prom}::mNeonGreen* and cells from EMS and C/D lineages are labeled with *med-2^{prom}::mScarlet* and *pal-1^{prom}::mScarlet*, respectively. The Z2 and Z3 cells also remain unlabeled, but they represent a very minor fraction compared to the rest of the ABp lineage. **B.** Representative images following the development of a labeled embryo over time. DIC and fluorescence images are shown. From top to bottom, 6-cell, 8-10-cell, 8 ABa, 16 ABa, comma and 1.5-fold stages. Scale bars = 10 µm.

C. FACS plot representation of the ABa (GFP positive) and ABp (GFP and mScarlet negative) sorted cell population for the three ATAC-seq time points. FACS pseudo-colored plots for cells dissociated from the described strain at different timepoints. From left to right, 90 min, 200 min and 400 min. The top panels show the gating used to separate single cells from debris and doublets or higher-order cell aggregates. The bottom panel shows the separation of GFP positive, mScarlet negative cells corresponding to the ABa lineage; and double negative cells corresponding to ABp descendants. The cell populations outlined in black were isolated by FACS for the preparation of ATAC-seq libraries. **D.** Pairwise comparisons of ATAC-seq signal, in $\log_2$ (cpm), for all peaks called by MACS in at least one ATAC-seq sample. Pearson's correlation coefficients are shown. The p-values (MACS2) for the called *lsy-6* peaks in ABa and ASEL are shown. **E.** De novo motif discovery using the MEME Suite was used to find enriched motifs (and predicted TFs that can bind them) in ABa (using peaks detected in ABa at 90 min that do not overlap with peaks in ABp at 90 min), ASEL (using peaks detected in ASEL that do not overlap with peaks in ABa at 350 min) and ASER (using peaks detected in ASER that do not overlap with peaks in ABp at 350 min). ABa-specific accessible regions have significant enrichment of TBX-37/38 binding sites, while both ASEL and ASER accessible regions have significant enrichment of motifs that correspond to CHE-1 binding consensus and other TFs.

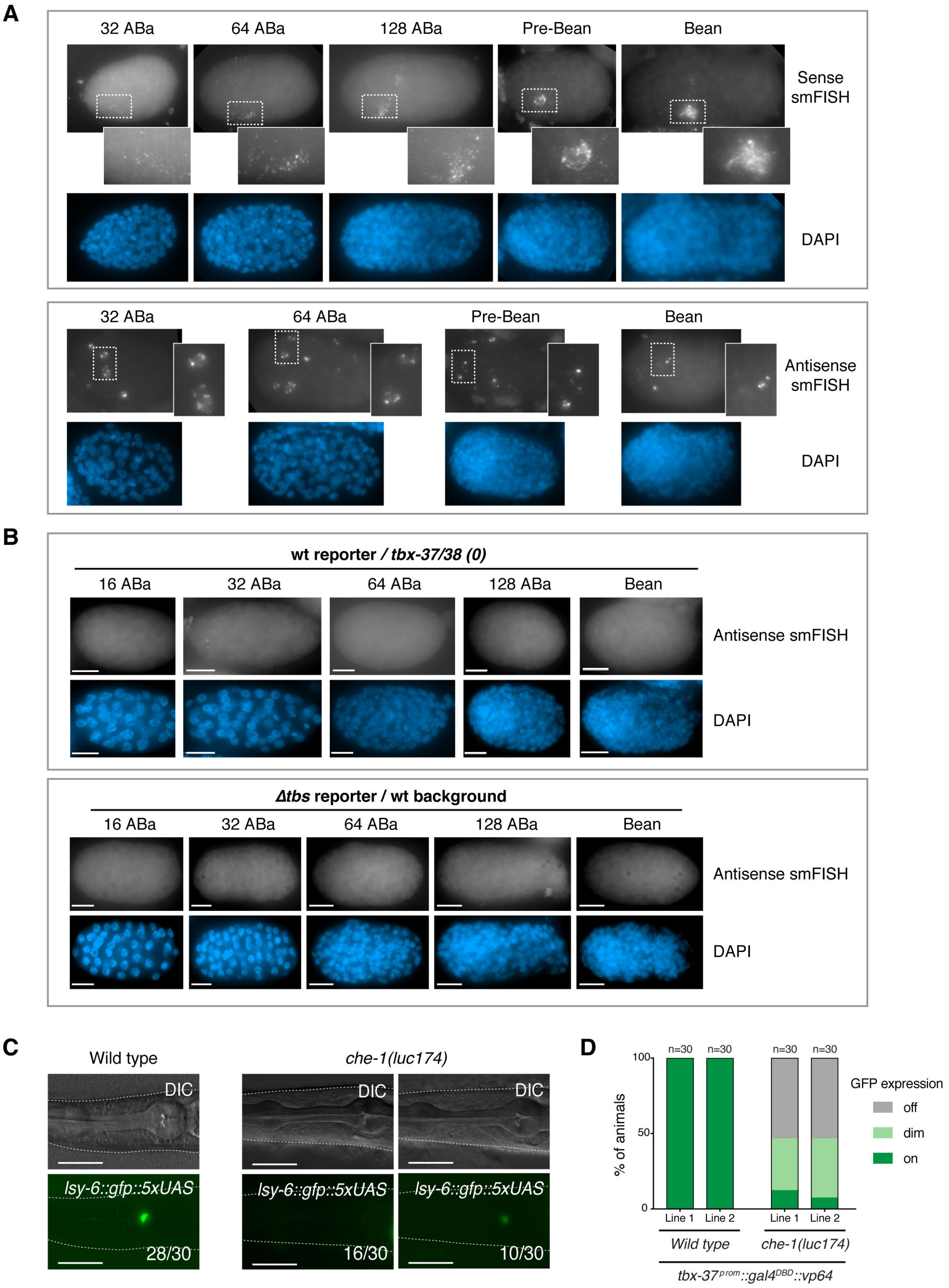

Figure S4. Transcription of the *lsy-6* locus occurs bidirectionally and requires TBX-37/38 binding (related to Figure 5)
A. Single molecule in situ hybridization on embryos carrying the *lsy-6::yfp*^{fosmid} with probes against the sense *yfp* transcript show low levels of sense transcription of the *lsy-6* locus from 32 AB stage until boosting of expression by CHE-1 in the ASEL mother cell (Pre-bean). Embryos were staged by counting DAPI-stained nuclei. Dashed boxes indicate zoomed regions showing bright nuclear foci. (Bottom) Same as above but using a probe set against the antisense of *yfp.* The insets show the presence of bright nuclear foci, indicative of continuous transcription. **B.** Antisense transcription of *lsy-6::yfp* is fully dependent on TBX-37/38 (top) and its binding sites (bottom). **C.** (Left) Representative image of animals like the ones scored in Fig. 5B, in which tethering of GAL4-VP64 restores *lsy-6::gfp::Δtbs::5xUAS* expression in ASEL. (Right) Representative images of the same tethering experiment in a *che-1*-deficient background. **D.** Scoring for the experiment described in C. Two independent lines were scored.

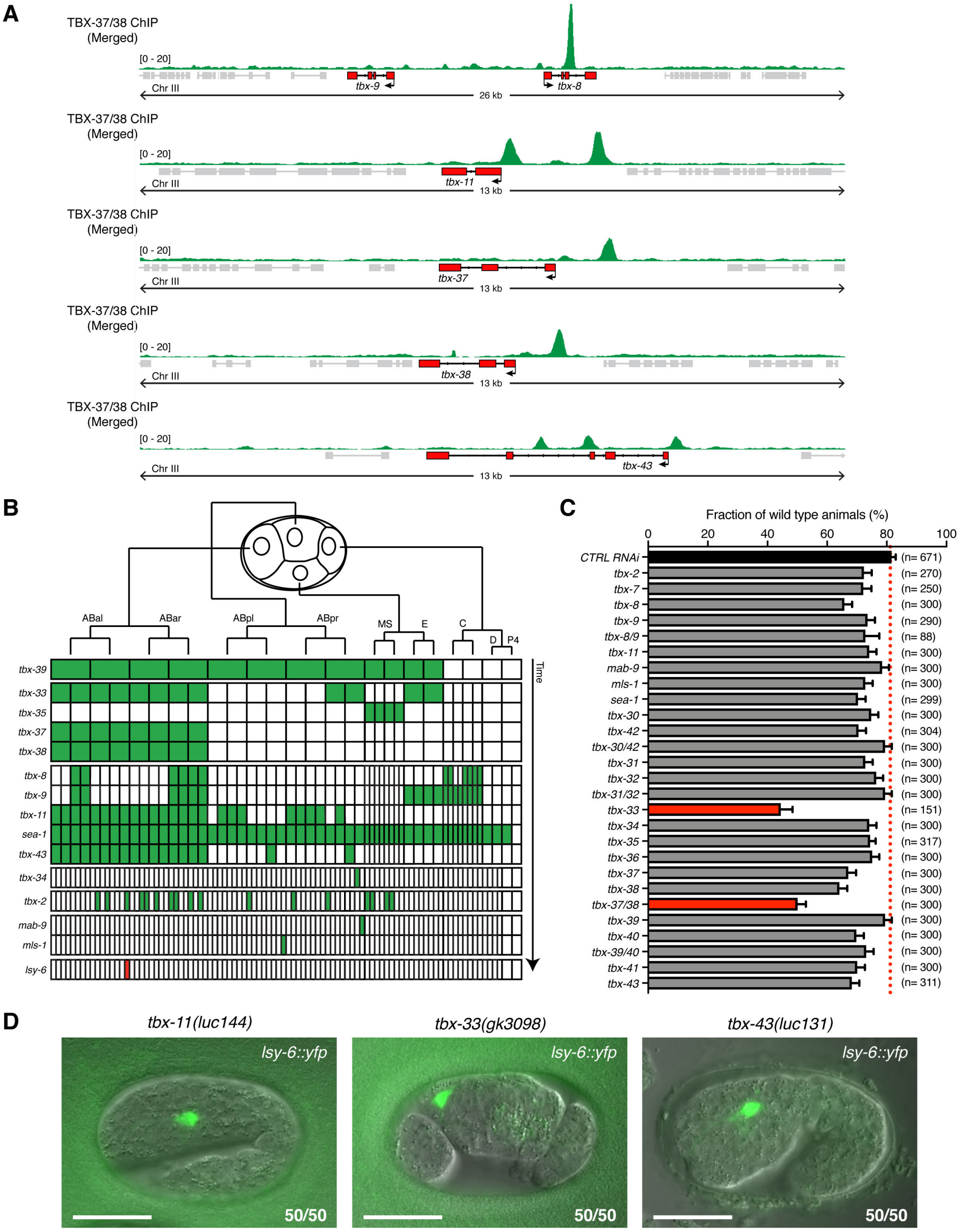

Figure S5. TBX-37/38 action at the *lsy-6* locus is not relayed by other T-Box transcription factors (related to Figure 6)

A. Aggregated GFP-TBX-37/38 ChIP-seq signal over T-box gene loci. TBX-37/38 were found to bind in the vicinity of *tbx-8/9*, *tbx-11*, *tbx-37*, *tbx-38* and *tbx-43*. **B.** Reporters for most *tbx* genes were generated and examined for expression. Those that showed early embryonic expression were lineaged with the aid of 4D microscopy and Simi Biocell software. The onset of expression of those early-expressed *tbx* genes is represented as a green box in the matrix. Boxes get narrower with every cell division. Expression of *tbx-33*, *tbx-37*, *tbx-38*, *tbx-11*, *sea-1*, *tbx-43* and *tbx-2* was observed in the ABalpp lineage branch, which will give rise to ASEL. **C.** Effect of RNA interference against different *tbx* genes on *lsy-6* expression in a sensitized background. A point mutation in the CHE-1 binding site in the promoter of *lsy-6* (*ot150*) affects CHE-1 binding efficiency and results in a sensitized background in which 20% of animals fail to express enough *lsy-6* and to specify ASEL (Sarin et al., 2007). The failure to specify ASEL is monitored by its ectopic expression of the ASER terminal fate marker *gcy-5^{prom}::GFP*. Because some of these T-box factors occur in recently duplicated pairs, we also tested for possible redundancy by doing double RNAi. Only RNAi against *tbx-37/38* or against *tbx-33* caused further enhancement of the defect. The effect of *tbx-33* was further explored by using a deletion allele (panel D) but could not be validated. **D.** Expression of the *lsy-6::yfp* fosmid reporter is not affected by deletion of *tbx-11, tbx-33* or *tbx-43*. All scale bars represent 20 μm.

A

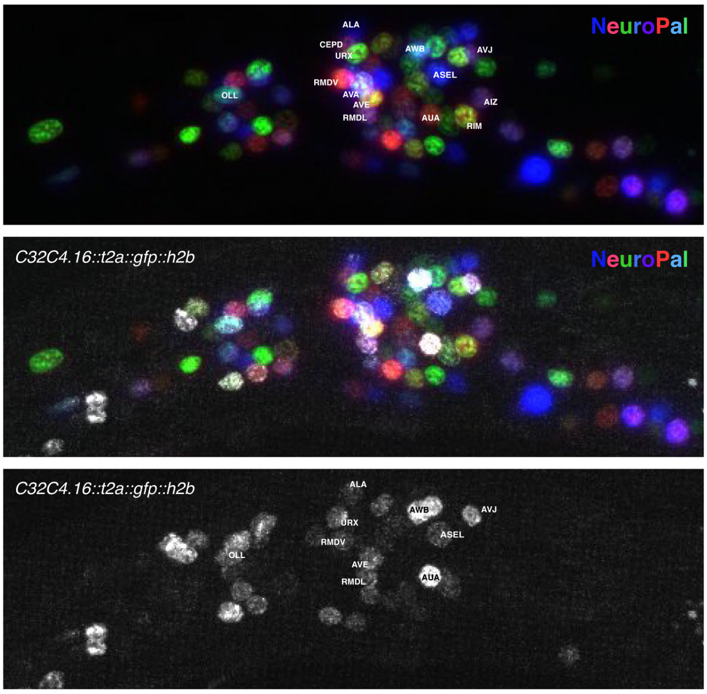

B

Worm	Orientation	Identified Neurons
1	Left	AUAL, AWBL, AVEL, RMDL, ASEL, AVJL/R, RMDV
2	Left	AVEL, RMDL, ASEL, OLLL
3	Left	AUAL, AWBL, AVEL, RMDL, ASEL, AVJL/R, RMDV, SMDVL/R, RMDD
4	Right	AUAL, AWBL, AVEL, RMDL, ASEL, AVJL/R, RMDVL/R, SMDVL/R, RMDDR, AVAR

C

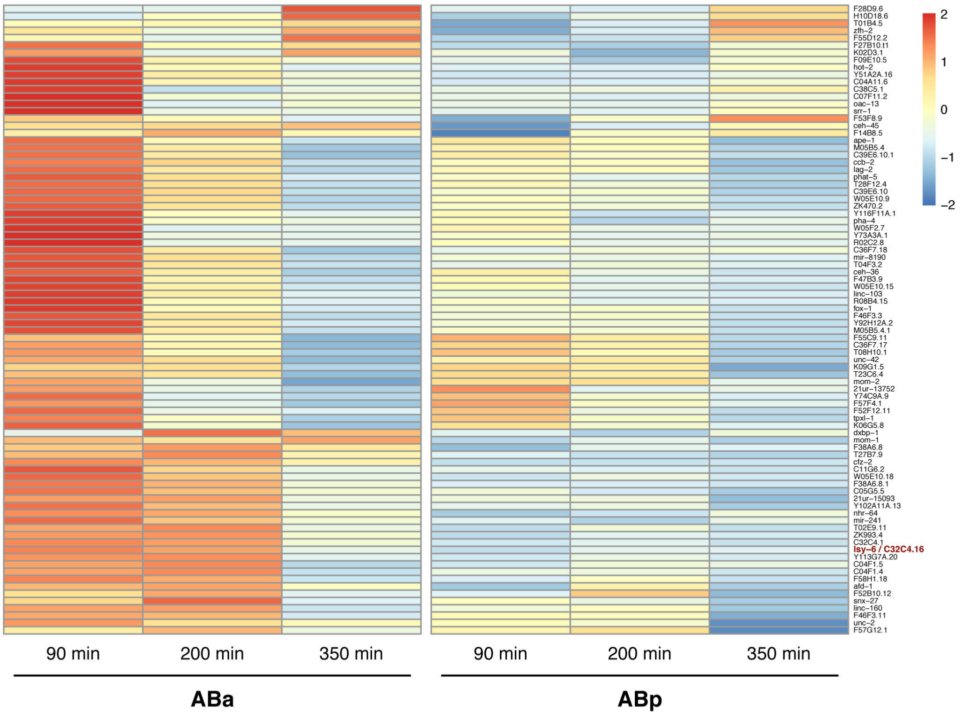

Figure S6. Identification of neurons expressing C32C4.16 and of candidate genes primed by TBX-37/38 (related to Figure 7)

A. Representative images of C32C4.16 reporter in the NeuroPal system, which uses a combination of over 40 neuron-specific promoters to allow easy neuronal identification by their unique color combination and position (Yemini et al., 2019). Shown are the neuronal identity assignments based on these properties (top), the nuclear GFP-H2B signal from the C32C4.16 reporter (bottom) and an overlay of both (middle). Max. intensity projections from one animal are shown. **B.** Four L1-stage larvae were analyzed in detail using this system. The neurons we could confidently identify are listed. In all four cases we could identify the right-side counterparts of AUAL, AWBL, AVEL, RMDL and ASEL, but we never saw GFP expression in those cells. **C.** Heatmap showing the scaled ATAC-seq accessibility of 86 genes that harbor TBX-37/38 binding sites (by ChIP-seq) and are asymmetrically accessible in ABa at 90 min, but not in ABp. The regulatory region between *lsy*-6 and C32C4.16 are highlighted in red. These genes represent candidates for regulation similar to *lsy-6.*
